# Lactoferrin Deficiency During Lactation Causes Adult Obesity‐Related Metabolic Disease Through Persistent Adipose Dysfunction Driven by Impaired Adipocyte Development

**DOI:** 10.1002/advs.75678

**Published:** 2026-05-19

**Authors:** Qin An, Yunxia Zou, Wenli Wang, Zhimei Cheng, Zhuoxing Zhang, Ruwei Liu, Xiong Wang, Kunlun Huang, Fangrong Ding, Yunping Dai, Qingyong Meng, Yali Zhang

**Affiliations:** ^1^ College of Food Science and Nutritional Engineering China Agricultural University Beijing China; ^2^ Shanghai Key Laboratory of Embryo Original Diseases The International Peace Maternity and Child Health Hospital Shanghai Jiao Tong University School of Medicine Shanghai China; ^3^ College of Biological Sciences China Agricultural University Beijing China

**Keywords:** adipocyte, adipose tissue plasticity, lactoferrin, metabolic programming, obesity, single‐nucleus RNA sequencing

## Abstract

Obesity is associated with metabolic disorders due to unhealthy white adipose tissue (WAT) failing to sustain energy homeostasis, highlighting the importance of adipose development. The lactation period is critical for epididymal WAT (eWAT) development and metabolic programming. However, the role of lactoferrin (LF) in the early development of adipose remains unclear. Using a mouse model of lactational LF deficiency and single‐nucleus RNA sequencing, we assessed the long‐term impact of LF deficiency on eWAT plasticity and metabolic homeostasis at weaning, adulthood, and under a high‐fat diet (HFD). LF deficiency persistently impaired eWAT development, causing restricted adipocyte hyperplasia, exacerbated hypertrophy, diminished lipid uptake, and sustained adiponectin decline with resistin elevation. These defects led to long‐term metabolic disorders, worsening HFD‐induced eWAT remodeling, glucose intolerance, dyslipidemia, and chronic inflammation. Mechanistically, LF could bind CSK and PRMT5. LF promoted CSK degradation, activating SRC to drive preadipocyte proliferation. Additionally, LF stabilized PRMT5 to enhance PPARg‐mediated differentiation and lipid uptake. Rescue experiments confirmed that CSK overexpression reversed LF‐induced proliferation, while PRMT5 knockdown blocked LF‐enhanced differentiation. This study reveals lactational LF as a key nutritional signal that programs adipose development and long‐term metabolic health via CSK‐SRC and PRMT5‐PPARg pathways, offering an early‐life intervention strategy against obesity‐related metabolic diseases.

## Graphical abstract

Lactational lactoferrin deficiency exerts lasting effects on epididymal adipose tissue development from lactation into adulthood: it impairs adipocyte hyperplasia and induces pathological hypertrophy, resulting in lower body weight yet exacerbated metabolic dysfunction under a high‐fat diet in adulthood. Mechanistically, lactoferrin drives adipocyte proliferation via the CSK‐SRC pathway and promotes healthy adipose development through PRMT5‐PPARG signaling.

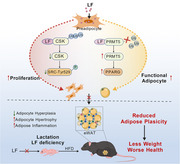

## 1 Introduction

Obesity is a global epidemic and serves as a major risk factor for type 2 diabetes, cardiovascular diseases, cancer, and non‐alcoholic fatty liver disease [1, 2]. Its pathogenesis primarily stems from a chronic imbalance between energy intake and expenditure, leading to pathological fat accumulation and systemic metabolic dysregulation [3]. It is well known that excessive fat levels are closely related to a higher incidence of metabolic diseases, however, there are significant differences among individuals [4]. For instance, some obese people can maintain metabolic health, while some lean people suffer from metabolic diseases [5]. Similarly, although patients with atrophy of fat have a small amount of fat tissue, they suffer from many of the same diseases as those with severe obesity [6]. Growing evidence identifies adipose tissue dysfunction as a central driver of obesity‐associated metabolic complications [7]. Adipose tissue is not merely an energy storage organ but a complex endocrine entity, and its dysregulation can initiate systemic inflammation and insulin resistance [8]. Notably, healthy adipose tissue efficiently stores lipids, secretes beneficial adipokines, and maintains insulin sensitivity, potentially explaining the phenomenon of “metabolically healthy obesity” even under conditions of nutrient excess [9]. Therefore, understanding the mechanisms of adipose tissue dysfunction and developing strategies to enhance adipocyte health are crucial for preventing and treating obesity‐associated metabolic diseases.

The “Developmental Origins of Health and Disease” (DOHaD) concept proposes that disruptions during gestational and early postnatal development can permanently alter an individual's susceptibility to obesity and metabolic disorders when exposed to a high‐fat diet (HFD) in adulthood [10]. Mouse epididymal white adipose tissue (eWAT), a classic visceral fat depot characterized by high cellular heterogeneity and developmental plasticity, serves as an ideal model for studying this early‐life programming [11]. The postnatal initiation of eWAT differentiation marks infancy as a critical window for its metabolic programming and functional maturation [12]. During this period, lactoferrin (LF), a key bioactive component in breast milk with demonstrated immunomodulatory and growth‐promoting properties, is considered a pivotal factor for ensuring healthy development [13]. However, with less than 50% of infants globally exclusively breastfed for the first six months of life, a lot of newborns lack comprehensive LF protection during this crucial stage. According to market survey data, less than 40% of infant formula products available in the Chinese market are supplemented with LF, further limiting access for non‐breastfed infant [14]. Our previous research has confirmed that LF deficiency during lactation impairs gut barrier integrity, disrupts microbial homeostasis, and hinders neurodevelopment [15, 16, 17]. These findings underscore the urgency of investigating LF's role in early adipose tissue development.

Existing studies indicates that LF administration reduces visceral adiposity and weight gain in adult rodent models, improves insulin sensitivity and glucose homeostasis, and ameliorates hepatic steatosis [18, 19, 20]. Interestingly, while some studies report that LF promotes differentiation of human subcutaneous and visceral preadipocytes [21, 22], others demonstrate inhibitory effects on adipogenesis in 3T3‐L1 cells and rat mesenteric preadipocytes [23, 24]. Although research on LF's regulation of preadipocyte proliferation and differentiation remains limited and conclusions are inconsistent, these findings strongly suggest LF may act as a critical nutritional signal guiding adipose tissue development. The specific functions and molecular mechanisms through which LF influences postnatal adipose tissue development remain deserve further investigation.

The advancement of single‐nucleus RNA sequencing (snRNA‐seq) technology has provided a powerful tool for dissecting cellular heterogeneity and plasticity in adipose tissue. In this study, by establishing an LF‐deficient model during lactation and combining snRNA‐seq with functional experiments, we systematically analyzed the impact of postnatal LF deficiency on eWAT remodeling and transcriptional reprogramming in mice at 3, 9 weeks, and in adulthood under HFD‐induced obesity (DIO). Here, we applied snRNA‐seq technology and both in vitro and in vivo to verify LF as an enhancer of adipocyte neogenesis, a positive regulator of metabolically beneficial eWAT plasticity, and systemic nutrient homeostasis. Thus, LF supplementation during lactation may serve as an early nutritional intervention strategy for preventing obesity and related metabolic diseases.

## 2 Results

### 2.1 Lactational Deficiency of Exogenous LF Inhibits Adipocyte Hyperplasia and Induces Metabolic Reprogramming of eWAT in 3‐Week‐Old Mice

**FIGURE 1:**
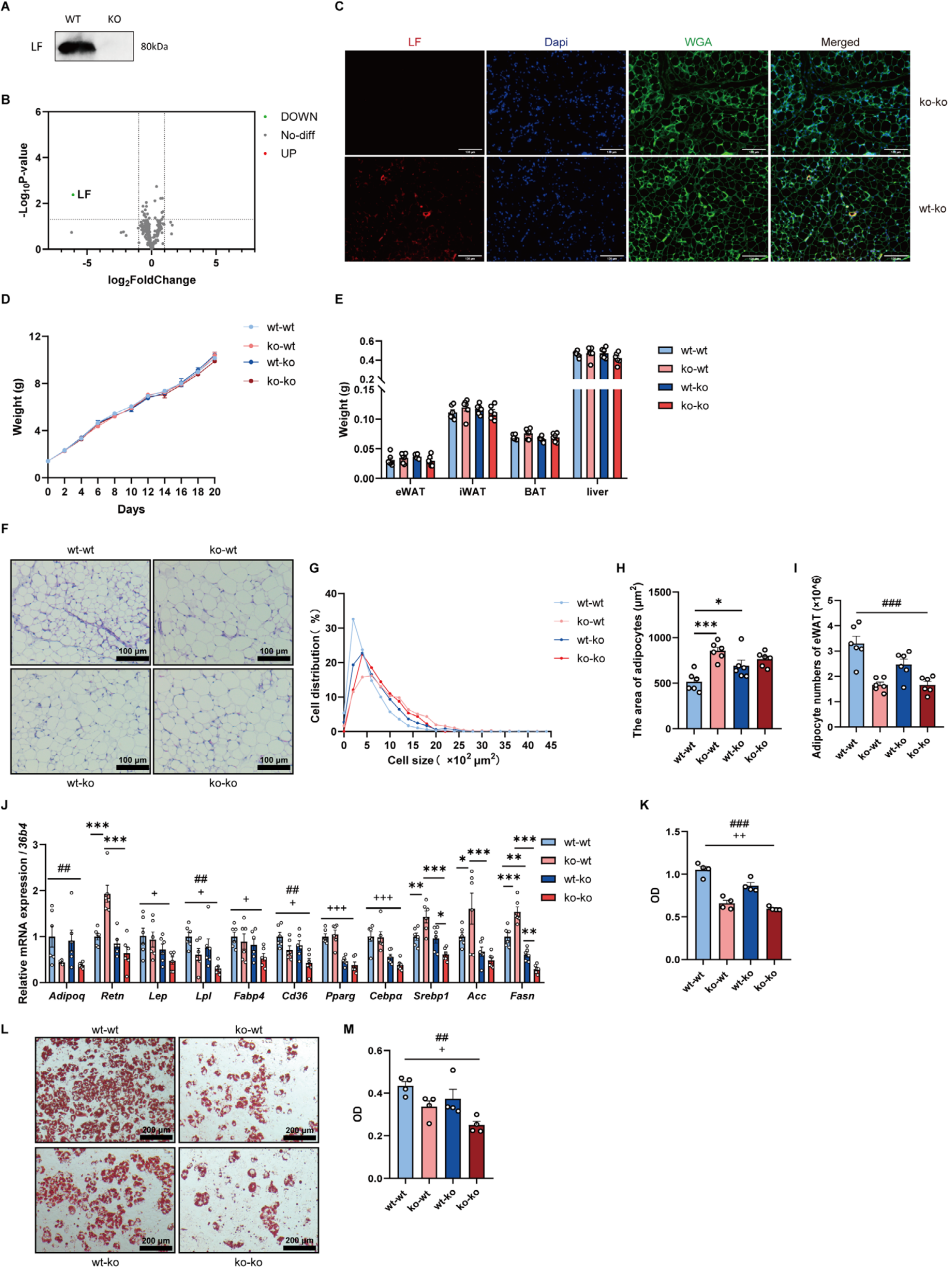
Lactational LF deficiency inhibits adipocyte hyperplasia and induces metabolic reprogramming of eWAT in 3‐week‐old mice. (A) LF protein expression in the milk of WT and KO mouse. (B) Differentially expressed proteins (|log2FC| > 1, *p*‐value < 0.05) between WT and KO mouse milk, screened from the top 200 proteins identified by mass spectrometry (MS) in WT mice. (C) Representative 100x immunofluorescence images of LF (red) location in ewat from 10‐day‐old ko‐ko mice (KO dams nursing KO pups) or wt‐ko mice (WT dams nursing KO pups). Cytomembrane were labeled by WGA (Green); Nuclei were labeled by DAPI (blue). Scale bar = 130 µm. (D) Body weight of mice during the Lactation (n = 6). (E) Organ weight of 3‐week‐old mice (n = 6). (F) Representative 200x brightfield images of H&E stained eWAT from 3‐week‐old mice. Scale bar = 100 µm. (G) Adipocyte size distribution in eWAT from 3‐week‐old mice (n = 6). (H) Adipocyte areas of eWAT from 3‐week‐old mice (n = 6). (I) Adipocyte numbers of eWAT from 3‐week‐old mice (n = 6). (J) Relative mRNA levels of target genes in eWAT from 3‐week‐old mice (n = 6). (K) SVF cells were isolated from eWAT of wt‐wt, ko‐wt, wt‐ko or ko‐ko mice and cultured in vitro for 24 h. The OD value was measured using the CCK‐8 kit (n = 4). (L) Representative 100x brightfield images of ORO‐stained SVF cells after 8 days of adipogenic differentiation from eWAT of wt‐wt, ko‐wt, wt‐ko or ko‐ko mice. Scale bar = 200 µm. (M) Absorbance values of ORO extracted from ORO‐stained SVF cells using isopropanol (n = 4). Data are mean ± SEM. Significance was calculated using two‐way ANOVA followed by Tukey's test. ^***^ *p* < 0.001, ^**^ *p* < 0.01 and ^*^ *p* < 0.05 indicate significant differences between groups; ^###^ *p* < 0.001, ^##^ *p* < 0.01 indicate significant main effects of exogenous LF; ^+++^ *p* < 0.001, ^++^ *p* < 0.01 and ^+^ *p* < 0.05 indicate significant main effects of pup genotype.

To investigate the impact of lactoferrin (LF) deficiency during lactation on adipose tissue development in mice, we generated LF knockout (KO) mice (Figure S1A). Immunostaining confirmed the absence of LF protein in the milk of KO dams, while the profiles of other milk proteins remained largely unaltered (Figure 1A; Figure S1B). Consistently, among the top 200 proteins ranked by signal intensity in WT mouse milk as determined by mass spectrometry (MS), LF was the only protein exhibiting significantly altered expression in KO mice (|log2FC| > 1, *p*‐value < 0.05) (Figure 1B). Mouse milk contained an average of 65.6 µg mL^−1^ LF, with about 7.4 µg mL^−1^ crossing into the circulation (Figure S1C–E). To establish a lactational LF deficiency model, WT newborn pups were fostered either by KO dams (ko–wt) or WT dams (wt–wt). To account for the effect of the LF gene itself, KO newborn pups were similarly divided into two groups, fostered either by KO dams (ko‐ko) or WT dams (wt‐ko).

Immunostaining showed that LF in mouse milk was transported to the epididymal white adipose tissue (eWAT) of lactating pups (Figure 1C). At the end of the lactation period (3 weeks of age), there were no significant differences in body weight or organ weight among the wt‐wt, ko‐wt, wt‐ko, and ko‐ko groups (Figure 1D,E). However, both ko‐wt and wt‐ko mice exhibited larger average adipocyte area and cell size distribution in eWAT compared with the wt‐wt control group (Figure 1F–H). Further analysis revealed that mice not receiving LF during lactation (ko‐wt and ko‐ko mice) had a significantly reduced number of eWAT adipocytes, whereas the genotype of pups had no significant effect on the number of eWAT adipocytes (Figure 1I).

The expression of adipokines serves as a key indicator of adipose tissue function. LF deficiency during lactation significantly reduced *Adipoq* gene expression in eWAT of 3‐week‐old mice, and this change was independent of pup genotype (Figure 1J). The expression level of *Retn* in ko‐wt mice was significantly higher than that in the wt‐wt control group. Additionally, LF deficiency during lactation markedly decreased the expression of the lipid uptake‐related genes *Lpl* and *Cd36*, and this effect was also independent of pup genotype (Figure 1J). However, compared with wt‐wt mice, ko‐wt mice showed increased expression of the lipogenic transcription factor *Srebp1* and its target genes that involving in de novo lipogenesis (DNL) (*Fasn*, *Acc*) were also upregulated (Figure 1J). LF gene knockout significantly reduced the expression of the adipokine *Lep*, lipid uptake‐related factors *Lpl*, *Fabp4*, and *Cd36*, as well as the adipogenic transcription factors *Pparg* and *Cebpa*, and these changes were not influenced by whether LF is consumed during lactation (Figure 1J). In wt‐ko mice, *Fasn* expression was significantly downregulated compared with wt‐wt mice. In ko‐ko mice, the expression of *Retn*, *Srebp1*, *Acc*, and *Fasn* were all significantly downregulated compared with ko‐wt mice. Furthermore, the expression of *Srebp1* and *Fasn* were significantly higher in wt‐ko mice than that in ko‐ko mice (Figure 1J).

Subsequently, primary eWAT stromal vascular fraction (SVF) cells were isolated from wt‐wt, ko‐wt, wt‐ko, and ko‐ko mice and cultured in vitro to assess proliferation and differentiation capacity. The results showed that after 24 h of culture, the absence of LF intake during lactation significantly inhibited the in vitro proliferation capacity of SVF cells (*p* = 0.000). Concurrently, LF gene deficiency in pups also inhibited SVF cell proliferation (*p* = 0.002) (Figure 1K). Specifically, CCK‐8 assay revealed that the absorbance of SVF cells in the ko‐wt group was reduced by approximately 37.3% compared with the wt‐wt group, whereas that in the wt‐ko group was reduced by 17.7% compared with the wt‐wt group. These data suggest that exogenous LF during lactation may exert a more critical influence on SVF cell proliferation than the LF genotype of pups. Moreover, Oil Red O (ORO) staining revealed that the absence of LF intake during lactation significantly inhibited the in vitro adipogenic differentiation capacity of SVF cells (*p* = 0.003), and LF gene deficiency in pups also reduced SVF cell adipogenic capacity (*p* = 0.029) (Figure 1L,M). Specifically, quantitative analysis of ORO extracted with isopropanol showed that the absorbance of SVF cells in the ko‐wt group was reduced by approximately 28.6% compared with the wt‐wt group, whereas that in the wt‐ko group was reduced by 14.1% compared with the wt‐wt group. These results suggest that exogenous LF during lactation may exert a more critical influence on the adipogenic differentiation capacity of SVF cells than the LF genotype of pups.

In conclusion, LF gene deficiency may suppress the proliferation and adipogenic differentiation of adipose‐derived stem cells or preadipocytes, whereas exogenous LF intake may be a more critical factor influencing adipose development. In vivo results support this notion, demonstrating that exogenous LF intake rescues the developmental defects caused by LF gene deficiency and exerts positive effects on both hyperplastic adipose development and adipocyte function.

### 2.2 Lactational LF Deficiency Inhibits Adipocyte Hyperplasia and Induces Metabolic Reprogramming of eWAT in Adult Mice

Given that exogenous LF intake during lactation plays a dominant role in adipose development and functional maturation, and considering the realistic reality in which some infants do not receive LF during lactation while remaining WT at the LF gene locus, this study used WT pups to investigate the long‐term effects of lactational LF deficiency on adipose development. After weaning at 3 weeks of age, wt‐wt and ko‐wt mice were maintained on a standard chow diet until 9 weeks of age.

**FIGURE 2:**
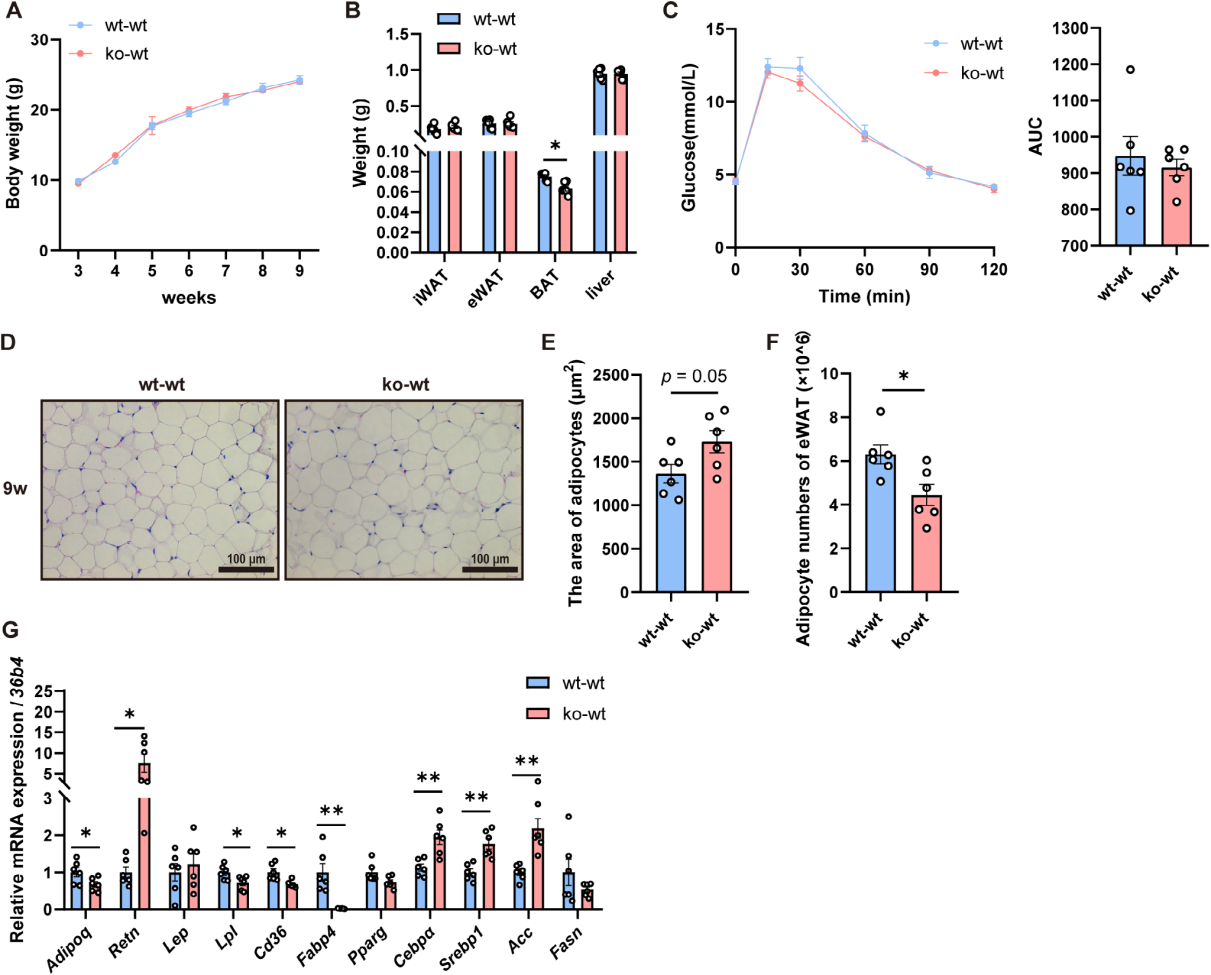
Lactational LF deficiency inhibits adipocyte hyperplasia and induces metabolic reprogramming of eWAT in 9‐week‐old mice. (A) Body weight of mice from 3 to 9 weeks of age (n = 6). (B) Organ weight of 9‐week‐old mice (n = 6). (C) Glucose tolerance tests (GTT) of 8‐week‐old mice and Area under the curve (AUC) measurements of GTT (n = 6). (D) Representative 200x brightfield images of H&E stained eWAT from 9‐week‐old mice. Scale bar = 100 µm. (E) Adipocyte areas of eWAT from 9‐week‐old mice (n = 6). (F) Adipocyte numbers of eWAT from 9‐week‐old mice (n = 6). (G) Relative mRNA levels of target genes in eWAT from 9‐week‐old mice (n = 6). Data are mean ± SEM. Significance was calculated using Student's two‐tailed unpaired *t*‐test, ^*^ *p* < 0.05 and ^**^ *p* < 0.01.

At 9 weeks, no differences in body weight, organ weight (except for BAT), or glucose tolerance tests (GTT) were observed (Figure 2A–C). The weight of BAT was decreased in ko‐wt mice. Notably, the average area of adipocytes was larger, and the number of adipocytes was significantly reduced in the eWAT of 9‐week‐old ko‐wt mice (Figure 2D–F), consistent with the phenotype observed at 3‐week‐old. In addition, *Adipoq* remained downregulated, and *Retn* upregulated in 9‐week‐old ko–wt eWAT (Figure 2G). Lipid uptake genes (*Lpl*, *Cd36*, *Fabp4*) were suppressed, while DNL‐related genes (*Srebp1, Acc*) were elevated. These findings indicate that lactational LF deficiency exerts persistent effects on adipose tissue development from lactation through adulthood.

### 2.3 Lactation LF Deficiency Drives Adipocyte Hypertrophy and Metabolic Dysfunction in Obese Mice

**FIGURE 3:**
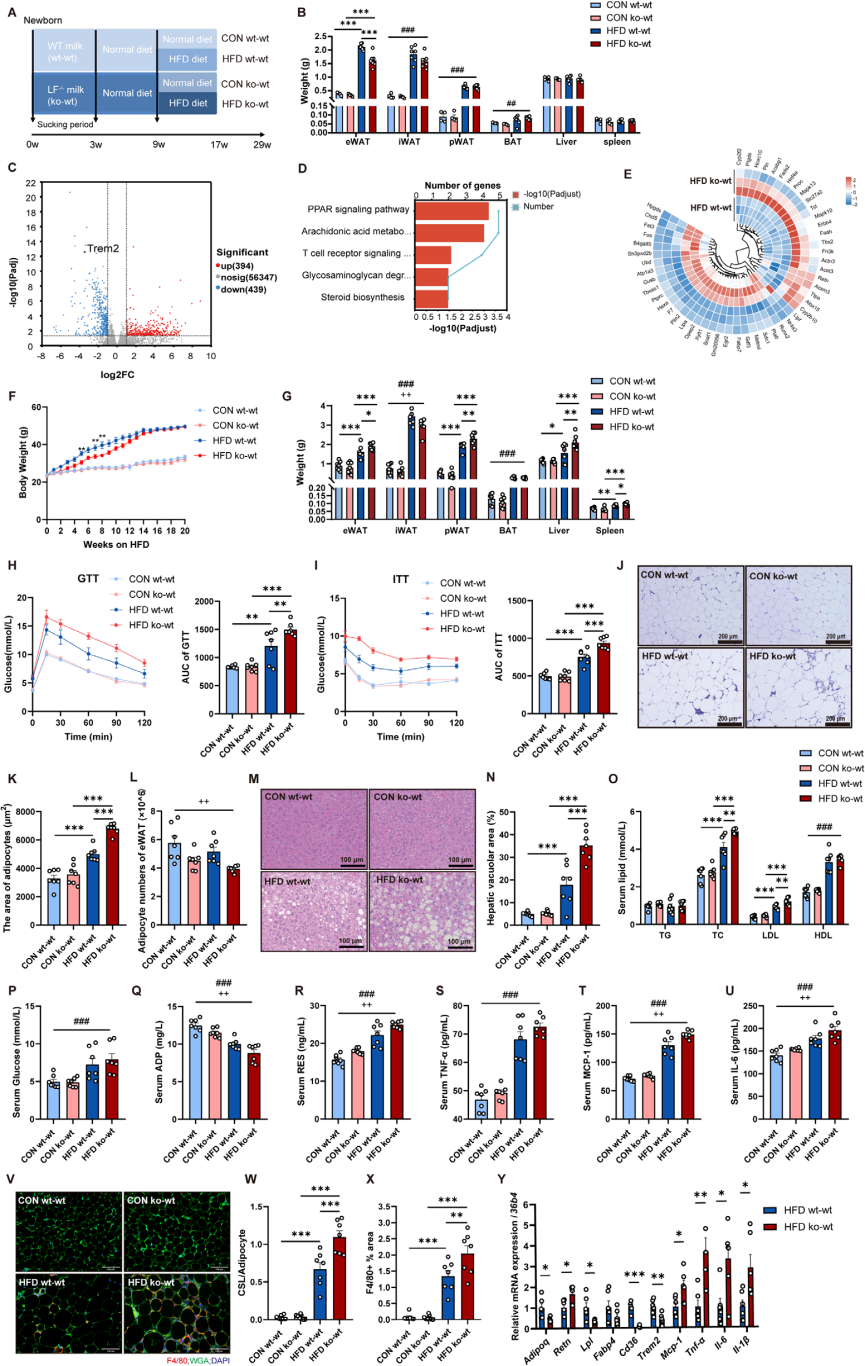
Lactation LF deficiency leads to pathological hypertrophy expansion of eWAT adipocyte and exacerbates metabolic dysfunction. (A) Schematic diagram of the establishment of the lactational LF deficiency model and obesity model. (B) Tissue weights of mice after 8 weeks of HFD (n = 4/CON group; n = 7/HFD group). (C) Differential expression genes (DEGs) between HFD wt‐wt and HFD ko‐wt mice after 8 weeks of HFD (|log2FC| > 1, adjusted *p*‐value (padj) < 0.05) (n = 3). (D) KEGG enrichment analysis of fat‐related DEGs between HFD wt‐wt and HFD ko‐wt mice after 8 weeks of HFD (n = 3). (E) The clustering heatmap of fat‐related DEGs between HFD wt‐wt and HFD ko‐wt mice after 8 weeks of HFD (n = 3). (F) Weekly body weights of mice during the 20 weeks HFD feeding period (n = 7). (G) Organ weight of mice after 20 weeks HFD feeding (n = 7). (H) GTT after 18 weeks of HFD feeding and AUC measurements of GTT (n = 7). (I) Insulin tolerance tests (ITT) after 19 weeks of HFD feeding and AUC measurements of ITT (n = 7). (J) Representative 100x brightfield images of H&E‐stained eWAT from 20 weeks of HFD feeding mice. Scale bar = 200 µm. (K) Adipocyte areas of eWAT from 20 weeks of HFD feeding mice (n = 7). (L) Adipocyte numbers of eWAT from 20 weeks of HFD feeding mice (n = 7). (M) Representative 400x brightfield images of H&E stained liver from 20 weeks of HFD feeding mice. Scale bar = 100 µm. (N) Quantification of the percentage of vacuolar area, at least 6 fields of view counted per sample (n = 7). (O) Serum TG, TC, LDL, HDL levels of mice after 20 weeks of HFD feeding (n = 7). (P‐U) Serum glucose, adiponectin (ADP), resistin (RES), TNF‐α, MCP‐1, IL‐6 levels after 20 weeks of HFD feeding (n = 7). (V) Representative 100x immunofluorescence images of F4/80 (red) expression in ewat from 20 weeks of HFD feeding mice. Cytomembrane were labeled by WGA (Green); Nuclei were labeled by DAPI (blue). Scale bar = 130 µm. (W‐X) Quantification of the fraction of crown‐like structures (CSL) (W) and the percentage of F4/80‐positive area (X), at least 6 fields of view counted per sample (n = 7). (Y) Relative mRNA levels of target genes in eWAT from 20 weeks of HFD feeding mice (n = 6). Data are mean ± SEM. Significance for figure Y was calculated using Student's two‐tailed unpaired *t*‐test, ^***^ *p* < 0.001, ^**^ *p* < 0.01, and ^*^ *p* < 0.05. Significance for remain Figures was calculated using two‐way ANOVA followed by Tukey's test. ^***^ *p* < 0.001, ^**^ *p* < 0.01, and ^*^ *p* < 0.05 indicate significant differences between groups; ^###^ *p* < 0.001, ^##^ *p* < 0.01 indicate significant main effects of diet; ^++^ *p* < 0.01 indicate a significant main effect of lactational exogenous LF. RNA‐seq data visualizations were generated using Majorbio.com.

To investigate whether the impact of lactational LF deficiency on adipose tissue development leads to altered susceptibility to DIO in adulthood, we subjected 9‐week‐old wt‐wt and ko‐wt mice to a HFD intervention to establish a DIO model, with a standard chow diet as control (CON). This resulted in four experimental groups: CON wt‐wt, CON ko‐wt, HFD wt‐wt, and HFD ko‐wt (Figure 3A).

After 8 weeks of HFD feeding, HFD significantly increased body weight and the weights of inguinal WAT (iWAT), eWAT, perirenal WAT (pWAT), and brown adipose tissue (BAT) compared with the CON group (Figure 3B; Figure S2A). However, HFD ko–wt mice exhibited lower body weight and eWAT mass than HFD wt–wt mice, with no differences in food intake between wt‐wt and ko‐wt mice under either diet (Figure S2B). GTT revealed that HFD significantly impaired glucose tolerance, and lactational LF deficiency further exacerbated this impairment (Figure S2C). Serum lipid analysis showed that HFD significantly increased total cholesterol (TC), high‐density lipoprotein (HDL), and low‐density lipoprotein cholesterol (LDL) levels. Notably, lactational LF deficiency not only significantly elevated serum LDL levels, but also markedly increased very‐low‐density lipoprotein (vLDL) levels under HFD conditions (Figure S2D).

To explore the underlying mechanisms, we performed RNA sequencing (RNA‐seq) analysis on eWAT from obese mice after 8 weeks of HFD. We identified 833 differentially expressed genes (DEGs) between the HFD ko‐wt and HFD wt‐wt mice, with 394 genes upregulated and 439 genes downregulated (|log2FC| > 1, adjusted *p*‐value (padj) < 0.05) (Figure 3C). Among the top 10 significant DEGs, *Trem2* expression was significantly reduced in the HFD ko‐wt group. *Trem2* is positively correlated with insulin sensitivity, and its deficiency promotes adipocyte hypertrophy and apoptosis [25]. Further analysis showed that obesity‐related DEGs were significantly enriched in the PPAR signaling pathway (Figure 3D). Within this pathway, lipid synthesis‐related genes (*Fads2*, *Acsbg1*) were upregulated, while those involved in lipid uptake (*Fabp7*) and lipolysis (*Lipa*) were downregulated (Figure 3E). Additionally, the expression of *Plin2* (associated with adipocyte neogenesis) and *Fos* (involved in adipogenesis) were both markedly reduced, whereas the expression of *Retn* was significantly elevated in HFD ko‐wt mice.

To assess the effects of lactational LF deficiency on long‐term HFD feeding in adulthood, mice were subjected to HFD for 20 weeks. After 20 weeks of HFD feeding, body weight and the mass of various fat depots and organs increased (Figure 3F,G; Figure S2E,F). Compared to HFD wt‐wt mice, HFD ko‐wt mice still exhibited lower body weight at weeks 7 and 8 of the HFD. ko‐wt mice had less iWAT compared with wt‐wt mice, but HFD ko‐wt mice had larger eWAT, pWAT, liver, and spleen depots relative to HFD wt‐wt controls, although no differences in body weight or food intake were observed between ko‐wt and wt‐wt mice under either HFD or CON condition (Figure S2G). GTT and insulin tolerance tests (ITT) revealed that lactational LF deficiency exacerbated HFD‐induced impairments in glucose homeostasis and insulin sensitivity (Figure 3H,I).

Histological analysis showed that LF deficiency exhibited a larger average adipocyte area in eWAT of obese mice after 20 weeks of HFD, with the distribution curve of adipocyte area shifted to the right (Figure 3J,K; Figure S2H). The number of adipocytes decreased significantly, a change unaffected by dietary conditions (Figure 3L). Additionally, LF deficiency aggravated hepatic steatosis, with HFD ko–wt mice exhibiting more severe lipid droplet accumulation in the liver (Figure 3M,N).

After 20 weeks of HFD, serum lipid and glucose levels were elevated (Figure 3O,P). The HFD‐induced increases in serum TC and LDL were significantly increased in HFD ko‐wt mice compared with HFD wt‐wt controls. In addition, HFD significantly decreased serum Adiponectin (ADP) levels and increased Resistin (RES) levels (Figure 3Q,R). Importantly, lactational LF deficiency led to the same disorder of adipokine secretion, and this phenotypic change was not affected by dietary conditions. Moreover, HFD significantly increased the levels of serum TNF‐α, MCP‐1, and IL‐6, with LF deficiency further elevating MCP‐1 and IL‐6 levels independent of diet (Figure 3S–U).

Immune cell infiltration into adipose tissue and subsequent inflammatory responses are commonly observed in DIO. After 20 weeks of HFD, eWAT from HFD ko‐wt mice contained more crown‐like structures (CLS) and F4/80^+^ inflammatory cells than HFD wt‐wt mice, indicating a more severe inflammatory state and an unhealth pathological phenotype (Figure 3V–X; Figure S2I).

Next, we detected the gene expression levels in the eWAT of obese mice after 20 weeks of HFD. Consistent with the expression trends observed in 3‐week‐old and 9‐week‐old mice, lactational LF deficiency significantly reduced *Adipoq*, *Lpl* and *Cd36* expression, while increased *Retn* expression (Figure 3Y). Additionally, the expression of *Trem2* was decreased in HFD ko‐wt mice. Moreover, lactational LF deficiency significantly increased the expression of *Mcp‐1*, *Tnf‐α*, *Il‐6*, and *Il‐1β* (Figure 3Y).

### 2.4 Lactational LF Deficiency Reduces eWAT Plasticity by Disrupting Developmental Cellular Composition and Metabolic Programs

**FIGURE 4:**
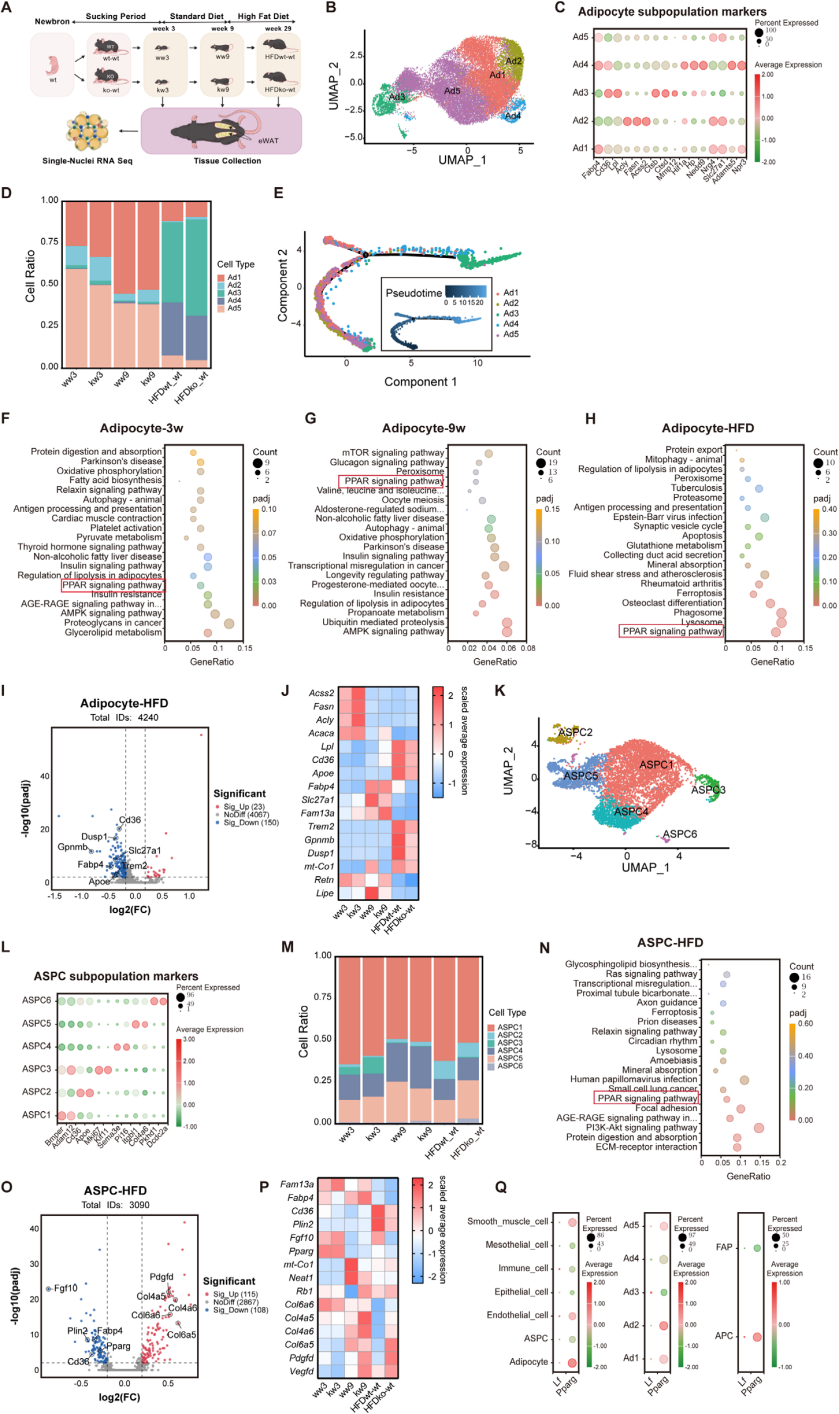
Lactational LF deficiency alters adipocyte and ASPC subpopulation composition and impairs their metabolic function. (A) Outline of the workflow. We performed snRNA‐seq on nuclei from pools of ewat from 3‐week‐old wt‐wt and ko‐wt (ww3 and kw3), 9‐week‐old wt‐wt and ko‐wt (ww9 and kw9), 20 weeks HFD wt‐wt and ko‐wt (HFDwt‐wt and HFDko‐wt) mice (n = 1). Created with BioGDP.com [32]. (B) UMAP of adipocyte subpopulations. (C) Dotplot showing scaled average expression of adipocyte subpopulation marker genes: Ad1 (*Fabp4*, *Cd36*, *Lpl*), Ad2 (*Acly*, *Fasn*, *Acss2*), Ad3 (*Ctsb*, *Ctsd*, *Mmp12*), Ad4 (*Hif1a*, *Hp*, *Nedd9*), and Ad5 (high expression of *Nrg4* and *Slc27a1*, low expression of *Adamts5* and *Npr3*). (D) The average fraction (relative to the total number of adipocyte nuclei) of each subpopulation. (E) Pseudo‐time cell trajectory of adipocyte subpopulations. (F‐H) KEGG enrichment analysis of DEGs (|log2FC| > 0.18, padj < 0.01) in adipocytes between ko‐wt group and wt‐wt group of 3‐week‐old mice (F), 9‐week‐old mice (G), 20 weeks HFD feeding mice (H). (I) Volcano Plot showing DEGs of adipocyte subpopulation between ko‐wt group and wt‐wt group of 20 weeks HFD feeding mice. (J) Heatmap showing DEGs in adipocytes between ko‐wt group and wt‐wt group. (K) UMAP of ASPC subpopulations. (L) Dotplot showing scaled average expression of ASPC subpopulation marker genes: ASPC1 (*Bmper*, *Adam12*), ASPC2 (*Cd36*, *Apoe*), ASPC3 (*Mki67*, *Kif11*), ASPC4 (*Sema3e*, *Pi16*), ASPC5 (*Itgbl1*, *Col4a6*), and ASPC6 (*Pkhd1*, *Dcdc2a*). (M) The average fraction (relative to the total number of ASPC nuclei) of each subpopulation. (N) KEGG enrichment analysis of DEGs (|log2FC| > 0.2, padj < 0.01) in ASPC between ko‐wt group and wt‐wt group of 20 weeks HFD feeding mice. (O) Volcano Plot showing DEGs of ASPC subpopulation between ko‐wt group and wt‐wt group of 20 weeks HFD feeding mice. (P) Heatmap showing DEGs in ASPC between ko‐wt group and wt‐wt group. (Q) The expression of the *Lf* and *Pparg* in all cell clusters, adipocyte subpopulations and ASPC subpopulations. snRNA‐seq data visualizations were generated using NovoMagic (magic.novogene.com).

To construct a comprehensive single‐nucleus atlas of eWAT and investigate how lactational LF deficiency affects its cellular plasticity, we performed snRNA‐seq on eWAT from mice under three conditions: lactation (3 weeks postpartum), adulthood (9 weeks), and obesity (20‐week HFD from 9 weeks of age) (Figure 4A). Each stage included mice lacking lactational LF and controls. After quality control, we integrated 6 datasets, including 142660 nuclei. Based on DEGs, we annotated the following cell types: adipocyte, adipose stem and progenitor cell (ASPC), immune cell, endothelial cell, mesothelial cell, epithelial cell (EC), smooth muscle cell, spermatozoa, and epididymal cell (Figure S3A,B). Both spermatoza and epididymal cells were found to contain nuclei originating from the epididymis. Since the epididymis is anatomically connected to eWAT in the head and tail, the observed admixture of eididymal nuclei is consistent with expectations [11, 26]. However, given the low number of epididymal nuclei detected in our datasets, we conclude that contamination from these epididymal cell clusters is negligible.

Comparative analysis of eWAT composition showed an increase in immune cell proportion during development, particularly under obese conditions (Figure S3C). While adipocyte abundance decreased in obese mice, likely due to immune infiltration.

Re‐clustering of adipocytes (n = 22360) revealed five subpopulations (Figure 4B,C). Ad2 showed high expression of genes associated with lipid biosynthesis, indicating that they are lipogenic adipocytes with a high capacity for DNL. The nuclei of Ad1, Ad3, Ad4, and Ad5 showed high expression of genes associated with lipid uptake and transport, indicating a lipid‐scavenging role that likely acquires lipids primarily through uptake rather than DNL. Ad3 also showed inflammatory signatures, Ad4 exhibited high expression of genes involved in stress response, and Ad5 was distinguished by high expression of genes involved in promoting thermos genesis and low expression of genes involved in inhibiting thermogenesis.

Analysis of adipocyte composition revealed that compared to 3‐week‐old mice, 9‐week‐old mice displayed an increased proportion of Ad1, with decreased Ad2 and Ad5 (Figure 4D; Figure S3D). In obesity, the proportions of Ad2 and Ad5 were further reduced, and a reduction in Ad1 was also observed. Conversely, the proportions of Ad3 and Ad4 were increased, indicating a pro‐inflammatory shift. Pseudo‐time cell trajectory analysis demonstrated that Ad3 was predominantly enriched in the late phase of the developmental trajectory, whereas Ad2 and Ad5 was primarily concentrated in the early stage (Figure 4E; Figure S3E). Importantly, LF deficiency increased Ad3 by 20% in obese mice (Figure 4D; Figure S3D). Moreover, in both obese mice and 3‐week‐old mice, lactational LF deficiency reduced the proportion of Ad5 by 36% and 16%, respectively.

In addition to assessing the proportional changes of different adipocyte subpopulation, we further investigated whether the absence of LF intake during lactation affects the transcriptional program of adipocytes. We detected DEGs in adipocyte subpopulations from 3‐week‐old, 9‐week‐old, and obese mice, respectively. KEGG pathway enrichment analysis demonstrated significant enrichment of these DEGs in the PPAR signaling pathway (Figure 4F–H). In mouse with lactational LF deficiency, the expression of multiple genes involved in lipid transport and uptake was suppressed in adipocyte subpopulations. Specifically, *Lpl* was downregulated in 3‐week‐old mice; *Slc27a1* was reduced in 9‐week‐old mice; *Cd36*, *Fabp4*, and *Slc27a1* were significantly inhibited in obese mice (Figure 4I,J; Figure S3F). These alterations suggest that lactational LF deficiency may impair lipid clearance in eWAT adipocytes. Lactational LF deficiency also promoted the upregulation of genes related to DNL in both 3‐week‐old and 9‐week‐old mice. Specifically, *Acly*, *Acss2*, and *Fasn* were elevated in 3‐week‐old mice, and *Acaca* were upregulated in 9‐week‐old mice (Figure 4J; Figure S3F). *Fam13a*, a gene associated with increased adipocyte size and apoptosis [27], was upregulated in both 3‐week‐old and 9‐week‐old mice. *mt‐Co1* as a gene linked to mitochondrial dysfunction, was also upregulated at both ages (Figure 4J; Figure S3F). In 9‐week‐old mice, lactational LF deficiency resulted in decreased expression of the lipolysis‐related gene *Lipe* and increased expression of the insulin resistance‐related gene *Retn*. In obese mice, lactational LF deficiency exhibited downregulation of *Trem2*. Moreover, it also resulted in decreased expression of the anti‐inflammatory genes *Gpnmb* and *Dusp1* (Figure 4I,J). These genes are closely associated with macrophage‐driven inflammation [28, 29].

Re‐clustered ASPCs (n = 7288) formed six subpopulations (Figure 4K,L). ASPC1, ASPC2, ASPC3, and ASPC6 exhibited high expression levels of adipogenic regulators, indicating that they are adipogenic progenitor cells (APCs). Among these, ASPC2 showed the highest expression of genes associated with fatty acid uptake, suggesting its role of lipid‐absorbing adipogenic progenitor. ASPC3 expressed the highest levels of proliferation‐related genes, supporting its classification as a proliferative adipogenic progenitor. ASPC6 was characterized by elevated expression of epithelial marker genes, so identified as an epithelial‐like adipogenic progenitor. In addition, ASPC4 highly expressed pro‐inflammatory factors, while ASPC5 showed enriched expression of fibrosis‐related genes. Therefore, ASPC4 and ASPC5 were collectively defined as fibro‐inflammatory adipogenic progenitor cells (FAPs), distinct from APC.

Interestingly, LF deficiency reduced APC proportion (especially ASPC1 and ASPC2) by 14% and increased FAPs by 40% in obese mice (Figure 4M; Figure S3G). DEGs in ASPCs between HFD wt‐wt and HFD ko‐wt mice revealed significant enrichment in the PPAR signaling pathway (Figure 4N). Notably, in the HFD ko‐wt group, genes associated with fatty acid uptake (*Cd36*, *Fabp4*), adipocyte development (*Pparg*, *Fgf10*), and a nascent adipocyte marker (*Plin2*) were downregulated, indicating impaired adipogenesis (Figure 4O,P). This phenomenon was further corroborated in the 3‐week‐old mice, where *Fabp4* also showed significant downregulation in ko‐wt group (Figure 4P; Figure S3H). Furthermore, in ASPCs from obese mice, the HFD ko‐wt group exhibited significantly upregulation of multiple collagen genes (*Col6a5*, *Col4a6*, and *Col6a6*) (Figure 4O,P). Inflammatory microenvironment induces similar extracellular matrix proteins in adipose precursor cells [30]. Additionally, upregulation of pro‐inflammatory cytokine (*Pdgfd*) was observed in the HFD ko‐wt group, suggesting fibro‐inflammatory reprogramming. Importantly, this phenomenon was consistently observed across both 3‐week‐old and 9‐week‐old mice. In 3‐week‐old mice, pro‐apoptotic genes (*Fam13a*) increased. In 9‐week‐old mice, *Fam13a*, *Pdgfd, Vegfd* were also increased (Figure 4P; Figure S3H). Furthermore, lactational LF deficiency led to upregulation of *Rb1*, which inhibits cell proliferation.

snRNA‐seq analysis revealed that, among all cell subpopulations in eWAT, *Lf* was mainly expressed in immune cells, though *Lf*‐expressing cells accounted for less than 0.5% of each subpopulation (Figure 4Q). Within the adipocyte subpopulations, *Lf* expression was primarily detected in the Ad3 subpopulation, yet the proportion of *Lf*‐expressing cells remained below 0.5% across all adipocyte subpopulations. In the ASPC subpopulations, *Lf* showed relatively high expression in the APC subpopulation, but the proportion of *Lf*‐expressing cells was less than 0.2% in both APC and FAP subpopulations. In contrast, *Pparg*‐expressing cells were abundant across all subpopulations, with *Pparg* positivity reaching as high as 86% in adipocytes, where it was also expressed at high levels (Figure 4Q).

LF deficiency also increased EC abundance in obese and 3‐week‐old mice (Figure S3C). Re‐clustered EC nuclei (n = 6689) formed three subsets: normal (EC1), inflammatory (EC2), and stressed (EC3) (Figure S3I,J). Compared to the HFD wt‐wt group, the relative abundance of the EC2 and EC3 in the HFD ko‐wt group were increased (Figure S3K). DEGs were enriched in the oxidative phosphorylation pathway (Figure S3L), with inflammatory‐related genes (*Spp1*, *Pdgfd*, *Pde10a*) upregulated (Figure S3M,N).

Re‐clustered immune cells (n = 21464) included B cells, dendritic cells, macrophages, mast cells, natural killer (NK) cells, and T cells (Figure S3O,P). Immune cell subpopulations increased during development, particularly under obese conditions (Figure S3Q). Among immune cells, macrophages are the most abundant in eWAT (n = 13857). We further identified macrophages as M1, M2, lipid‐associated macrophages (LAM), proliferative LAM (P‐LAM), and regulatory macrophages (RM) (Figure S3R,S). M1 and M2 represent the classic polarization of macrophages [31]. LAM participates in the clearance of dead adipocytes and lipids [31]. RM secretes ligands that improving insulin sensitivity of adipocyte [11]. The results showed that obesity increased LAM but decreased RM proportion (Figure S3T). Notably, lactational LF deficiency reduced RM proportion by 44% in 3‐week‐old mice and 57% in obese mice. In obese mice, lactational LF deficiency downregulated genes involved in lipid uptake (*Cd36*, *Fabp4*), lipolysis (*Lipa*), and anti‐inflammatory responses (*Gpnmb*, *Dusp1*), while upregulating pro‐inflammatory genes (*Aim2*) (Figure S3U,V). Similar expression patterns occurred in early developmental stages. In 3‐week‐old mice, LF deficiency similarly reduced lipid uptake‐related genes (*Lpl, Fabp4*). Additionally, LF deficiency decreased the expression of *Apoe*, a key gene involved in adipose tissue cholesterol homeostasis, in both 3‐week‐old and obese mice. The expression of *mt‐Co1* was reduced in both 3‐week‐old and 9‐week‐old mice. In 9‐week‐old mice, increased expression of pro‐inflammatory factors (*Nfkb1*, *Itga4*) was also observed (Figure S3U,V).

### 2.5 Recombinant Human LF Promotes Proliferation and Adipogenic Differentiation of Adipose Stem Cells and Preadipocytes In Vitro

**FIGURE 5:**
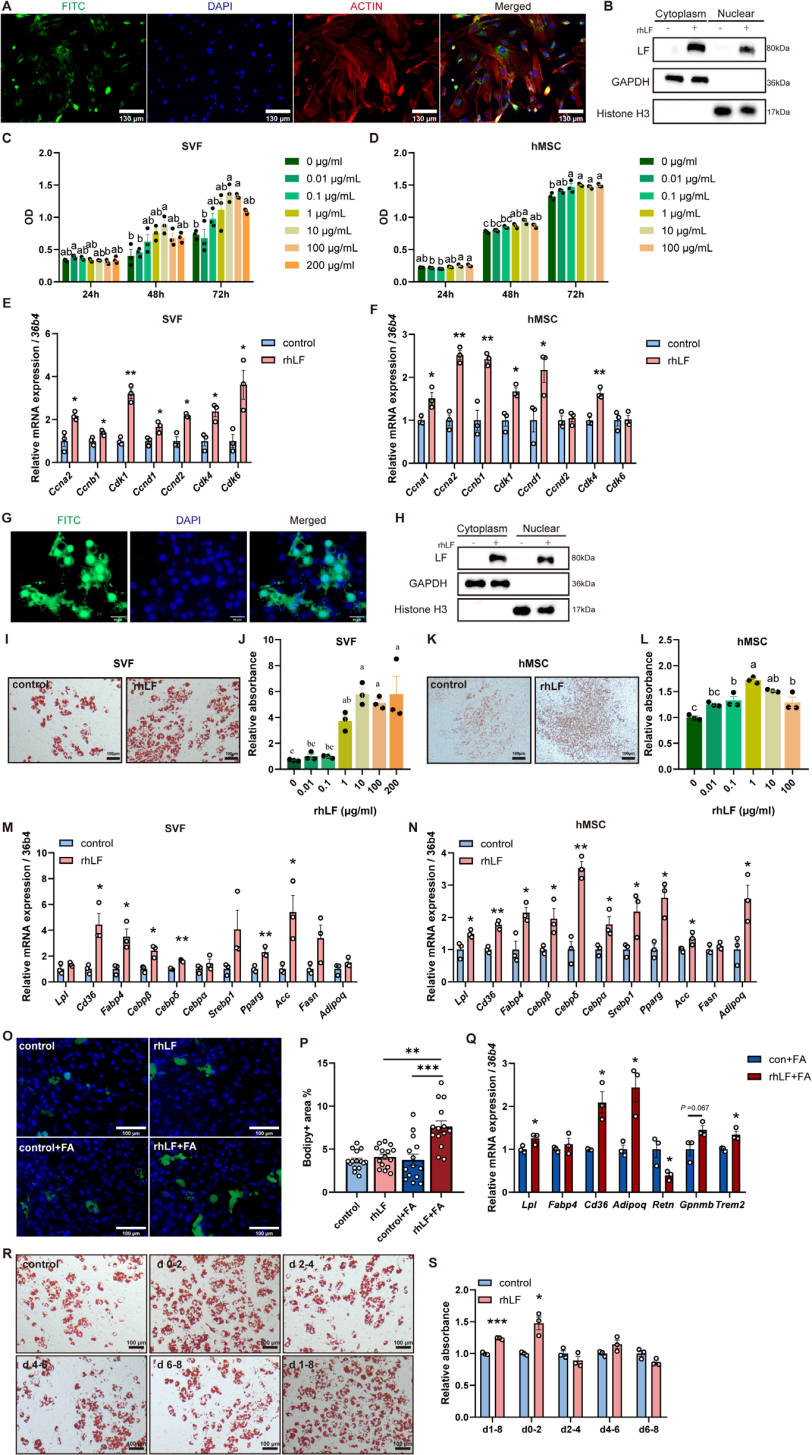
rhLF treatment promotes the proliferation and adipogenic differentiation of SVF cells and hMSCs in vitro. (A) Representative 100x immunofluorescence images of rhLF (green) location in SVF cells after 48 h of FITC‐rhLF treatment. ACTIN were labeled by Phalloidin (Red); Nuclei were labeled by DAPI (blue). Scale bar = 130 µm. (B) Immunoblotting of rhLF distribution in SVF cells after 48 h of rhLF treatment. (C, D) SVF cells or hMSCs were treated with various concentrations of rhLF for 24, 48, and 72 h, then added CCK‐8 to determine the absorbance (n = 3). (E, F) Relative mRNA levels of target genes in SVF cells or hMSCs treated with 10 µg mL^−1^ rhLF (n = 3). (G) Representative 400x immunofluorescence images of rhLF (green) location in SVF cells after 8 days of adipogenic differentiation and FITC‐rhLF treatment. Nuclei were labeled by DAPI (blue). Scale bar = 50 µm. (H) Immunoblotting of rhLF distribution in SVF cells after 8 days of adipogenic differentiation and rhLF treatment. (I) Representative 100x brightfield images of ORO‐stained SVF cells after 8 days of adipogenic differentiation under treatment with 10 µg mL^−1^ rhLF. Scale bar = 100 µm. (J) Relative absorbance values of ORO extracted from ORO‐stained SVF cells using isopropanol after 8 days of adipogenic differentiation with various concentrations of rhLF (n = 3). (K) Representative 100x brightfield images of ORO‐stained hMSCs after 8 days of adipogenic differentiation under treatment with 1 µg mL^−1^ rhLF. Scale bar = 100 µm. (L) Relative absorbance values of ORO extracted from ORO‐stained hMSCs using isopropanol after 8 days of adipogenic differentiation with various concentrations of rhLF (n = 3). (M) Relative mRNA levels of target genes in SVF cells after 8 days of adipogenic differentiation under treatment with 10 µg mL^−1^ rhLF (n = 3). (N) Relative mRNA levels of target genes in hMSCs after 8 days of adipogenic differentiation under treatment with 1 µg mL^−1^ rhLF (n = 3). (O) Representative 400x immunofluorescence images of BODIPY‐labeled lauric acid (green) in SVF cells. The cells were first treated with 10 µg mL^−1^ rhLF followed by 8 days of adipogenic differentiation, and then treated with or without 1mm fatty acid for 24 h. BODIPY‐labeled lauric acid was used to assess lipid uptake capacity. Nuclei were labeled by DAPI (blue). Scale bar = 100 µm. (P) The percentage of BODIPY‐positive area per field of view, with n = 15 fields of view counted per group. (Q) Relative mRNA levels of target genes in SVF cells after 8 days of adipogenic differentiation under treatment with 10 µg mL^−1^ rhLF, and then treated with 1 mm fatty acid for 24 h (n = 3). (R) Representative 100x brightfield images of ORO‐stained SVF cells after 8 days of adipogenic differentiation. Cells in each group were treated with 10 µg mL^−1^ rhLF during distinct periods of adipogenic differentiation: 0–2 days, 2–4 days, 4–6 days, 6–8 days, and 1–8 days, respectively (n = 3). Scale bar = 100 µm. (S) Relative absorbance values of ORO extracted from ORO‐stained SVF cells using isopropanol were measured (n = 3). The group treated with 0 µg mL^−1^ rhLF served as the control. Data are mean ± SEM. Significance for Figure 5C,D,J,L was calculated using one‐way ANOVA followed by Tukey's test; Different letters indicate statistically significant differences. Significance for Figure 5E,F,M,N,Q,S was calculated using Student's two‐tailed unpaired *t*‐test, ^***^ *p* < 0.001, ^**^ *p* < 0.01, and ^*^ *p* < 0.05. Significance for Figure 5P was calculated using two‐way ANOVA followed by Tukey's test; ^**^ *p* < 0.01 and ^***^ *p* < 0.001.

To further investigate the regulation of LF in adipocyte development, SVF cells was isolated from mouse eWAT and treated with recombinant human LF (rhLF). Immunoblotting and immunostaining confirmed the efficient uptake of exogenous rhLF into SVF cells, with localization in both the cytoplasm and nucleus (Figure 5A,B; Figure S4A). CCK8 assay results showed that treatment with 10–100 µg mL^−1^ rhLF for 72 h significantly enhanced the viability of SVF cells, while 0.1–100 µg mL^−1^ rhLF for 72 h significantly enhanced the viability of human adipose‐derived mesenchymal stem cells (hMSCs) (Figure 5C,D). At 48 h of treatment, 10 µg mL^−1^ rhLF was the most effective concentration for promoting the viability of both cell types. This finding was supported by the upregulation of key cell cycle regulators. In SVF cells, the expression of cyclins (*Ccna2*, *Ccnb1*, *Ccnd1*, *Ccnd2*) and cyclin‐dependent kinases (*Cdk1*, *Cdk4*, *Cdk6*) was significantly increased (Figure 5E). Similarly, in hMSCs, the expression of *Ccna1*, *Ccna2*, *Ccnb1*, *Ccnd1*, *Cdk1*, and *Cdk4* was also significantly upregulated (Figure 5F). These results indicate that rhLF promotes the proliferation of both SVF cells and hMSCs.

Similarly, rhLF can enter both the cytoplasm and nucleus of SVF cells after 8 days of adipogenic differentiation (Figure 5G,H; Figure S4B). Treatment with 1–200 µg mL^−1^ rhLF enhanced the adipogenic differentiation capacity of SVF cells, as evidenced by increased Oil Red O (ORO) staining, especially at 10 ug mL^−1^ (Figure 5I,J). Treatment with 0.1–100 µg mL^−1^ rhLF enhanced the adipogenic differentiation capacity of hMSCs, especially at 1 ug mL^−1^ (Figure 5K,L). In SVF cells, 10 µg mL^−1^ rhLF treatment increased the expression of adipogenic genes involved in lipid uptake (*Cd36*, *Fabp4*), adipogenic transcription factors (*Cebp*β, *Cebp*δ, *Pparg*), and the DNL gene (*Acc*) (Figure 5M). In hMSCs, 1 µg mL^−1^ rhLF treatment increased the expression of genes involved in lipid uptake (*Lpl*, *Cd36*, *Fabp4*), adipogenic transcription factors (*Cebp*β, *Cebp*δ, *Cebp*α, *Pparg*), DNL genes (*Srebp1*, *Acc*), and the mature adipocyte marker *Adipoq* (Figure 5N).

We next assessed whether rhLF influences mature adipocyte function. Following differentiation, rhLF‐treated cells exhibited enhanced fatty acid uptake under lipid‐overloaded conditions (Figure 5O,P), along with increased expression of lipid transport genes (*Lpl*, *Cd36*) (Figure 5Q). rhLF treatment also improved metabolic and inflammatory profiles, elevating *Gpnmb*, *Trem2*, and *Adipoq* expression, while suppressing *Retn*, suggesting a shift toward a healthier adipocyte phenotype.

To investigate the critical window of rhLF action, we administered it during distinct differentiation phases: early (days 0–2), mid (days 2–4), mid‐late (days 4–6), and terminal (days 6–8). Exogenous rhLF into both the cytosol and nucleus of SVF cells throughout differentiation (Figure S4C,D). However, rhLF exposure restricted specifically to the early phase was sufficient to significantly enhance adipogenesis, demonstrated by more staining with ORO (Figure 5R,S).

### 2.6 rhLF Promotes SVF Cell Proliferation by Enhancing CSK Degradation to Inhibit the CSK‐SRC Pathway In Vitro

**FIGURE 6:**
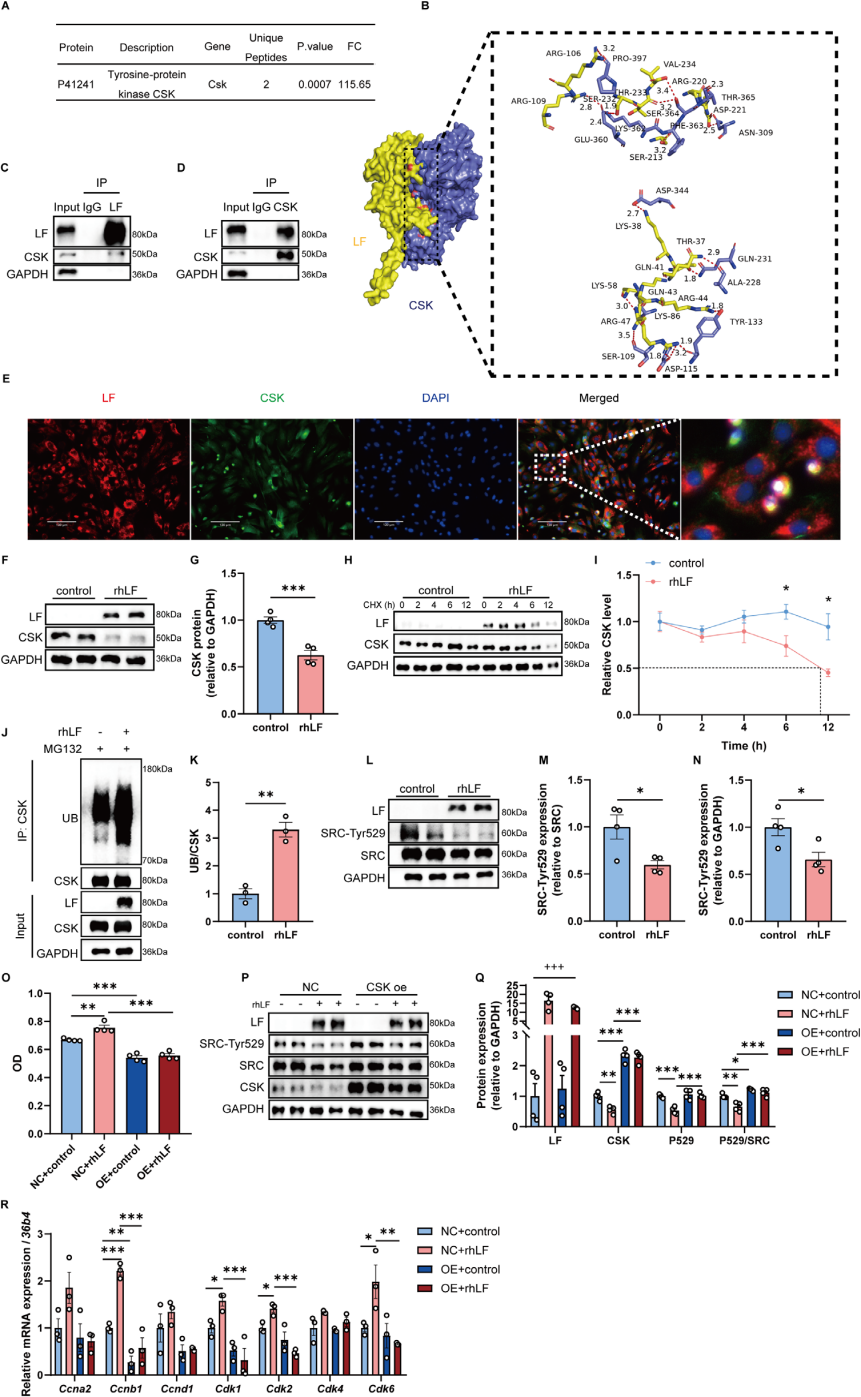
rhLF promotes SVF cells proliferation via the CSK‐SRC pathway. (A) The CSK protein in the IP product of rhLF was discovered through IP/MS analysis. (B) Molecular docking analysis of CSK (P41241) and rhLF (Q8IX02). (C, D) Co‐IP analysis of the interaction between CSK and rhLF in SVF cells was performed with anti‐CSK or anti‐LF primary antibodies (n = 3). (E) Representative 100x immunofluorescence images of rhLF (red) and CSK (green) location in SVF cells after 48 h of rhLF treatment. Nuclei were labeled by DAPI (blue). Scale bar = 130 µm. (F, G) Western blot and quantification of CSK protein expression in SVF cells after 48h treated with 10 µg mL^−1^ rhLF (n = 4). (H, I) Western blot and quantification of CSK in CHX‐treated (10 µg mL^−1^) SVF cells with 10 µg mL^−1^ rhLF (n = 4). (J, K) Western blot and quantification of ubiquitination of CSK in HEK293T cells with 10 µg mL^−1^ rhLF (n = 3). Cells were pretreated with 10 µm MG132 for 6 h before harvest. (L–N) Western blot and quantification phosphorylation level of SRC at Tyr529 in SVF cells after 48 h treated with 10 µg mL^−1^ rhLF (n = 4). (O) The absorbance after adding CCK‐8 in NC or CSK^oe^ SVF cells with 10 µg mL^−1^ rhLF treated for 48h (n = 4). (P, Q) Western blot and quantification of CSK protein expression, and phosphorylation level of SRC at Tyr529 in NC or CSK^oe^ SVF cells with 10 µg mL^−1^ rhLF treated for 48 h (n = 4). (R) Relative mRNA levels of target genes in NC or CSK^oe^ SVF cells with 10 µg mL^−1^ rhLF treated for 48 h (n = 3). The group treated with 0 µg mL^−1^ rhLF served as the control. Data are mean ± SEM. Significance for Figure 5G,I,K,M,N was calculated using Student's two‐tailed unpaired *t*‐test, ^***^ *p* < 0.001, ^**^ *p* < 0.01, and ^*^ *p* < 0.05. Significance for Figure 5O,Q,R was calculated using two‐way ANOVA combined with Tukey's multiple comparison test. ^***^ *p* < 0.001, ^**^ *p* < 0.01 and ^*^ *p* < 0.05 indicate significant differences between groups; ^+++^ *p* < 0.001 indicate a significant main effect of rhLF treatment.

To explore the mechanism by which rhLF promotes SVF cell proliferation, we performed quantitative MS‐based proteomic analysis on rhLF immunoprecipitates from proliferating SVF cells. This identified CSK as a potential binding partner of rhLF (Figure 6A). Molecular docking predicted hydrogen bond interactions between LF and CSK at multiple residues (Figure 6B). Co‐immunoprecipitation (Co‐IP) confirmed their specific interaction (Figure 6C,D), which was supported by their colocalization in SVF cells (Figure 6E; Figure S5A).

Treatment with 10 µg mL^−1^ rhLF significantly reduced CSK protein levels without affecting its mRNA expression (Figure 6F,G; Figure S5B), indicating that rhlf may promote CSK protein degradation. To test this hypothesis, we evaluated the effect of rhlf on CSK protein stability in the presence of the protein synthesis inhibitor cycloheximide (CHX). The results showed that rhlf accelerated CSK protein degradation (Figure 6H,I). Subsequently, in the presence of CHX, we used either the lysosome inhibitor chloroquine (CQ) or the proteasome inhibitor MG132 to investigate the pathway through which rhlf promotes CSK degradation. Both CQ and MG132 were able to rescue the reduced half‐life of CSK induced by rhlf (Figure S5C–F). Furthermore, rhlf treatment significantly enhanced CSK ubiquitination (Figure 6J,K).

Given that CSK inhibits cell proliferation by phosphorylating SRC at Tyr529 [33], we assessed SRC activity. The results showed that rhlf treatment significantly reduced SRC phosphorylation at Tyr529 (Figure 6L–N).

To investigate whether CSK mediates the pro‐proliferative effect of rhLF, we examined cell proliferation in both normal control (NC) and CSK‐overexpressing (CSK^oe^) SVF cells in the presence or absence of rhLF. Notably, rhLF enhanced the proliferation of NC cells, but this effect was abolished in CSK ^oe^ cells (Figure 6O). Furthermore, CSK overexpression restored the rhLF‐induced decrease in SRC Tyr529 phosphorylation (Figure 6P,Q) and suppressed the upregulation of cell cycle genes (*Ccnb1*, *Cdk2*, *Cdk1*, *Cdk6*) induced by rhLF (Figure 6R).

### 2.7 rhLF Promotes SVF Cell Adipogenic Differentiation by Inhibiting PRMT5 Degradation to Activate the PRMT5‐PPARg Pathway In Vitro

**FIGURE 7:**
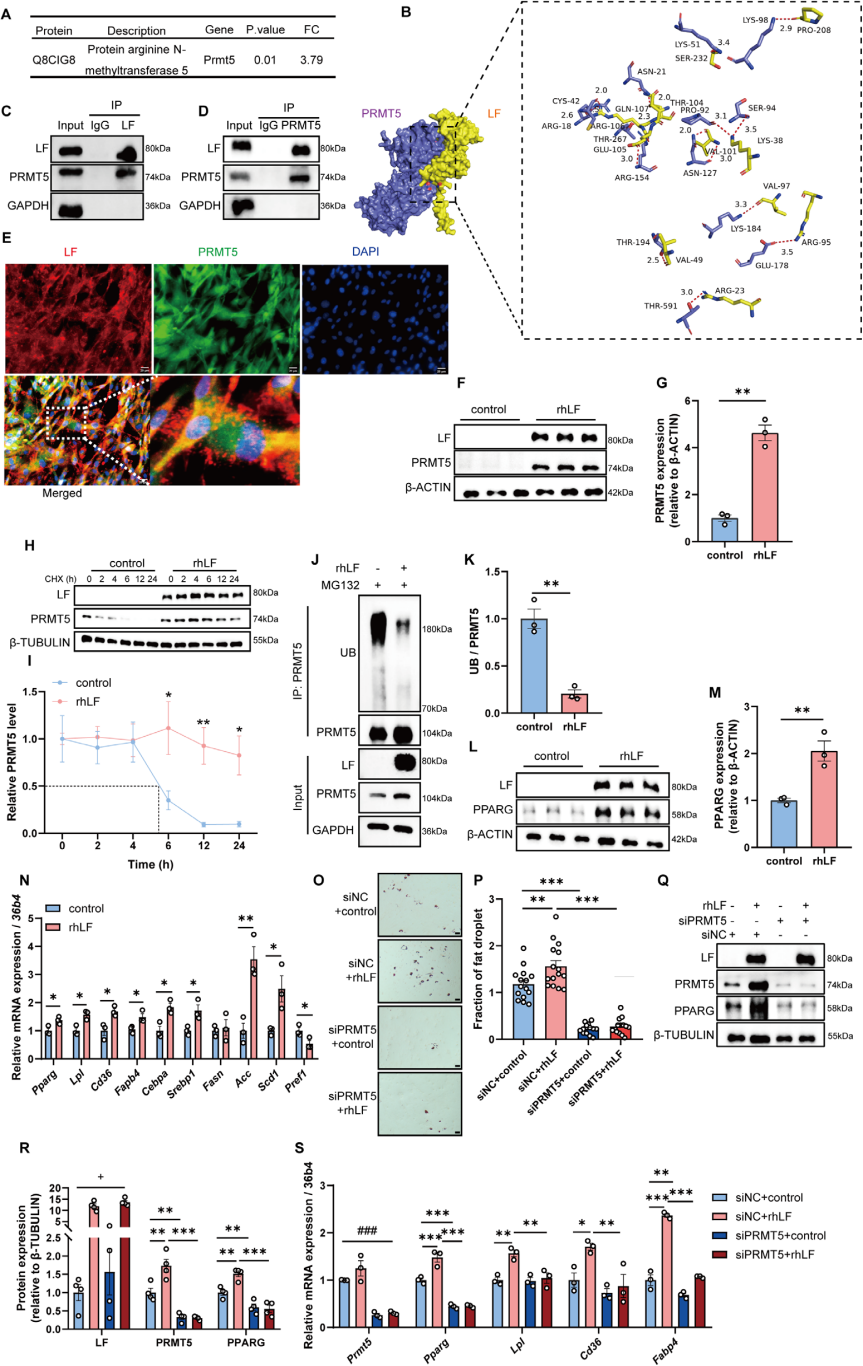
rhLF promotes SVF cells adipogenic differentiation via PRMT5‐PPARg pathway. (A) The PRMT5 protein in the IP product of rhLF was discovered through IP/MS analysis. (B) Molecular docking analysis of PRMT5 (Q8CIG8) and LF (Q8IX02). (C, D) Co‐IP analysis of the interaction between rhLF and PRMT5 in SVF cells was performed with anti‐PRMT5 or anti‐LF primary antibodies (n = 3). (E) Representative 100x immunofluorescence images of rhLF (red) and PRMT5 (green) location in SVF cells at the early stage of differentiation with rhLF treatment. Nuclei were labeled by DAPI (blue). Scale bar = 20 µm. (F, G) Western blot and quantification of PRMT5 protein expression in SVF cells at the early stage of differentiation after 48 h treated with 10 µg mL^−1^ rhLF (n = 3). (H, I) Western blot and quantification of PRMT5 in CHX‐treated (10 µg mL^−1^) SVF cells at the early stage of differentiation with 10 µg mL^−1^ rhLF (n = 4). (J, K) Western blot and quantification of ubiquitination of PRMT5 in HEK293T cells with 10 µg mL^−1^ rhLF (n = 3). Cells were pretreated with 10 µm MG132 for 6 h before harvest. (L, M) Western blot and quantification of PPARg protein expression in SVF cells at the early stage of differentiation after 48 h treated with 10 µg mL^−1^ rhLF (n = 3). (N) Relative mRNA levels of target genes in SVF cells at the early stage of differentiation with 10 µg mL^−1^ rhLF (n = 3). (O, P) ORO stain and quantification of the level of adipogenesis in control (siNC) or PRMT5 knockdown (siPRMT5) SVF cells after 8 days of adipogenic differentiation with 10 µg mL^−1^ rhLF treated at 0–2 days of differentiation (n = 15 fields of view counted per group). Scale bar = 50 µm. (Q, R) Western blot and quantification of PRMT5 and PPARg protein expression in siNC or siPRMT5 SVF cells at the early stage of differentiation with 10 µg mL^−1^ rhLF (n = 4). (S) Relative mRNA levels of target genes in siNC or siPRMT5 SVF cells with 10 µg mL^−1^ rhLF (n = 3). The group treated with 0 µg mL^−1^ rhLF served as the control. Data are mean ± SEM. Significance for Figure 5G,I,K,M,N was calculated using Student's two‐tailed unpaired *t*‐test, ^**^ *p* < 0.01, and ^*^ *p* < 0.05. Significance for Figure 5P,R,S was calculated using two‐way ANOVA combined with Tukey's multiple comparison test. ^***^ *p* < 0.001, ^**^ *p* < 0.01 and ^*^ *p* < 0.05 indicate significant differences between groups; ^###^ *p* < 0.001 indicate a significant main effect of PRMT5 knockdown; ^+^ *p* < 0.05 indicate a significant main effect of LF treatment.

To investigate how rhLF promotes healthy adipocyte development, we performed quantitative MS‐based proteomics on rhLF immunoprecipitates from SVF cells at early differentiation. This identified PRMT5 as a candidate rhLF‐binding partner (Figure 7A). Molecular docking predicted hydrogen bonding between LF and PRMT5 at multiple residues (Figure 7B). Co‐IP confirmed their specific interaction (Figure 7C,D), which was supported by their colocalization in SVF cells during early adipogenesis (Figure 7E; Figure S6A).

Treatment with rhLF increased PRMT5 protein levels without altering its mRNA levels (Figure 7F,G; Figure S6B), suggesting that rhLF may enhance PRMT5 stability. Consistent with this, rhLF extended the half‐life of PRMT5 in CHX chase assays (Figure 7H,I), and reduced its ubiquitination (Figure 7J,K).

Given that PRMT5 promotes adipogenesis by upregulating PPARg and its targets [34], we assessed this pathway. In this study, rhLF treatment significantly increased both the protein and mRNA levels of PPARg in early‐differentiating SVF cells (Figure 7L–N), and significantly increased its downstream genes (*Lpl*, *Cd36*) as well as synergistic adipogenic factors (*Srebp1*, *Cebpa*, *Acc*, and *Scd1*) (Figure 7N).

To investigate whether PRMT5 mediates the pro‐adipogenic effect of rhLF, we compared the adipogenic capacity of normal control (siNC) and PRMT5‐knockdown (siPRMT5) SVF cells in the presence or absence of rhLF. Notably, rhLF enhanced lipid accumulation in siNC cells, as shown by Oil Red O staining, but this effect was abolished upon PRMT5 knockdown (Figure 7O,P). PRMT5 knockdown also suppressed the rhLF‐induced upregulation of PPARg expression (Figure 7Q,R) and target genes (*Lpl*, *Cd36*, *Fabp4*) expression (Figure 7S).

## 3 Discussion

Obesity, characterized by pathological white adipose tissue (WAT) expansion, is closely linked to the development of metabolic diseases [35]. WAT expansion occurs through an increase in the size of existing adipocytes (hypertrophy) or the differentiation of adipocyte precursors into new adipocytes (hyperplasia) [36]. Hyperplasia of adipocyte is generally metabolically protective, whereas hypertrophy often leads to dysfunctional WAT remodeling and metabolic complications [37]. Therefore, identifying key regulators of healthy adipocyte development is crucial for preventing obesity‐related metabolic disorders. The lactation period is a critical window for adipose development, where nutritional status can exert long‐lasting effects on tissue formation and metabolic programming [12]. This study demonstrates that lactational LF deficiency in mice suppresses adipocyte hyperplasia, induces hypertrophy and adipose dysfunction, and triggers systemic metabolic disorders and inflammation. Mechanistically, LF promotes SVF cells proliferation by binding CSK and facilitating its ubiquitin‐mediated degradation, thereby activating SRC activity. Concurrently, LF enhances SVF cells differentiation by binding PRMT5, stabilizing it against ubiquitination, and potentiating PPARg signaling. Importantly, the adipose developmental defects and metabolic reprogramming caused by lactational LF deficiency persist into adulthood and obesity, highlighting the long‐term impact of early‐life nutrition.

LF, a multifunctional protein abundant in breast milk, is crucial for infant growth and development. LF concentration in human colostrum is notably high, exceeding 5 g L^−1^, and remains at 2–3 g L^−1^ in mature milk. In contrast, bovine milk contains lower levels, with 0.8 g L^−1^ in colostrum and only 0.03–0.49 g L^−1^ in mature milk [13]. Although regulators permit bovine LF in infant formula, its commercial levels (30–100 mg/100 g) remain far below the physiological levels in human milk [38].

Previous studies have investigated the intervention effects of LF in obesity and its associated metabolic disorders, including both preventive and therapeutic strategies. Animal studies have demonstrated that oral administration of LF effectively reduces body weight, fat accumulation, blood lipid, and glucose levels in HFD‐induced obese mice, while also ameliorating hepatic steatosis, intestinal inflammation, and systemic chronic inflammation [20, 39, 40, 41, 42]. Furthermore, lactoferrin has shown beneficial effects in improving obesity‐related vascular dysfunction [43], non‐alcoholic fatty liver disease [20, 42], and in combination therapies (e.g., with hypoxia, metformin, or fermented dairy products) [19, 44, 45]. Clinical studies have also suggested that lactoferrin supplementation can reduce visceral fat in obese individuals, improve body mass index and glucolipid metabolism markers, and decrease inflammatory levels [46, 47]. Here, we focus on the lactation period, which serves as a crucial developmental window for eWAT. Our previous studies showed that lactational LF deficiency exacerbates gut microbiota dysbiosis in obese mice [48]. Here, despite lower body weight and eWAT mass after 8‐week HFD, LF‐deficient mice displayed worse metabolic phenotypes and eWAT dysfunction, suggesting lactational LF deficiency exacerbates obesity‐related metabolic dysregulation, potentially via early developmental defects in eWAT.

By constructing a single‐nucleus transcriptomic atlas of eWAT across lactation, adulthood, and prolonged DIO, we systematically elucidated how lactational LF deficiency affects eWAT cellular composition and plasticity. We found that the proportion of newly formed adipocytes declines with age, in contrast to the peak adipogenesis during lactation. In obese mice, the adipose tissue was predominantly composed of pro‐inflammatory adipocytes, with a lower proportion of new adipocytes. Although adipocyte expansion under obesity can involve both hyperplasia and hypertrophy, existing studies indicate that long‐term HFD significantly suppresses the hyperplastic process [49]. Notably, lactational LF deficiency exacerbated the reduction of newborn adipocytes and increase in pro‐inflammatory adipocytes in obese mice, a change detectable as early as 3 weeks of age. Furthermore, LF deficiency reduced APC while increased FAP in both 3‐week‐old and obese mice, suggesting that LF loss compromises adipose plasticity by impairing adipogenic potential and reducing adipocyte turnover.

Notably, across all stages, adipocyte DEGs from LF deficiency were enriched in the PPAR signaling pathway. Genes related to lipid uptake were consistently downregulated, suggesting a central role for lipid uptake in LF‐mediated regulation of adipocyte development and metabolism. Exogenous lipid uptake serves as a major source for lipid storage in adipocytes, which also possess DNL capacity [50]. We found that lactational LF deficiency promoted DNL in 3‐week and 9‐week‐old mice, an unexpected shift in lipid metabolic patterns. Enhanced hepatic DNL is typically associated with metabolic dysfunction [51]. While some studies suggest that adipocyte DNL might be a marker of insulin sensitivity [11], its upregulation here likely reflects a compensatory response to defective lipid uptake. In contrast, this compensatory mechanism was absent in ko‐ko mice, where *Srebp1* and *Fasn* expression were significantly lower than those in wt‐ko mice. This difference suggests that the endogenous LF gene is required for adipose tissue to initiate compensatory lipogenesis under nutritional stress. Furthermore, the lower expression level of *Lep*, a key marker of adipocyte maturation, in KO mice further supports a role of endogenous LF in adipose development. Consistent with studies by Moreno‐Navarrete et al. [21], when LF gene deficiency impairs the differentiation potential of adipocyte precursors, the cells fail to initiate *Srebp1*‐mediated adipogenic responses. However, exogenous LF intake can partially restore the adipogenic capacity of KO mice.

Importantly, impaired lipid clearance in adipocytes, the primary lipid‐storing cells, may indicate functional compromise [6]. *Retn* is associated with insulin resistance [52]. In ko‐wt mice, which possess an intact genotype but lack exogenous LF, the elevated expression of *Retn* likely reflects a compensatory stress response of adipose tissue. In contrast, in ko‐ko mice with LF gene deficiency, adipocyte maturation is impaired due to defective differentiation, resulting in markedly reduced *Retn* expression. *Adipoq*, an important indicator of adipocyte health [53], is primarily influenced by lactational LF intake rather than by the LF genotype of pups. Likewise, the number of eWAT adipocytes is mainly regulated by exogenous LF intake during lactation. These findings suggest that exogenous LF acts as a key nutritional signal that directly modulates the metabolic status and function of adipocytes. Consistent with previous studies that reported low LF expression in adipose tissue [54], our snRNA‐seq analysis also revealed low LF expression across all cell populations, further supporting the central role of exogenous LF in adipocyte development and maturation. Moreover, Lactational LF deficiency induces adipocyte hypertrophy and apoptosis at the transcriptional level in mice at various developmental stages, providing additional evidence that the absence of exogenous LF intake leads to eWAT adipocyte dysfunction.

This study reveals the long‐term regulatory effects of lactational LF deficiency on adipocyte development. In vivo, lactational LF deficiency caused a lasting phenotype of inhibited eWAT adipocyte hyperplasia, impaired lipid uptake, and dysregulated adipokine expression. Although eWAT weight significantly increased in mice with lactational LF deficiency after 20 weeks of HFD feeding, which was contrary to the reduced eWAT observed after 8 weeks of HFD, we speculate that prolonged HFD feeding may drive further adipocyte hypertrophy in dysfunctional eWAT, leading to more pronounced visceral fat accumulation [6]. Except for the changes in eWAT, LF deficiency promoted pWAT accumulation and reduced iWAT mass, thereby shifting body fat distribution toward a pathological pattern of increased visceral adiposity that is strongly associated with metabolic dysregulation [55]. Meanwhile, the lower BAT mass seen in young mice even before HFD feeding suggests a limited ability to expend energy, which likely contributed to their poor metabolic health. In addition, lactational LF deficiency still led to impaired glucose tolerance, exacerbated insulin resistance, elevated serum lipid and inflammatory factor levels, accompanied by increased macrophage infiltration in eWAT and worsened hepatic steatosis. The ectopic fat deposition in organs like the liver is usually indicative of dysfunction of the adipose tissue characterized by insufficient lipid storage capacity [8]. These findings indicate that LF deficiency causes adipose tissue dysfunction, resulting in insufficient lipid storage capacity under conditions of nutrient excess, thereby promoting ectopic lipid deposition in the liver and exacerbating metabolic disturbances. Importantly, rhLF addition to SVFs promoted proliferation, adipogenic differentiation, and lipid uptake, supporting a cell‐autonomous effects of LF on adipocyte development. Moreover, this effect is not unique to mice, as rhLF also promoted the proliferation and adipogenic differentiation of hMSCs.

We identified that the decreased stability of CSK as key molecular mechanism underlying the function of LF in the proliferation of primary adipocyte. CSK is a ubiquitously expressed non‐receptor tyrosine kinase that negatively regulates SRC activity by phosphorylating the C‐terminal tyrosine residue of the SRC protein [33]. As a protein tyrosine kinase, SRC can activate multiple downstream signaling pathways, including PI3K/AKT and MAPK, playing crucial roles in regulating cell survival and function [56]. Activation of SRC promotes the expression of cell cycle proteins and accelerates the G1‐to‐S phase transition, thereby stimulating cell proliferation. IGF‐1 reportedly promotes 3T3‐L1 proliferation by downregulating CSK and inhibiting SRC pTyr529 [57]. Using IP‐MS, we identified CSK as a binding target of rhLF. We subsequently confirmed that LF binds CSK and promotes its ubiquitin‐dependent degradation, leading to reduced SRC Tyr529 phosphorylation, enhanced SRC kinase activity, and ultimately increased adipocyte precursor proliferation. However, the precise ubiquitination mechanism requires further study.

Notably, we did not observe significant changes in *Pparg* expression in eWAT following lactational LF deficiency. Further snRNA‐seq analysis indicated that *Pparg*, while not being a DEG, was highly expressed in a large proportion of adipocyte subpopulations, potentially masking its expression changes in other cell populations. However, *Pparg* was significantly down‐regulated in the ASPC subcluster of obese mice upon LF deficiency. Lactational LF deficiency led to enrichment of DEGs in the PPAR signaling pathway within the ASPC population of obese mice, accompanied by down‐regulation of lipid uptake‐related genes and markers of newborn adipocyte. Moreover, lactational LF deficiency also decreased the expression of lipid uptake‐related genes in the ASPC population of 3‐week‐old mice. These findings highlight the central role of exogenous LF in determining the fate of adipose precursor cells. After receiving adipogenic signals, adipocyte precursor cells and adipose‐derived stem cells exit the cell cycle and initiate differentiation, ultimately forming mature adipocytes capable of lipid storage [58]. We demonstrated that enhanced PRMT5 stability is a primary molecular mechanism for LF's role in adipocyte precursor adipogenic differentiation. PRMT5, an arginine methyltransferase involved in chromatin remodeling, has been demonstrated to be indispensable for adipogenesis. PRMT5 promotes PPARg expression and its target genes during adipogenesis [34]. PPARg, a key lineage‐determining transcription factor essential for adipogenesis, forms differentiation‐dependent chromatin loops between its promoter and enhancers during differentiation, a process requiring PRMT5 [59]. Using IP‐MS, we identified PRMT5 as a binding target of rhLF in early‐differentiating SVF cells. This finding is correlating perfectly with the frequent enrichment of the PPAR signaling pathway in our eWAT snRNA‐seq data, further emphasizing the centrality of this pathway in LF‐mediated adipogenesis. We subsequently confirmed that LF binds PRMT5 and inhibits its ubiquitin‐dependent degradation in early‐differentiating SVF cells, thereby promoting PPARg expression, enhancing lipid uptake capacity, and synergistically facilitating healthy development of adipocyte. However, the specific mechanisms and sites by which LF binding inhibits PRMT5 ubiquitination remain to be elucidated.

While this study has focused on the roles of LF in binding CSK and PRMT5 within the SVF during proliferation and early differentiation, we made a pertinent observation regarding its subcellular localization. We found that exogenous LF primarily localizes to the cytoplasm during these initial stages. Interestingly, as differentiation progresses, LF appears to accumulate increasingly within the nucleus. Given that LF is a protein containing a nuclear localization signal [60], this shift in localization suggests that, particularly during later stages of adipocyte development, LF may exert additional functions beyond those identified here.

eWAT is a complex functional ensemble composed of multiple cell types [61]. Lactational LF deficiency increased the abundance of inflammatory EC and enhanced oxidative stress responses in obese mice, likely representing adaptations to metabolic stress and reflecting a deteriorated pro‐inflammatory local microenvironment [62]. Inflammation typically leads to abnormal enhancement of the systemic immune response [63]. HFD shifted macrophage composition and transcriptional profiles toward a lipid‐associated phenotype, which aligned with the recent studies [11, 31]. In obese mice with lactational LF deficiency, macrophages exhibited decreased transcriptional signatures for both lipid uptake and catabolism, potentially impairing their ability to assist in lipid clearance and maintain adipose tissue homeostasis. The relative abundance of RMs, which improve adipocyte insulin sensitivity, was reduced in both 3‐week‐old pups and obesity mice, further exacerbating the disruption of adipose tissue homeostasis. Importantly, LF deficiency established a pro‐inflammatory, metabolically compromised transcriptional profile in macrophages early in development, exerting lasting effects on the adipose micro‐environment.

Lactational LF deficiency caused persistent dysregulation of the adipokines *Adipoq* and *Retn* in mouse eWAT. Adiponectin is an adipokine with anti‐inflammatory, anti‐diabetic, and anti‐atherosclerotic effects [53]. Conversely, Ressitin contributes to insulin resistance, inflammation, and metabolic disorders by interfering with insulin signaling pathways [52]. This early reprogramming persistently impacts all cells. In obesity, epithelial cells and immune cells may synergize to create a pro‐inflammatory microenvironment. This disrupts homeostasis in the tissue under lactational LF deficiency, and hypertrophic adipocytes continue to recruit immune cells, intensifying local inflammation and creating a self‐perpetuating vicious cycle that ultimately leads to severe metabolic disturbances locally in adipose tissue and systemically [7]. Additionally, lactational LF deficiency induced the expression of extracellular matrix proteins in ASPCs of obese mice, and the pro‐inflammatory microenvironment shaped by macrophages may further drive ASPCs toward a pro‐fibrotic phenotype [30]. This further reduces adipocyte turnover efficiency and inhibits adipose tissue renewal in LF‐deficient mice. These findings illustrate that adipose tissue is a tightly interacting system, and lactational LF deficiency reduces its plasticity through multiple pathways. Further research could explore in depth intercellular communication and the specific roles of different cellular subpopulations in LF‐regulated adipose tissue plasticity.

The systemic metabolic phenotypes in our lactational LF deficiency models are consistent with the concept that visceral WAT health determine systemic metabolic homeostasis [37]. Promoting adipocyte hyperplastic expansion and facilitating healthy WAT remodeling are increasingly recognized as important strategies for combating obesity‐related metabolic diseases. Furthermore, our findings support the concept that early‐life homeostasis persistently influences adult chronic disease [10]. Therefore, from a clinical perspective, lactational LF supplementation may promote healthy adipose development, offering a potential nutritional intervention strategy to prevent WAT dysfunction or improve WAT function and systemic metabolic health in obese individuals.

In summary, this study identifies a critical role for LF in lactational adipose tissue development, metabolic programming, and plasticity. We demonstrate that deficiency in lactational LF intake suppresses adipocyte hyperplasia, promotes hypertrophy, and disrupts immune homeostasis, leading to persistent metabolic dysfunction exacerbated by obesity. LF promotes adipocyte generation by inhibiting the CSK‐SRC pathway, and facilitates healthy adipocyte development by stabilizing PRMT5 to improve PPARg expression. Our findings reveal the profound long‐term impact of LF as an early‐life nutrient, and provide a mechanistic rationale for LF supplementation during lactation as a strategy to improve adipose tissue function and prevent obesity‐related metabolic diseases.

## 4 Experimental Section

### 4.1 Animals and Experimental Models

The *Lf* gene knockout (KO) mice were customized from Biocytogen Co., Ltd. (Beijing, China). Researchers utilized CRISPR‐Cas9 technology to delete exons 3–8 of the *Lf* gene in zygotes of C57BL/6N mice, successfully establishing a systemic *Lf* gene knockout model [64]. Single guide RNA sequences were designed targeting intronic regions 3 and 8, inducing a deletion of approximately 4 kb in the genomic fragment, thereby achieving knockout of the target gene.

Before the experiment, KO and wild‐type (WT) mice were bred synchronously. The lactational LF deficiency model was constructed according to our previous method [17], WT newborns were cross‐fostered to lactating KO dams on postnatal day 2, thus generating the ko‐wt group, which suckled milk lacking LF. The control group (wt‐wt) comprised WT pups suckled by WT dams. Additionally, KO pups suckled by WT dams to form the wt‐ko group, while KO pups suckled by KO dams to establish the ko‐ko group.

Pups were weaned on postnatal day 21 and subsequently fed a standard maintenance diet until 9 weeks of age. Starting at 9 weeks of age, mice from the ko‐wt and wt‐wt groups were fed a high‐fat diet (HFD) (60% kcal from fat, Xietong, Jiangsu, China, Cat. No: XTM04‐001) for either 8 or 20 weeks to establish the obesity model. Control (CON) groups for both ko‐wt and wt‐wt continued to receive the standard maintenance diet (6% kcal from fat, Huafukang Bioscience, Beijing, China, Cat. No: 1025). Only male mice were used in this study.

All mice were housed under standardized conditions (temperature 22 ± 2°C, relative humidity 55 ± 10%, 12 h light/dark cycle) with free access to food and water. The animal experiments involved in this study were conducted in accordance with the guidelines of the Animal Care and Use Committee of China Agricultural University (SYXK 2020‐0052), and were approved by the Animal Experiment Ethics Committee of China Agricultural University (approval number: Aw40702202‐4‐5, approval date: July 4, 2022).

### 4.2 Glucose and Insulin Tolerance Tests

Glucose tolerance test (GTT) and insulin tolerance test (ITT) were performed according to previous studies [65]. For GTT, mice were fasted for 16 h and then injected intraperitoneally with a glucose (Sigma, St. Louis, MO, USA, Cat. No: G7021) at a dose of 1.5 g kg^−1^ body weight. For ITT, mice were fasted for 6 h and then injected intraperitoneally with bovine insulin (Labled, Beijing, China, Cat. No: I5500) at a dose of 1 U kg^−1^ body weight. In both tests, Glucose was monitored in the tail blood at 0, 15, 30, 60, 90, and 120 min after injection.

### 4.3 Serum Analysis

After anesthesia with 1.25% tribromoethanol (TIGERGENE, Nanjing, China, Cat. No: 2506B), blood samples were collected from the retro‐orbital venous plexus of experimental animals. Whole blood was centrifuged at 5000 rpm for 20 min at 4°C, and the supernatant serum was collected. Serum metabolic parameters, including TG, TC, HDL, LDL, vLDL, TNF‐α, IL‐6, MCP‐1, Adiponectin (ADP), and Resistin (RES), were analyzed by Huaying Biological Technology Research Institute (Beijing, China).

### 4.4 Histological and Morphometric Analysis

Mice were euthanized, and tissues were collected at three different time points: 3 weeks of age, 9 weeks of age, and after HFD‐induced obesity (DIO). Mice at 3 weeks of age (wt‐wt, ko‐wt, wt‐ko, and ko‐ko groups) were euthanized under isoflurane anesthesia without fasting. Mice at 9 weeks of age (ko‐wt and wt‐wt groups) and HFD‐induced obese mice (CON wt‐wt, CON ko‐wt, HFD wt‐wt, and HFD ko‐wt) were fasted overnight and then anesthetized with 1.25% tribromoethanol prior to euthanasia. Afterward, organs were dissected out and weighed.

Adipose tissue and liver were fixed in 4% paraformaldehyde for at least 24 h, followed by graded dehydration and paraffin embedding [64]. Using a Leica microtome (Wetzlar, Germany), adipose tissue blocks were sectioned at a thickness of 8 µm, while liver blocks were sectioned at a thickness of 5 µm. The sections were deparaffinized programmatically and stained using hematoxylin and eosin (H&E) staining kit (Beyotime, Shanghai, China, Cat. No: C0105S).

Bright‐field microscopy was used to capture images of the tissue sections, with at least six random fields selected per sample. ImageJ software (NIH, Bethesda, MD, USA) was used to quantitatively analyze hepatic lipid droplet vacuole area and adipocyte area.

The total adipocyte number was determined according to the method described by Lemonnier [66]. Briefly, we calculated the average adipocyte volume using the formula π/6×(3σ^2^×d+d^3^), where d is the mean diameter and σ is the standard deviation of the diameter. This volume was then converted to the average adipocyte weight using a density of 0.92 g mL^−1^. Finally, the total adipocyte number was derived by dividing the total eWAT weight by the average adipocyte weight.

### 4.5 Immunofluorescence

Paraffin sections were deparaffinized, rehydrated, and subjected to antigen retrieval using enhanced citrate antigen retrieval solution (Beyotime, Shanghai, China, Cat. No: P0083) with microwave heating. Cells were fixed with 4% PFA for 15 min. After Phosphate Buffered Saline (PBS) washing, samples were permeabilized with 0.5% Triton X‐100 at room temperature for 15 min. Next, the samples were blocked with 10% donkey serum at room temperature for 1 h. Afterward, the sections were incubated with primary antibodies overnight at 4°C. After washing 3 times with PBS, followed by incubation for 1 h at room temperature with secondary antibodies in a dark place. Cell nuclei were stained with DAPI (Servicebio, Wuhan, China, Cat. No: G1012), and cell membranes were labeled with Wheat Germ Agglutinin (WGA) (Sigma, USA, Cat. No: L4895)

Crown‐like structures (CLS) in adipose tissue were analyzed using ImageJ. At least six random fields per sample were selected for quantification based on the following two parameters: the percentage of F4/80‐positive area per field and the ratio of F4/80‐positive cells to the total number of adipocytes [67].

The following primary antibodies were used: rabbit anti‐LF (Invitrogen, USA, Cat. No: PA595513), rabbit anti‐F4/80 (Cell Signaling Technology, Cat. No: 30325), rabbit anti‐PRMT5 (HUABIO, Hangzhou, China, Cat. No: ET160943), and mouse anti‐CSK (Santa Cruz, Cat. No: sc166560).

The following secondary antibodies were used: 488‐conjugated Goat Anti‐Rabbit IgG (Servicebio, Wuhan, China, Cat. No: GB25303), 488‐conjugated Goat Anti‐Mouse IgG, Servicebio, Wuhan, China, Cat. No: GB25301), Cy5‐conjugated Goat Anti‐Rabbit IgG, Servicebio, Wuhan, China, Cat. No: GB27303), Cy5‐conjugated Goat Anti‐Mouse IgG, Servicebio, Wuhan, China, Cat. No: GB27301).

### 4.6 RNA sequencing

The eWAT from wt‐wt and ko‐wt mice after 8 weeks HFD feeding was collected, and the total RNA was extracted by using TRIZOL reagent (Invitrogen, USA). The concentration and purity of the RNA were detected by Nanodrop2000, RNA integrity was detected by agarose gel electrophoresis, the RIN value was measured by Agilent2100. The construction of a single data base requires that the total amount of RNA ≥ 1 µg, concentration > 35 ng µL^−1^, OD260/280 = 1.8∼2.2, OD260 /230 ≥ 2.0. RIN ≥ 6.5, 28S: 18S ≥ 1.0, Libraries were constructed using the Illumina TruseqTM RNA sample prep Kit (Illumina, San Diego, CA, USA) according to the manufacturer's instructions.

Sequencing of the libraries was performed on an Illumina NovaSeq X Plus instrument by Shanghai Majorbio Biopharm Biotechnology (Shanghai, China), and individually assessed for quality using FastQC. To identify DEGs between two different samples, the expression level of each transcript was calculated according to the transcripts per million reads (TPM) method. RSEM was used to quantify gene abundances. Differential expression analysis was performed using edgeR with a Q value ≤ 0.05. Statistical significance was assessed using a negative binomial Wald test, then corrected for multiple hypothesis testing with the Benjamini‐Hochberg method.

### 4.7 Single‐Nucleus RNA Sequencing

eWAT was isolated from mice at 3 weeks, 9 weeks, and after 20 weeks of HFD feeding, and stored at ‐80°C. For single‐nucleus RNA sequencing (snRNA‐seq) library preparation, we adopted a pooling strategy to minimize individual biological variability and obtain a representative cellular atlas. Specifically, each sequencing library was constructed from pooled eWAT samples derived from 20 mice for the 3‐week group, and from 6 mice each for the 9‐week and 20‐week HFD groups. This pooling approach has been widely used in single‐cell sequencing studies of heterogeneous tissues such as adipose tissue [68, 69, 70, 71]. Nuclei extraction and snRNA‐seq were performed by Novogene Bioinformatics Technology Co., Ltd (Tianjin, China). Frozen eWAT was homogenized in lysis buffer using a glass homogenizer. After 5 min of lysis, samples were filtered through a 30 µm strainer and centrifuged at 500 ×g for 5 min at 4°C. Nuclei pellets were washed, resuspended, and purified using a 29% Optiprep cushion by centrifugation at 10 000 ×g for 30 min. Purified nuclei were resuspended in Nuclei Resuspension Buffer and processed using the 10x Genomics Single Cell 3′ Kit (v4, PN:1000691) according to the manufacturer's instructions. Nuclei were loaded onto a 10x Chromium Chip (v4, PN:1000690) for barcoding with the Chromium Controller. Libraries were constructed with the 10x Library Construction Kit (v4, PN:1000694) according to the manufacturer's instructions. Sequencing was performed on an Illumina NovaSeq platform using a paired‐end 150 bp (PE150) configuration, with a target depth of 50 000–100 000 raw reads per nucleus.

### 4.8 snRNA‐seq Analysis

Unique molecular identifiers (UMIs) were employed to assign a unique tag to each original mRNA molecule. Following sequencing, the original mRNA abundance was estimated via UMI counts, and a gene was defined as expressed in a cell if its UMI count was ≥ 1. Sequencing data from each sample were aligned to the mm10 mouse reference genome (mm10‐2020‐A) using Cell Ranger (v7.1.0) to generate the feature‐barcode matrix. Stringent quality control was applied: nuclei with fewer than 200 detected genes or abnormally high gene counts (exceeding the 99th percentile of the distribution) were excluded. Nuclei with mitochondrial or erythrocyte gene content above the 99th percentile were also removed. Low‐abundance genes detected in fewer than three nuclei were filtered out, and doublets were removed using DoubletFinder for the 3‐week and 9‐week samples. After quality control, the data were analyzed with Seurat for clustering, dimensionality reduction, and differential expression. To mitigate batch effects, we integrated the count matrices from all samples using Canonical Correlation Analysis (CCA). The final dataset included 15900 cells (wt‐wt) and 12192 cells (ko‐wt) from 3‐week‐old mice; 7290 cells (wt‐wt) and 12000 cells (ko‐wt) from 9‐week‐old mice; and 11964 cells (HFD wt‐wt) and 11984 cells (HFD ko‐wt) from HFD‐induced obese mice.

For cell clustering, visualization, and annotation, the integrated dataset was analyzed using the top 2000 highly variable genes. Unsupervised clustering was performed based on the top 20 principal components, with resolution parameters tested between 0.2 and 1. Cell clusters were visualized in two dimensions using nonlinear dimensionality reduction techniques (t‐SNE, UMAP). Cluster‐specific marker genes were identified using the Wilcoxon rank‐sum test on the filtered expression matrix generated by Seurat.

Cell subtypes were annotated by integrating pathway analysis of marker genes (using the clusterProfiler R package, v3.14.0) with previously published cell‐type signatures. For differential expression analysis between ko‐wt and wt‐wt groups at each age, genes with a |log2FC| > 0.18–0.25 and an adjusted *p*‐value < 0.01 were considered significant. These differentially expressed genes were subsequently subjected to KEGG pathway enrichment analysis using clusterProfiler.

Additionally, pseudotemporal ordering of adipocyte subtypes was inferred using Monocle2 (v2.26.0).

### 4.9 Quantitative Real‐Time PCR (qRT‐PCR)

RNA was isolated using Trizol (Lablead, Beijing, China, Cat. No: R1100) according to the manufacturer's instructions. The purity and concentration of RNA were determined using a NanoDrop spectrophotometer (Thermo Fisher, Waltham, MA). 1 µg of total RNA was reverse transcribed into cDNA using a First‐strand cDNA Synthesis Mix kit (Lablead, Beijing, China, Cat. No: F0202). qRT‐PCR was performed using Rotor‐Gene Q (Qiagen, Hilden, Germany) with a SuperFastStar Universal Probe Mixture kit (Cwbio, Jiangsu, China, Cat. NO CW3380S). Values were normalized to *36b4* levels using the 2^−ΔΔCt^ method. The sequencing primers used in this study are listed in Table S1.

### 4.10 Western Blotting

Proteins were extracted with RIPA lysis buffer (Beyotime, Jiangsu, China, Cat. No: P0013B) supplemented with protease inhibitor (Beyotime, Jiangsu, China, Cat. No: ST507) and phosphatase inhibitor (Beyotime, Jiangsu, China, Cat. No: P1045). Protein samples and the loading buffer were mixed at a ratio of 4:1 and boiled for 10 min After separating the proteins using SDS‐PAGE (8%–10%), the protein strip was transferred to a PVDF membrane. The membranes were blocked using 5% non‐fat milk powder in Tris‐buffered saline and Tween (TBST) and incubated with primary antibodies at the indicated dilutions overnight at 4°C. After washing three times with TBST, followed by incubation with secondary antibody for 1 h, Protein bands were detected using Enhanced Chemiluminescence liquid (ECL) (Lablead, Beijing, China, Cat. No: E1050), and densitometry was performed by ImageJ software. The band intensity of each target protein was normalized to that of GAPDH/ β‐Tubulin/ β‐ACTIN.

The following primary antibodies were used: anti‐LF (Invitrogen, USA, Cat. No: PA595513), anti‐PRMT5 (HUABIO, Hangzhou, China, Cat. No: ET160943), anti‐PPARg ((UpingBio, China, Cat. No: YP‐mAb‐03325), anti‐CSK (UpingBio, China, Cat. No: YP‐Ab‐14713), anti‐p‐Src (Y529) (Abways, China, Cat. No: CY9049), anti‐SRC (UpingBio, China, Cat. No: YP‐Ab‐14716), anti‐Ubiquitin (Abways, China, Cat. No: CY5520), anti‐GAPDH (Lablead, Beijing, China, Cat. No: G0100), anti‐HISTONE H3.1 (Lablead, Beijing, China, Cat. No: H0101), anti‐β‐ACTIN (Servicebio, Wuhan, China, Cat. No: GB11001‐100), anti‐β‐TUBULIN (Lablead, Beijing, China, Cat. No: T0100).

The following secondary antibodies were used: Goat anti‐rabbit IgG (ABclonal, Wuhan, China, Cat. No:AS014) or Goat anti‐mouse IgG (ABclonal, Wuhan, China, Cat. No:AS003).

### 4.11 Immunoprecipitation

Proteins were extracted using Cell Lysis Buffer for Western and IP (Beyotime, Jiangsu, China, Cat. No: P0013J) containing protease inhibitor and phosphatase inhibitors. After lysis, samples were centrifuged at 13 000 ×g for 5 min at 4°C, and the supernatant was collected. The supernatant was incubated with the target‐specific primary antibody overnight at 4°C with rotation. Protein A+G Magnetic Beads (Beyotime, Jiangsu, China, Cat. No: P2108) were added the next day and incubated for 1 h at room temperature. The beads were collected using a magnetic stand, and the supernatant was discarded. The immunoprecipitations were washed three times with PBST. After washing, proteins were eluted by heating at 95°C for 5 min in 2× protein loading buffer. Controls included input (whole cell lysate without immunoprecipitation) and IgG control (immunoprecipitation with normal IgG). Immunoprecipitated proteins were subsequently detected by western blot using target‐specific primary antibodies and HRP‐conjugated secondary antibodies, followed by band visualization and analysis.

The following antibodies were used: anti‐LF (Santa Cruz, Cat. No: sc‐53498), anti‐PRMT5 (HUABIO, Hangzhou, China, Cat. No: ET160943), anti‐CSK (Santa Cruz, Cat. No: sc166560), mouse IgG (Beyotime, Jiangsu, China, Cat. No: A7028), rabbir IgG (Cat. No: A7016).

### 4.12 Isolation, Culture, and Adipogenic Differentiation of SVF

The stromal vascular fraction (SVF) was isolated from eWAT of 3–4‐week‐old mice [68]. Minced tissues were digested with 1 mg mL^−1^ collagenase I (Lablead, Beijing, China, Cat. No: V1891) at 37 °C for 40–60 min. The digestion was stopped by adding DMEM (Vivacell, Shanghai, China, Cat. No: 01‐052‐1ACS) supplemented with 10% FBS (Vistech, New Zealand, Cat. No: SE200‐ES). The mixture was centrifuged at 1000 ×g for 5 min, and the pellet was resuspended in high‐glucose DMEM complete medium (10% FBS, 1% penicillin–streptomycin), filtered through a 70 µm strainer, and centrifuged again. Cells were then resuspended and cultured in flasks at 37 °C with 5% CO_2_, with medium changes every other day.

Upon reaching 80%–90% confluence, SVF cells were passaged using 0.25% trypsin and seeded into culture plates for subsequent experiments. For adipogenic differentiation, cells were maintained for 48 h after reaching contact inhibition (designated as Day 0). The medium was then replaced with adipogenic induction medium (containing 2 µm rosiglitazone, 1 µm dexamethasone, 0.5 mm IBMX, and 10 µg mL^−1^ insulin) for 2 days, followed by adipogenic maintenance medium (with 10 µg mL^−1^ insulin) until Day 8, with medium refreshed every two days.

To examine the effect of LF on SVF proliferation, cells were treated with recombinant human lactoferrin (rhLF) (donated by Dr. Yunping Dai research group, China Agricultural University, China) dissolved in PBS during the proliferation phase. To assess the role of rhLF in adipogenesis, cells were treated with rhLF either throughout the differentiation process or at specific stages. Controls received PBS only.

### 4.13 Culture, and Adipogenic Differentiation of hMSCs

Immortalized human adipose‐drived mesenchymal stem cells (hMSCs) (FuHeng, Shanghai, China, Cat. No: Y008) were cultured in DMEM/F12 complete medium (10% FBS, 1% penicillin–streptomycin) at 37 °C with 5% CO_2_, with medium changes every other day. Upon reaching 80%–90% confluence, cells were passaged using 0.25% trypsin and seeded into culture plates for subsequent experiments. For adipogenic differentiation, cells were maintained for 48 h after reaching contact inhibition (designated as Day 0). The medium was then replaced with adipogenic induction medium for 4 days, followed by adipogenic maintenance medium until Day 10, with medium refreshed every two days.

To examine the effect of LF on hMSCs proliferation, cells were treated with rhLF during the proliferation phase. To assess the role of rhLF in adipogenesis, cells were treated with rhLF throughout the differentiation process. Controls received PBS only.

### 4.14 CCK‐8 Assay

Cells were seeded in 96‐well plates at a density of 2 × 10^3^ cells per well and cultured under standard conditions (37°C, 5% CO_2_). After 24 h of adhesion, the medium was replaced with fresh medium containing rhLF at final concentrations of 0.01, 0.1, 1, 10, 100, and 200 µg mL^−1^. Control wells received medium without rhLF.

Following 24, 48, or 72 h of treatment, cell proliferation was assessed using a CCK‐8 kit (Lablead, Beijing, China, Cat. No: CK001) according to the manufacturer's instructions. The absorbance of each well was measured at 450 nm using a microplate reader (Aosheng, Hangzhou, China).

### 4.15 Oil Red O (ORO) Staining

To assess adipogenic differentiation, cells were treated with rhLF at final concentrations of 0.01, 0.1, 1, 10, 100, and 200 µg mL^−1^. Treatments were administered either throughout the entire differentiation period (days 0–8) or during specific intervals (days 0–2, 2–4, 4–6, or 6–8), with all groups analyzed on day 8. Controls received PBS only.

At the end of the adipogenic stage, cells were fixed with 4% paraformaldehyde for 15 min at room temperature and washed three times with PBS. Subsequently, they were stained with 3 mg mL^−1^ Oil Red O (Lablead, Beijing, China, Cat. No: 0684) solution for 30 min at room temperature. After staining, cells were briefly rinsed with 60% isopropanol to remove unbound dye. Stained lipid droplets were visualized and imaged under a microscope. Quantification of ORO staining results can be achieved by either calculating the fraction of ORO‐stained area per field of view, or by eluting the intracellular ORO dye with isopropanol and measuring the absorbance at 510 nm.

### 4.16 Fatty Acid Uptake Assay

Fatty acid (FA) mixture was prepared by conjugating palmitic acid (Sigma, USA, Cat.No: P5585) and oleic acid (Sigma, USA, Cat. No: O1008) at a 1:2 molar ratio to 25% FA‐free BSA (Beyotime, Jiangsu, China, Cat. No: ST025) in PBS. On day 8 of adipogenic differentiation, SVF cells were switched to fresh complete medium containing 1 mm of the FA mixture and incubated for 24 h; control cells received BSA solution only.

To assess fatty acid uptake, cells were incubated with 1 µm BODIPY‐labeled Lauric Acid (Beyotime, Jiangsu, China, Cat. No: C2055) for 1 h, followed by nuclear staining with DAPI. Fluorescence was visualized and captured using a fluorescence microscope at excitation/emission wavelengths of 500/510 nm. The relative fluorescent area was quantified and statistically analyzed with ImageJ software.

### 4.17 FITC‐Labeling of rhLF and Cellular Uptake Assay

rhLF was labeled with FITC (Yeasen, Shanghai, China, Cat. No: 60514ES60) according to the manufacturer's instructions. Briefly, FITC was dissolved in DMSO at 1 mg mL^−1^, and rhLF was prepared at 2 mg mL^−1^ in 0.1 m sodium bicarbonate buffer (pH 9.0). 1mL rhLF solution was mixed with 50 µL of FITC solution under light‐protected conditions and incubated at 4°C for 8 h. The reaction was quenched by adding ammonium chloride to a final concentration of 50 mm, followed by incubation at 4°C for 2 h. Xylene cyanol and glycerol were then added to final concentrations of 0.1% and 5%, respectively.

Unconjugated FITC was removed by gel filtration chromatography with a separation range of 4000–120 000 Da. The purified rhLF‐FITC conjugate was collected, stored at 4°C in the dark, and quantified using a BCA assay kit (Lablead, Beijing, China, Cat. No: B5001). For cellular uptake studies, cells were treated with 100 µg mL^−1^ rhLF‐FITC for 48 h, followed by staining of nuclei with DAPI and actin cytoskeleton with phalloidin. Images were acquired using a fluorescence microscope.

### 4.18 CSK Overexpression

The CSK overexpression lentiviral plasmid (pLV3‐CMV‐Csk(mouse)‐CopGFP‐Puro, P83536) and the corresponding empty vector control (pLV3‐CMV‐MCS2‐EF1a‐CopGFP‐Puro, P39198), psPAX2 (DB00001), and pMD2.G (DB00002) were obtained from MiaoLingBio, China. HEK293T cells (Abcell, Beijing, China, Cat. No: AC101) were cultured in high‐glucose DMEM complete medium.

For lentivirus production, HEK293T cells were seeded at 5 × 10^6^ cells per 10 cm dish and transfected at 70%–80% confluence using Lipo8000 (Beyotime, Jiangsu, China, Cat. No: C0533) with a three‐plasmid system containing 10 µg of CSK (or control) plasmid, 7.5 µg of psPAX2, and 5 µg of pMD2.G. After 6–8 h, the medium was replaced. Viral supernatants were collected at 48 and 72 h post‐transfection and filtered through a 0.45 µm membrane.

For CSK overexpression (OE) in primary SVF cells, cells were infected 24 h after plating using a 1:1 mixture of viral supernatant and complete DMEM containing 5 µg mL^−1^ polybrene (Beyotime, Jiangsu, China, Cat. No: C0351). The control group (NC) was infected with the empty vector virus. Fresh complete medium was replaced 24 h post‐infection.

### 4.19 PRMT5 Knockdown by siRNA

Transient knockdown of PRMT5 was achieved using siRNA targeting mouse *Prmt5* (si‐Prmt5) (Hippo Bio, Huzhou, China, Cat. No: 27374), with a non‐targeting siRNA serving as the negative control (siNC). Transfection was performed at 50 nm siRNA working concentration using transfection reagent (D‐Nano Therapeutics, Beijing, China, Cat. No: DN0001) according to the manufacturer's protocol.

### 4.20 Mass Spectrometric Analysis

Proliferating or early‐differentiating SVF cells incubated with rhLF for 48 h were lysed, and immunoprecipitation was performed using an anti‐LF antibody, with IgG as a control. Beads bound to immunocomplexes were collected and submitted to Shanghai bioprofile Co., Ltd (Shanghai, China) for subsequent analysis.

After digestion with sequencing‐grade trypsin, peptides were analyzed by liquid chromatography‐tandem MS (LC‐MS/MS) using an Orbitrap Astral mass spectrometer (Thermo Scientific) coupled to a Vanquish Neo UHPLC system (Thermo Scientific). Full MS scans (m/z 380–980) were acquired at a resolution of 240 000 with an AGC target of 500% and an injection time of 5 ms. Data‐independent acquisition (DIA) MS/MS scans (m/z 150–2000) were performed on the Astral mass analyzer using an isolation window of 2 m/z, an AGC target of 500%, and an injection time of 3 ms. The normalized collision energy was set to 25%, and the cycle time was 0.6 s. Spectra for full MS and DIA MS/MS were recorded in profile and centroid modes, respectively. The DIA‐MS data were processed using DIA‐NN (v1.8.1).

### 4.21 Protein Degradation Assay

To assess CSK protein degradation, proliferating SVF cells were treated with 10 µg mL^−1^ rhLF in combination with one of the following regimens: 5 µg mL^−1^ cycloheximide (CHX) (Abmole, Houston, USA, Cat. No: M4879) alone, 5 µg mL^−1^ CHX plus 5 µm MG132 (Abmole, Houston, USA, Cat. No: M1902), or 5 µg mL^−1^ CHX plus 10 µm chloroquine (CQ) (Abmole, Houston, USA, Cat. No: M9559). Cells were harvested at 0, 2, 4, 6, and 12 h post‐treatment, and CSK levels were analyzed by western blotting. Controls received the same treatments without rhLF.

To examine PRMT5 degradation, early‐differentiating SVF cells were exposed to 10 µg mL^−1^ rhLF along with one of the following: 10 µg mL^−1^ CHX alone, 10 µg mL^−1^ CHX plus 5 µm MG132, or 10 µg mL^−1^ CHX plus 10 µm CQ. Samples were collected at 0, 2, 4, 6, and 12 h, and PRMT5 expression was detected by western blot. Corresponding controls were included without rhLF.

### 4.22 Protein Ubiquitination Assay

To assess the ubiquitination levels of CSK and PRMT5, HEK293T cells were co‐transfected with the following plasmids: For CSK ubiquitination, pCMV‐Csk(mouse)‐EGFP‐3×FLAG‐Neo (MiaoLingBio, Shanghai, China, Cat. No: P78475) and pCMV‐Ub(mouse)‐3×HA‐Puro (MiaoLingBio, Shanghai, China, P73084); For PRMT5 ubiquitination, pCMV‐Prmt5(mouse)‐EGFP‐Neo (MiaoLingBio, Shanghai, China, P66118) and pCMV‐Ub(mouse)‐3×HA‐Puro.

After 24 h of rhLF treatment, cells were exposed to MG132 for 6 h before harvesting. Cell lysates were subjected to immunoprecipitation using anti‐CSK or anti‐PRMT5 antibodies, followed by immunoblotting to detect ubiquitinated forms of the respective proteins.

### 4.23 Molecular Docking

Rigid‐body docking was performed using the GRAMM platform. Briefly, protein structures were retrieved from the UniProtKB database. The two input structures were submitted to the GRAMM system, and protein–protein docking was carried out using default parameters. The top 10 docking results were collected for further evaluation. The optimal docking pose was selected based on a binding energy threshold of < ‐4 kcal mol^−1^, together with analysis of the protein–protein interaction interface area, hydrogen bonding, and key amino acid residues. The binding free energy was calculated using PDBePISA, and the final complex structure was visualized with PyMOL 3.1.

### 4.24 Statistical Analysis

All data were analyzed in SPSS software and are expressed as the mean ± SEM. Groups were compared using Student's two‐tailed unpaired *t*‐test, one‐way or two‐way ANOVA followed by Tukey's test as a post‐hoctest. A *p* value of <0.05 was considered statistically significant. GraphPad Prism 8.0 (GraphPad Software, San Diego, CA, USA) was used for statistical graphing, and Adobe Illustrator 2021 (Adobe Inc., San Jose, CA, USA) was used for figure composition.

## Author Contributions

The study was conceptualized by Q.A. and Y.Z. (Yali Zhang). Q.A. was responsible for the methodology. The investigation was carried out by Q.A., Y.Z. (Yunxia Zou), W.W., Z.C., Z.Z., R.L., and X.W. Q.A. drafted the original manuscript, while Q.A., Y.Z. (Yunxia Zou), and Y.Z. (Yali Zhang) reviewed and edited the manuscript. Funding acquisition was handled by Q.M. and Y.Z. (Yali Zhang). Resources were provided by K.H., F.D., Y.D., and Q.M. The study was supervised by Y.Z. (Yali Zhang).

## Funding

This work was supported by grants from the China Agriculture Research System of MOF and MARA (CARS‐36), Biological Breeding‐Major Projects in National Science and Technology (2023ZD0404905).

## Conflicts of Interest

The authors declare no conflicts of interest.

## Supporting information




**Supporting File 1**: advs75678‐sup‐0001‐SuppMat.docx.

**Supporting File 2**: advs75678‐sup‐0002‐SuppMat.doc.

**Supporting File 3**: advs75678‐sup‐0003‐TableS1.xlsx.

## Data Availability

All data are available in the main text or the supplementary materials. The RNA‐seq and snRNA‐seq data have been deposited into China National GeneBank (CNGB) with accession number CNP0008487 and CNP0008488.
