# Supplementary material for: Lactoferrin Deficiency During Lactation Causes Adult Obesity‐Related Metabolic Disease Through Persistent Adipose Dysfunction Driven by Impaired Adipocyte Development
Source: Adv Sci (Weinh). 2026 May 19:e75678. Online ahead of print. doi: 10.1002/advs.75678 (PMC13335935; doi:10.1002/advs.75678)
Supplement: Supplementary file 1 — Supporting File 1: advs75678‐sup‐0001‐SuppMat.docx. [file ADVS-9999-e75678-s003.docx]

Supporting Information

**Lactoferrin Deficiency during Lactation causes Adult Obesity-Related Metabolic Disease through Adipose Dysfunction Driven by Impaired Adipocyte Development**

*Qin* *An*, *Yunxia* *Zou*, *Wenli* *Wang*, *Zhimei* *Cheng*, *Zhuoxing* *Zhang*, *Ruwei* *Liu*, *Xiong* *Wang*, *Kunlun* *Huang*, *Fangrong* *Ding*, *Yunping* *Dai*, *Qingyong* *Meng*, *Yali* *Zhang* ^*^


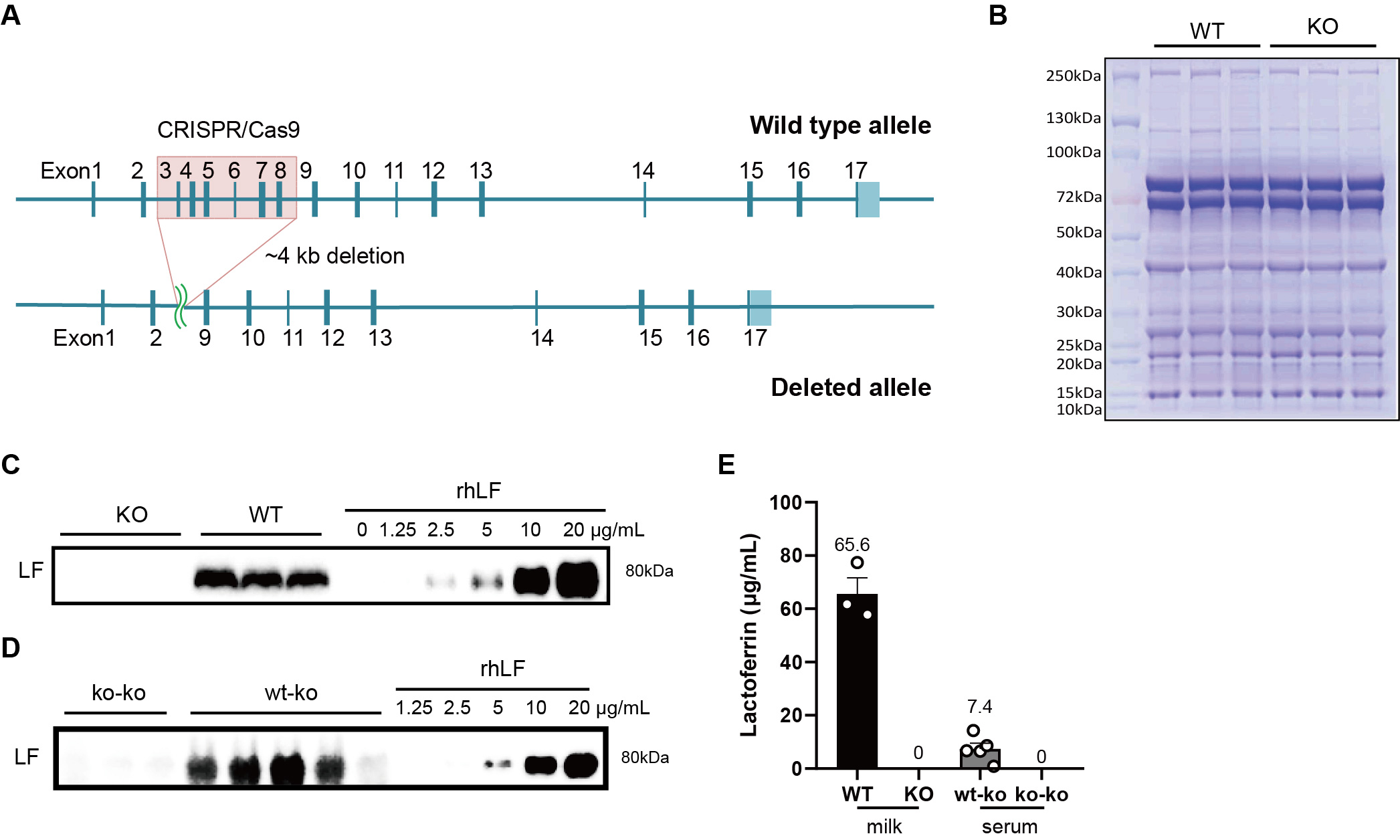


**Figure S1.** **Construction of lactoferrin gene knockout mice.** (A) The construction strategy of lactoferrin (LF) gene knockout mice: Deletion of exons 3 to 8 of the *Lf* gene in C57BL/6N mice using CRISPR-Cas9 technology, resulting in an approximately 4 kb genomic fragment deletion, to construct a systemic *Lf* gene knockout model. (B) Coomassie Brilliant Blue staining for detecting protein expression in mouse milk. WT, wild type mice; KO, *Lf* knockout mice, n = 3. (C) Western blot analysis of LF in mouse milk. Milk samples (n = 3) were diluted 8-fold for detection, with 0-20 μg mL^-1^ recombinant human LF (rhLF) used as a standard. (D) Western blot analysis of LF in ko-ko (KO dams nursing KO pups) or wt-ko (WT dams nursing KO pups) mice serum (n = 3/ko-ko group; n = 5/wt-ko group). 1.25-20 μg mL^-1^ rhLF used as a standard. (E) Milk and serum LF levels shown in C, D. Data are mean ± SEM.


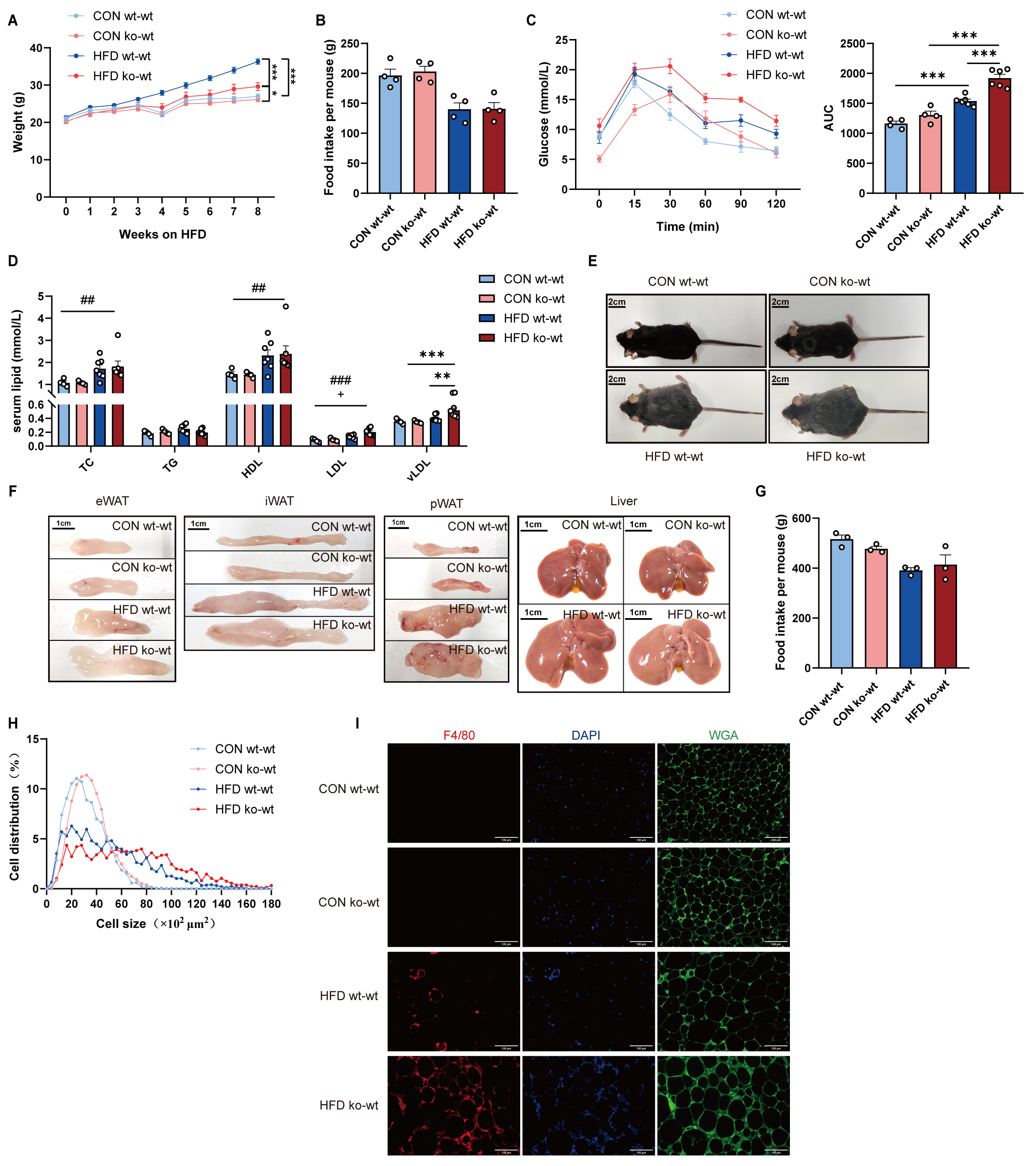


**Figure S2.** **Effects of LF Deficiency During the Lactation Period on Basic Phenotypes of HFD feeding mice.** (A) Weekly body weights of mice during the 8 weeks HFD feeding period (n = 4/CON group; n = 7/HFD group). (B) Total food intake of mice during 8 weeks of HFD feeding (n = 4). (C) Glucose tolerance tests (GTT) and Area under curve (AUC) measurements of GTT after 7 weeks of HFD feeding (n = 4/CON group; n = 7/HFD group). (D) Serum TC、TG、HDL、LDL and vLDL levels after 8 weeks of HFD feeding (n = 4/CON group; n = 7/HFD group). (E) The representative mice size at the end of experiments. Scale bar = 2 cm. (F) The representative organ size at the end of experiments. Scale bar = 1 cm. (G) Total food intake of mice during 20 weeks of HFD feeding (n = 3). (H) Adipocyte size distribution in eWAT of mice after 20 weeks of HFD feeding (n = 7). (I) Representative 100x immunofluorescence images of F4/80 (red) expression in eWAT from 20 weeks HFD feeding mice. Cytomembrane were labeled by WGA（Green）；Nuclei were labeled by DAPI (blue). Scale bar = 130 μm. Data are mean ± SEM. Significance was calculated using two-way ANOVA followed by Tukey’s test. ****P* < 0.001, ***P* < 0.01 and **P* < 0.05 indicate significant differences between groups; ^###^*P* < 0.001, ^##^*P* < 0.01 indicate significant main effects of diet; ^+^*P* < 0.05 indicates a significant main effect of lactational exogenous LF.


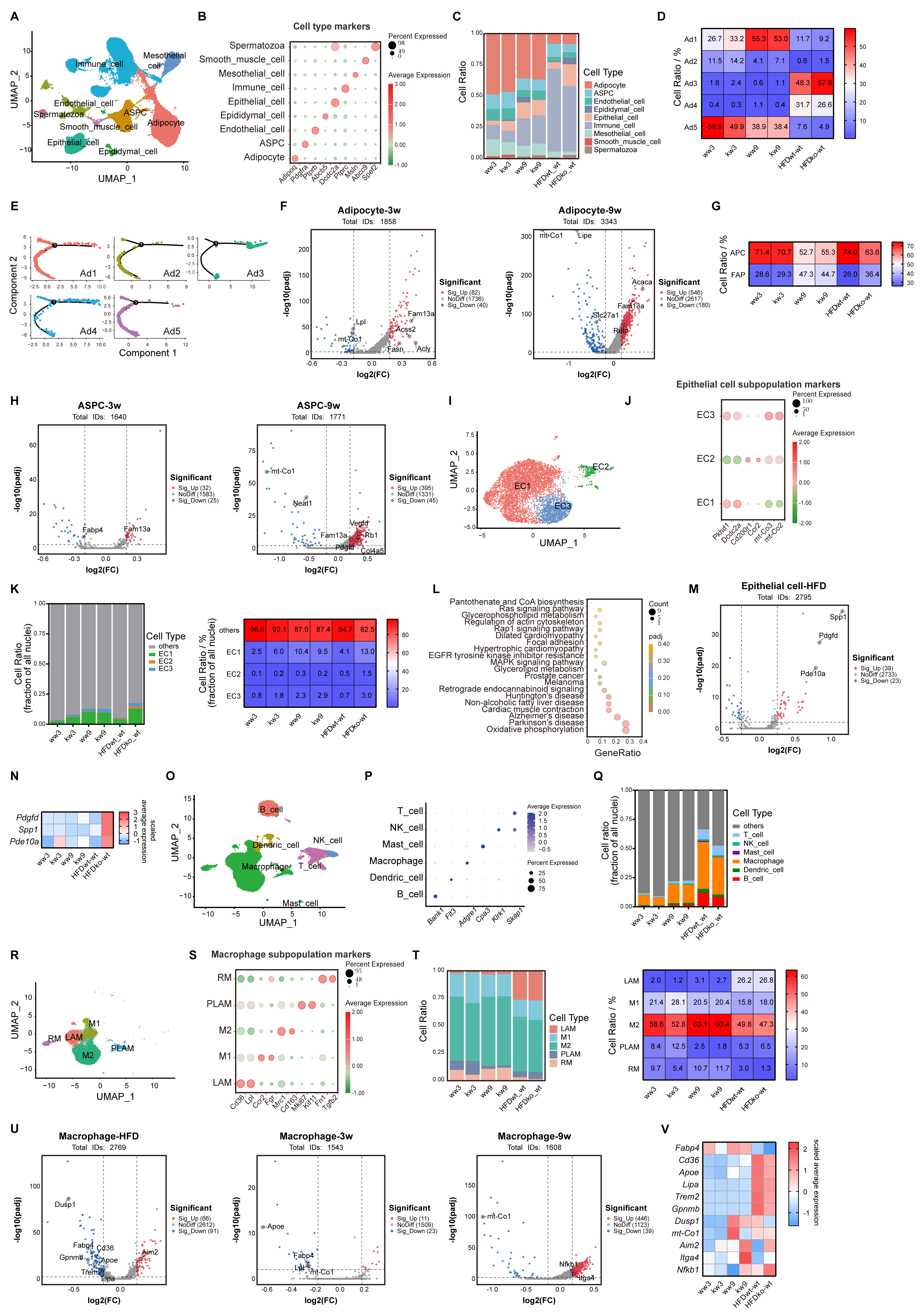


**Figure S3.** **Lactational LF Deficiency alters epithelial cell and macrophage subpopulation composition and impairs their homeostatic function.** (A) UMAP of eWAT cell types. (B) Dotplot showing scaled average expression of selected cell-type-enriched marker genes: Spermatoza (*Spef2*), Smooth muscle cell (*Abcc9*), Mesothelial cell (*Msln*), Immune cell (*Ptprc*), Epithelial cell (*Dcdc2a*), Epididymal cell (*Abcb5*), Endothelial cell (*Ptprb*), ASPC (*Pdgfra*), and Adipocyte (*Adipoq*). (C) The average fraction (relative to the total number of nuclei) of each cell type. (D) The average fraction (relative to the total number of adipocyte nuclei) of Adipocytes subpopulation. (E) Pseudo-time cell trajectory of Ad1, Ad2, Ad3, Ad4 and Ad5 subpopulation. (F) Volcano Plot showing DEGs (|log2FC| > 0.18, padj < 0.01) of adipocyte subpopulation between ko-wt group and wt-wt group of 3w mice, 9w mice. (G) The average fraction (relative to the total number of ASPC nuclei) of APC and FAP subpopulation. (H) Volcano Plot showing DEGs (|log2FC| > 0.2, padj < 0.01) of ASPC subpopulation between ko-wt group and wt-wt group of 3w mice, 9w mice. (I) UMAP of epithelial cell subpopulations. (J) Dotplot showing scaled average expression of epithelial cell subpopulation marker genes: EC1 (*Pkhd1*, *Dcdc2a*), EC2 (*Cd200r1*, *Ccr2*), EC3 (*mt-Co3*, *mt-Co2*). (K) The average fraction (relative to the total number of nuclei) of epithelial cell subpopulation. (L) KEGG enrichment analysis of DEGs (|log2FC| > 0.25, padj < 0.01) in epithelial cells between HFD wt-wt group and HFD ko-wt group mice. (M) Volcano Plot showing DEGs of epithelial cells subpopulation between HFD wt-wt group and HFD ko-wt group mice. (N) Heatmap showing DEGs in epithelial cells between ko-wt group and wt-wt group. (O) UMAP of immune cells subpopulations. (P) Dotplot showing scaled average expression of immune cells subpopulation marker genes: T cell (*Skap1*), natural killer (NK) cell (*Klrk1*), Mast cell (*Cpa3*), Macrophage (*Adgre1*), Dendric cell (*Flt3*), and B cell (*Bank1*). (Q) The average fraction (relative to the total number of nuclei) of each subpopulation. (R) UMAP of macrophage subpopulations. (S) Dotplot showing scaled average expression of macrophage subpopulation marker genes: RM (*Fn1*, *Tgfb2*), PLAM (*Mki67*, *Kif11*), M2 (*Mrc1*, *Cd163*), M1 (*Ccr2*, *Fgr*), and LAM (*Cd36*, *Lpl*). (T) The average fraction (relative to the total number of macrophage nuclei) of each subpopulation. (U) Volcano Plot showing DEGs (|log2FC| > 0.18, padj < 0.01) of macrophage subpopulation between ko-wt group and wt-wt group of 20 weeks HFD feeding mice, 3w mice, and 9w mice. (V) Heatmap showing DEGs in macrophages between ko-wt group and wt-wt group. snRNA-seq data visualizations were generated using NovoMagic (magic.novogene.com).


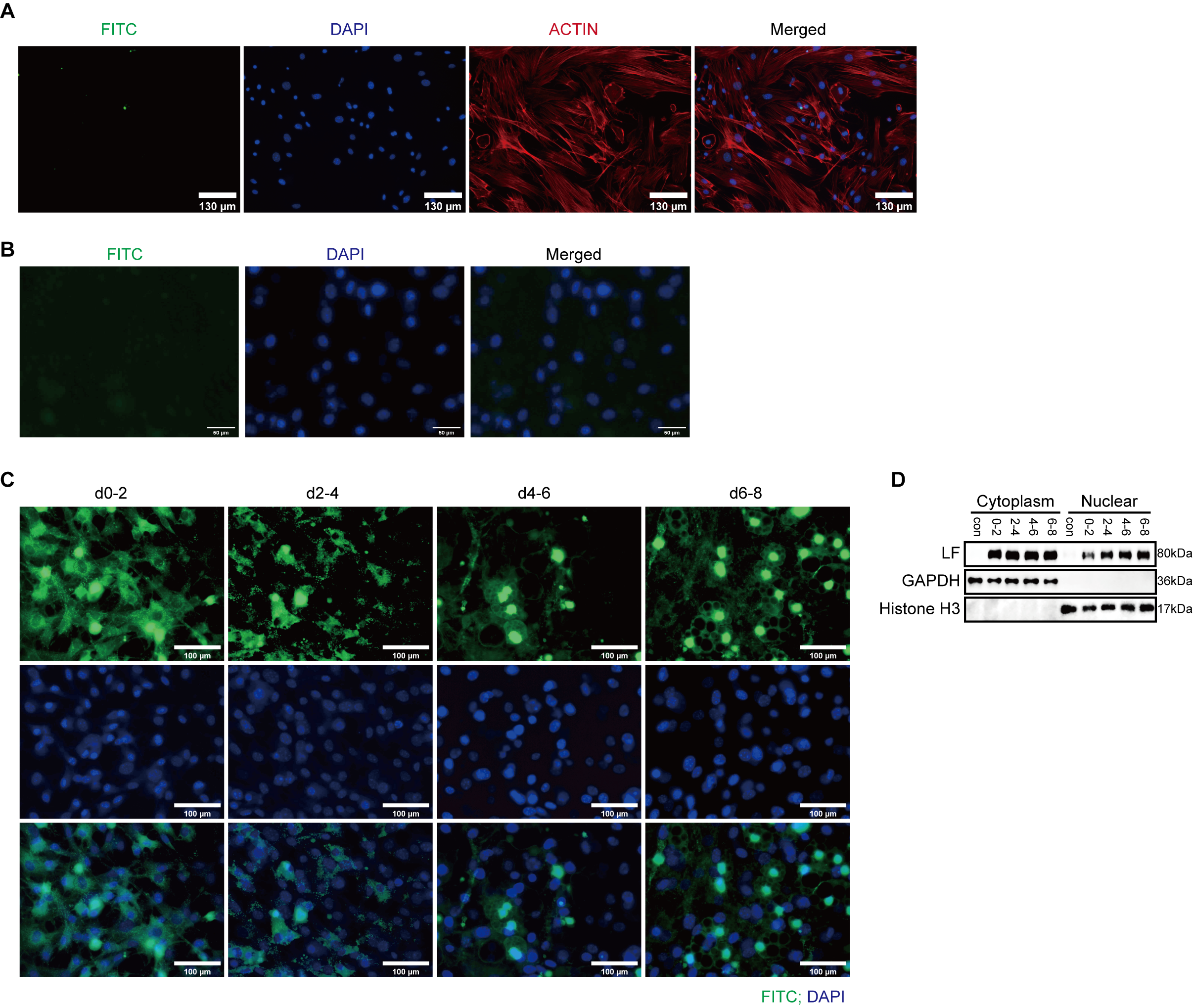


**Figure S4. Localization of exogenous rhLF in SVF cells derived from eWAT.** (A) Representative 100x immunofluorescence images of FITC (green) location in SVF cells after 48 h of FITC treatment. ACTIN were labeled by Phalloidin（Red）；Nuclei were labeled by DAPI (blue). Scale bar = 130 μm. (B) Representative 400x immunofluorescence images of FITC (green) location in SVF cells after 8 days of adipogenic differentiation and FITC treatment. Nuclei were labeled by DAPI (blue). Scale bar = 50 μm. (C) Representative 400x immunofluorescence images of rhLF (green) location in SVF cells with FITC-rhLF during 0-2 days, 2-4 days, 4-6 days, and 6-8 days of adipogenic differentiation. Nuclei were labeled by DAPI (blue). Scale bar = 100 μm. (D) Immunoblotting of rhLF distribution in SVF cells with 10 μg mL^-1^ rhLF during 0-2 days, 2-4 days, 4-6 days, and 6-8 days of adipogenic differentiation.


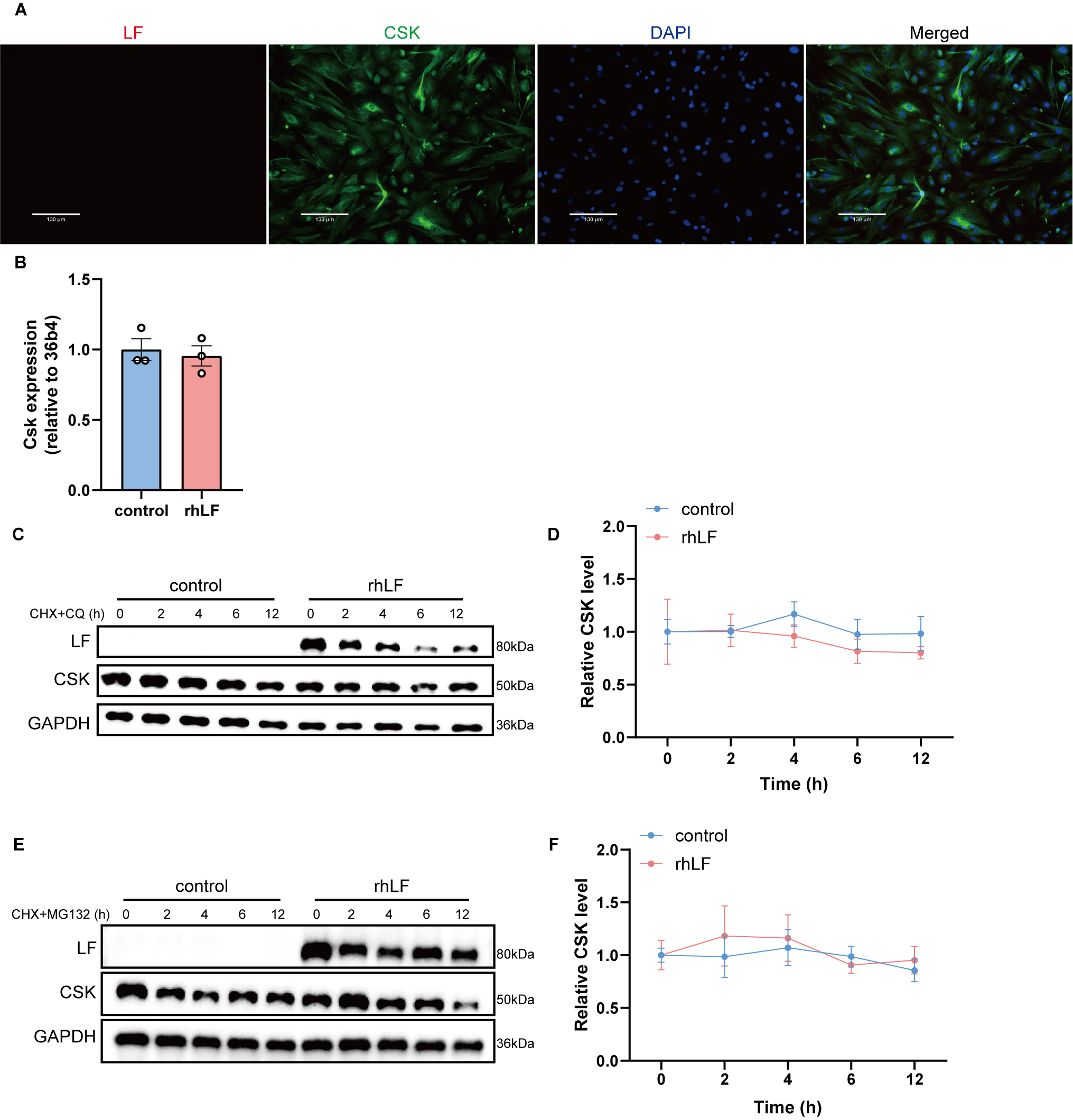


**Figure S5.** **rhLF affects the stability of the CSK protein.** (A) Representative 100x immunofluorescence images of rhLF (red) and CSK (green) location in SVF cells after 48 h of control treatment. Nuclei were labeled by DAPI (blue). Scale bar = 130 μm. (B) Relative mRNA levels of csk in SVF cells with 10 μg mL^-1^ rhLF treated for 48 h (n = 3). (C, D) Western blot and quantification of CSK in 10 μg mL^-1^ CHX-treated and 20 mμ CQ-treated SVF cells with 10 μg mL^-1^ rhLF (n = 4). (E, F) Western blot and quantification of CSK in 10 μg mL^-1^ CHX-treated and 5 μg mL^-1^ MG132-treated SVF cells with 10 μg mL^-1^ rhLF (n = 3). The group treated with 0 μg mL^-1^ rhLF served as the control. Data are mean ± SEM. Significance for was calculated using Student’s two-tailed unpaired t-test.


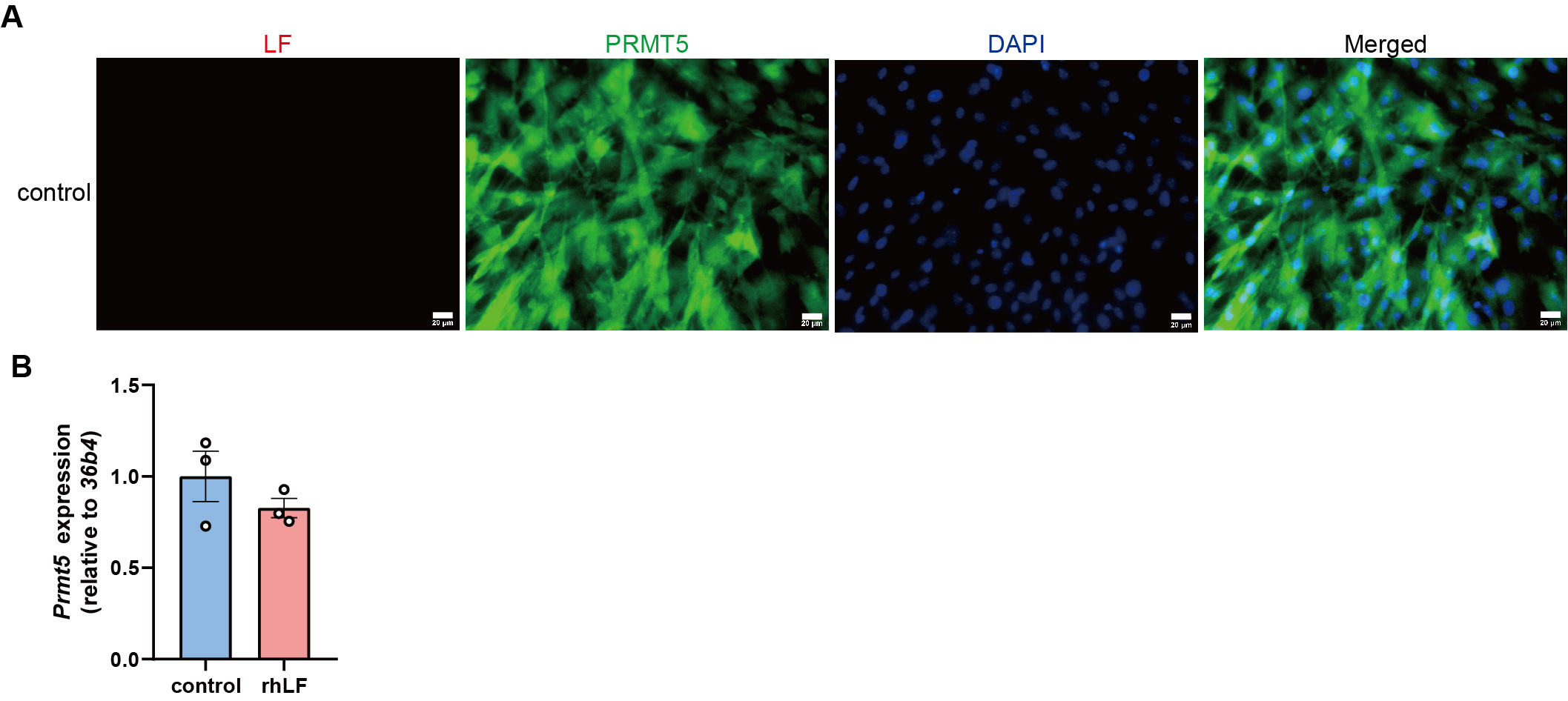


**Figure S6.** **rhLF does not affect the gene expression of *Prmt5.*** (A) Representative 100x immunofluorescence images of rhLF (red) and PRMT5 (green) location in SVF cells after 48 h of control treatment. Nuclei were labeled by DAPI (blue). Scale bar = 20 μm. (B) Relative mRNA levels of *Prmt5* in SVF cells at the early stage of differentiation with 10 μg mL^-1^ rhLF (n=3). The group treated with 0 μg mL^-1^ rhLF served as the control. Data are mean ± SEM. Significance for was calculated using Student’s two-tailed unpaired t-test. **Table S1. Primer sequences used for quantitative real-time PCR.**

| Gene | Forward primer sequence (5' to 3') | Reverse primer sequence (5' to 3') | Species |
| --- | --- | --- | --- |
| *36b4* | CGTCCTGGCATTGTCTGTG | TGATTCCTCCGACTCTTCCTT | Mouse |
| *Lpl* | TCGCCTTTCTCCTGATGACG | GCAATCACACGGATGGCTTC | Mouse |
| *Fabp4* | ATTTGGTCACCATCCGGTCA | CATAACACATTCCACCACCAGC | Mouse |
| *Cd36* | GGAGGCATTCTCATGCCAGT | CTGCTGTTCTTTGCCACGTC | Mouse |
| *Adipoq* | ATCGCTCAGCGTTCAGTGTG | TCGTAGGTGAAGAGAACGGC | Mouse |
| *Retn* | TCGATGAAGCCATCGACAAGA | GAATGTCCCACGAGCCACAG | Mouse |
| *Lep* | ATTTCACACACGCAGTCGGT | ACATTTTGGGAAGGCAGGCT | Mouse |
| *Pparg* | ATTGAGTGCCGAGTCTGTGG | GGCATTGTGAGACATCCCCA | Mouse |
| *Cebpα* | TAACTCCCCCATGGAGTCGG | TATAGACGTCTCGTGCTCGC | Mouse |
| *Srebp1* | GACACAGCGGTTTTGAACGA | GCTCTCAGGAGAGTTGGCAC | Mouse |
| *Acc* | GGTCAAGTCCTTCCTGCTCACA | TGGTGTAACTGCTGCCGTCATA | Mouse |
| *Fasn* | CCCACCCTGATTTCTGCCAT | CTCCACTCCCGAATGTGCTT | Mouse |
| *Cebpδ* | GACTCCTGCCATGTACGACG | GTTGAAGAGGTCGGCGAAGA | Mouse |
| *Cebpβ* | GACAAGCTGAGCGACGAGTA | TGCTTGAACAAGTTCCGCAG | Mouse |
| *Trem2* | GGAACCGTCACCATCACTCT | GAGGTGACCCACAGGATGAA | Mouse |
| *Gpnmb* | CTACAACTGGACTGCAGGGG | TTTCTTCCATCCATGGGGGC | Mouse |
| *Csk* | ATGCAGCTGGTGGAGCACTA | TTTGTTGCCCCGGTAATCCC | Mouse |
| *Prmt5* | GCCAAGCAGGGGTTTGATTTC | AGCCGCTTCAGAGTTCCTTC | Mouse |
| *Ccna2* | CTTGTAGGCACGGCTGCTAT | AGGTGCAGGAAGTACTGGGT | Mouse |
| *Ccnb1* | ACCAGAGGTGGAACTTGCTG | TCTGCGTCTACGTCACTCAC | Mouse |
| *Ccnd1* | CAAGTGTGACCCGGACTGC | CACATCTCGCACGTCGGT | Mouse |
| *Ccnd2* | GGGAGATTGGGTTCACAGGG | TAGGAACACTGCGGGAGGTA | Mouse |
| *Cdk1* | AAGTGTGGCCAGAAGTCGAG | AAAGTACGGGTGCTTCAGGG | Mouse |
| *Cdk4* | ACTCGATATGAACCCGTGGC | AGCACAGACATCCATCAGCC | Mouse |
| *Cdk6* | GCATCGTGATCTGAAACCGC | CCACGTCTGAACTTCCACGA | Mouse |
| *Mcp-1* | AGATGCAGTTAACGCCCCAC | CCCATTCCTTCTTGGGGTCA | Mouse |
| *Il-6* | CAACGATGATGCACTTGCAGA | TCTGTGACTCCAGCTTATCTCTTG | Mouse |
| *Il-1β* | ATGCCACCTTTTGACAGTGATG | AGCTTCTCCACAGCCACAAT | Mouse |
| *Tnf-α* | CGAGTGACAAGCCTGTAGCC | ACAAGGTACAACCCATCGGC | Mouse |
| *36b4* | GTTGCTGGCCAATAAGGTGC | CAGCTGCACATCACTCAGGA | Human |
| *Lpl* | CAGGATGTGGCCCGGTTTAT | GGGACCCTCTGGTGAATGTG | Human |
| *Fabp4* | AAGTAGGAGTGGGCTTTGCC | TGCACATGTACCAGGACACC | Human |
| *Cd36* | CGCTGAGGACAACACAGTCT | CTGCCACAGCCAGATTGAGA | Human |
| *Adipoq* | TGCAACATTCCTGGGCTGTA | CCATACACCTGGAGCCAGAC | Human |
| *Retn* | GAGTCCACGCTCCTGTGTTCC | ACTGGCAGTGACATGTGGTCTC | Human |
| *Pparg* | ACCCAGAAAGCGATTCCTTCA | CACGGAGCTGATCCCAAAGT | Human |
| *Cebpα* | TAACTCCCCCATGGAGTCGG | ATGTCGATGGACGTCTCGTG | Human |
| *Srebp1* | CATGCGGAGGGTGTTCCTAC | AGCACATCCATCAGCTCCTT | Human |
| *Acc* | TAGTCTGCCACGGATCCAGA | GGGAGGGATCTCTGAGGGTT | Human |
| *Fasn* | TGCGTGGCCTTTGAAATGTG | CTCCATGTCCGTGAACTGCT | Human |
| *Cebpδ* | TCTTCGCCGACCTCTTCAAC | CAAGCTCACCACGGTCTGT | Human |
| *Cebpβ* | AGAAGACCGTGGACAAGCAC | GCTTGAACAAGTTCCGCAGG | Human |
| *Ccna1* | CTTGAGGCGACAAGGAGTGT | TGAAGCTCACTCAGGCAAGG | Human |
| *Ccna2* | CGGTACTGAAGTCCGGGAAC | CATGAATGGTGAACGCAGGC | Human |
| *Ccnb1* | GCACTTCCTTCGGAGAGCAT | TTCTTAGCCAGGTGCTGCAT | Human |
| *Ccnd1* | GATGCCAACCTCCTCAACGA | GGAAGCGGTCCAGGTAGTTC | Human |
| *Ccnd2* | TACCTGGACCGTTTCTTGGC | TCCACTTCAACTTCCCCAGC | Human |
| *Cdk1* | CCCTCCTGGTCAGTACATGG | GCTCTGGCAAGGCCAAAATC | Human |
| *Cdk4* | GCCAGTGGCTGAAATTGGTG | AAAGCCACCTCACGAACTGT | Human |
| *Cdk6* | ACAGAGCACCCGAAGTCTTG | CTGGGAGTCCAATCACGTCC | Human |

(All amplicon lengths are between 100-300 bp).
