# Supplementary material for: Lactoferrin Deficiency During Lactation Causes Adult Obesity‐Related Metabolic Disease Through Persistent Adipose Dysfunction Driven by Impaired Adipocyte Development
Source: Adv Sci (Weinh). 2026 May 19:e75678. Online ahead of print. doi: 10.1002/advs.75678 (PMC13335935; doi:10.1002/advs.75678)
Supplement: Supplementary file 2 — Supporting File 2: advs75678‐sup‐0002‐SuppMat.doc. [file ADVS-9999-e75678-s001.doc]

Supporting Information

**Lactoferrin Deficiency during Lactation causes Adult Obesity-Related Metabolic Disease through Persistent Adipose Dysfunction Driven by Impaired Adipocyte Development**

*Qin* *An*, *Yunxia* *Zou*, *Wenli* *Wang*, *Zhimei* *Cheng*, *Zhuoxing* *Zhang*, *Ruwei* *Liu*, *Xiong* *Wang*, *Kunlun* *Huang*, *Fangrong* *Ding*, *Yunping* *Dai*, *Qingyong* *Meng*, *Yali* *Zhang* ^*^


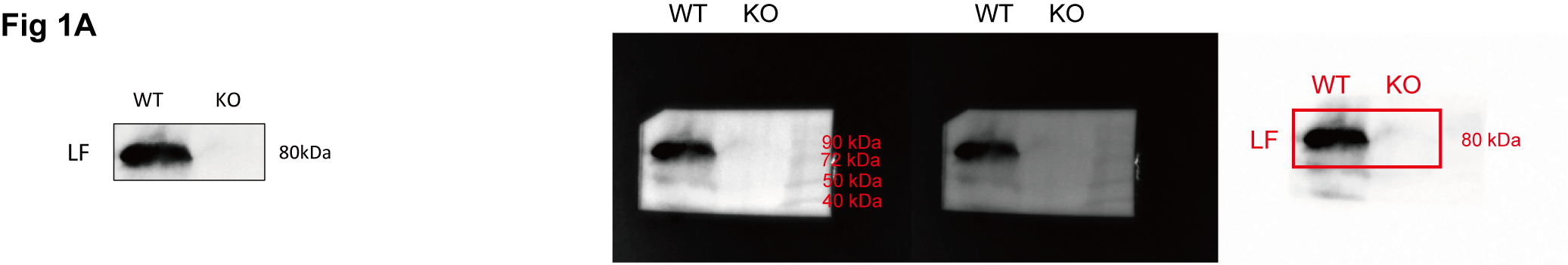


**Supplementary Figure S1**: Original images of blots corresponding to Figure 1A in the main text. Red boxes indicate the cropped areas shown in the main figure.


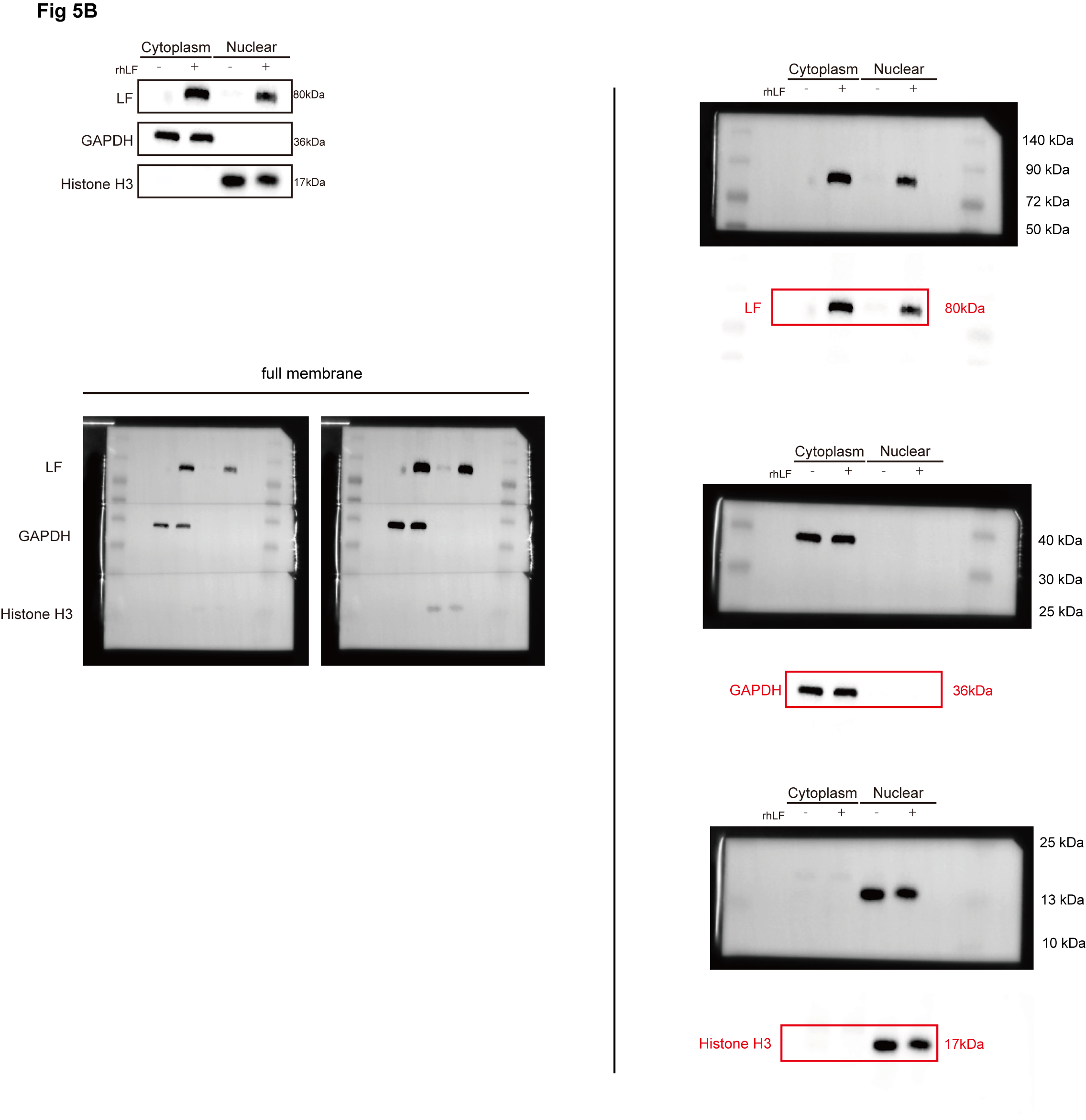


**Supplementary Figure S2**: Original images of blots corresponding to Figure 5B in the main text. Red boxes indicate the cropped areas shown in the main figure.


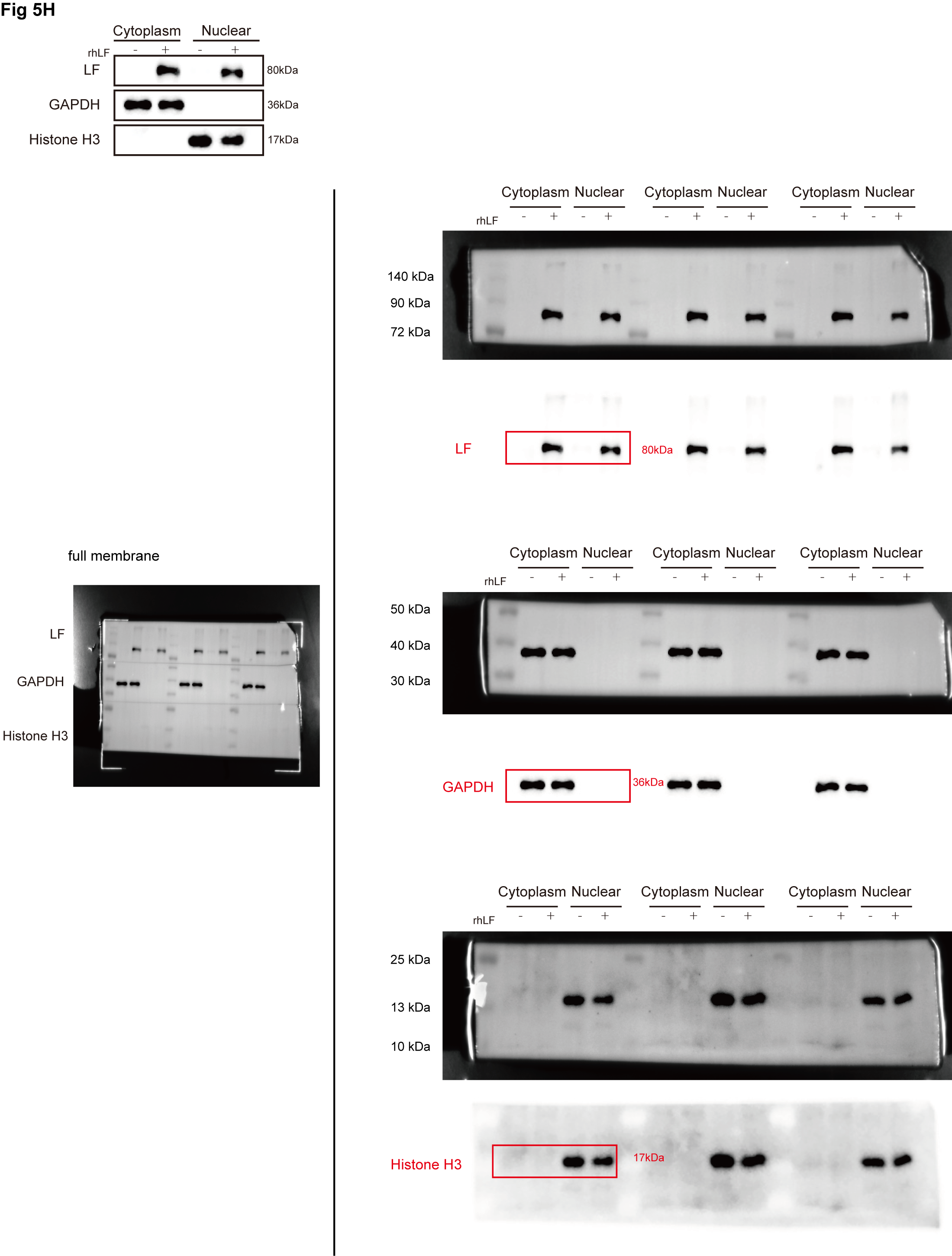


**Supplementary Figure S3**: Original images of blots corresponding to Figure 5H in the main text. Red boxes indicate the cropped areas shown in the main figure.


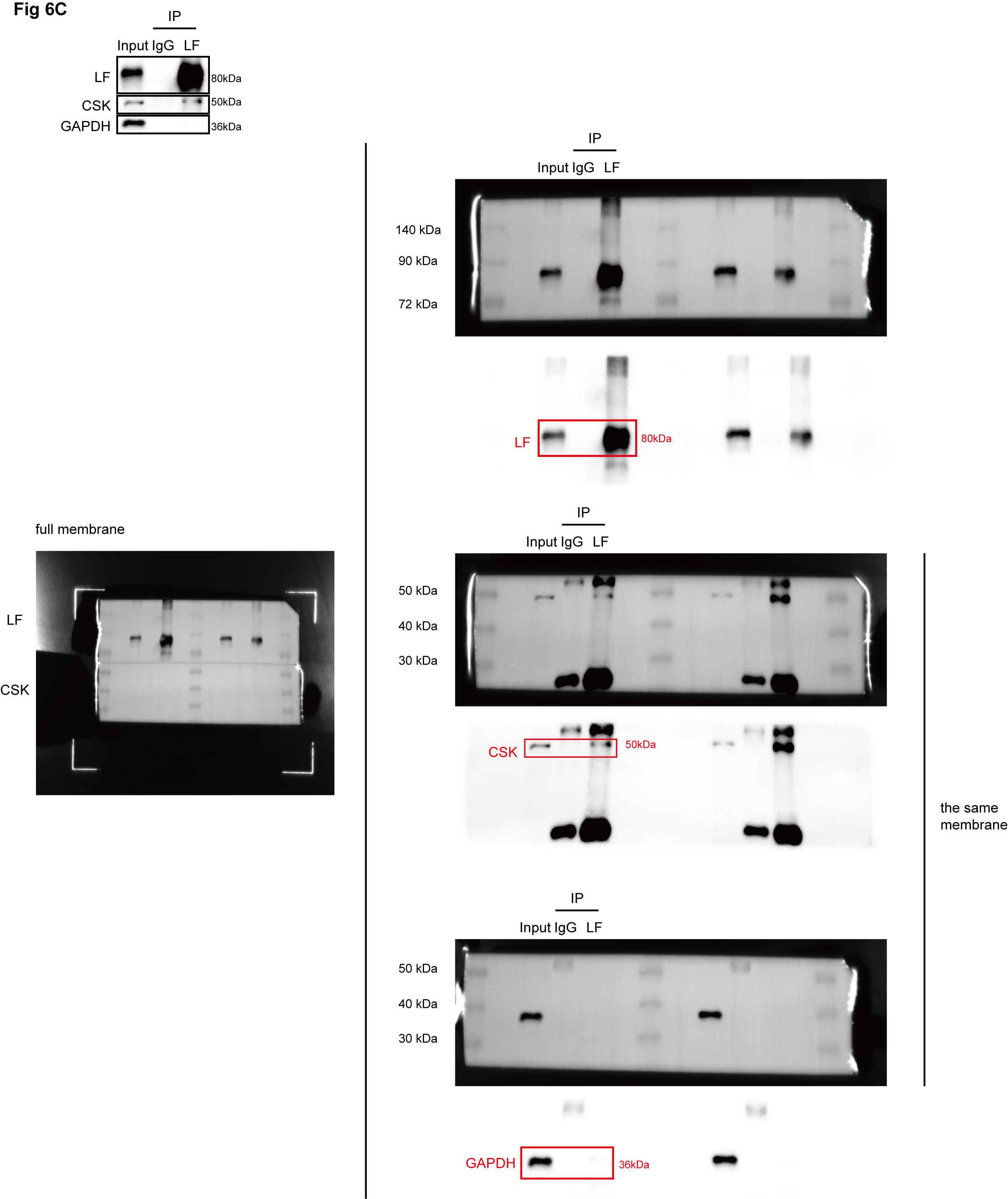


**Supplementary Figure S4:** Original images of blots corresponding to Figure 6C in the main text. Red boxes indicate the cropped areas shown in the main figure.


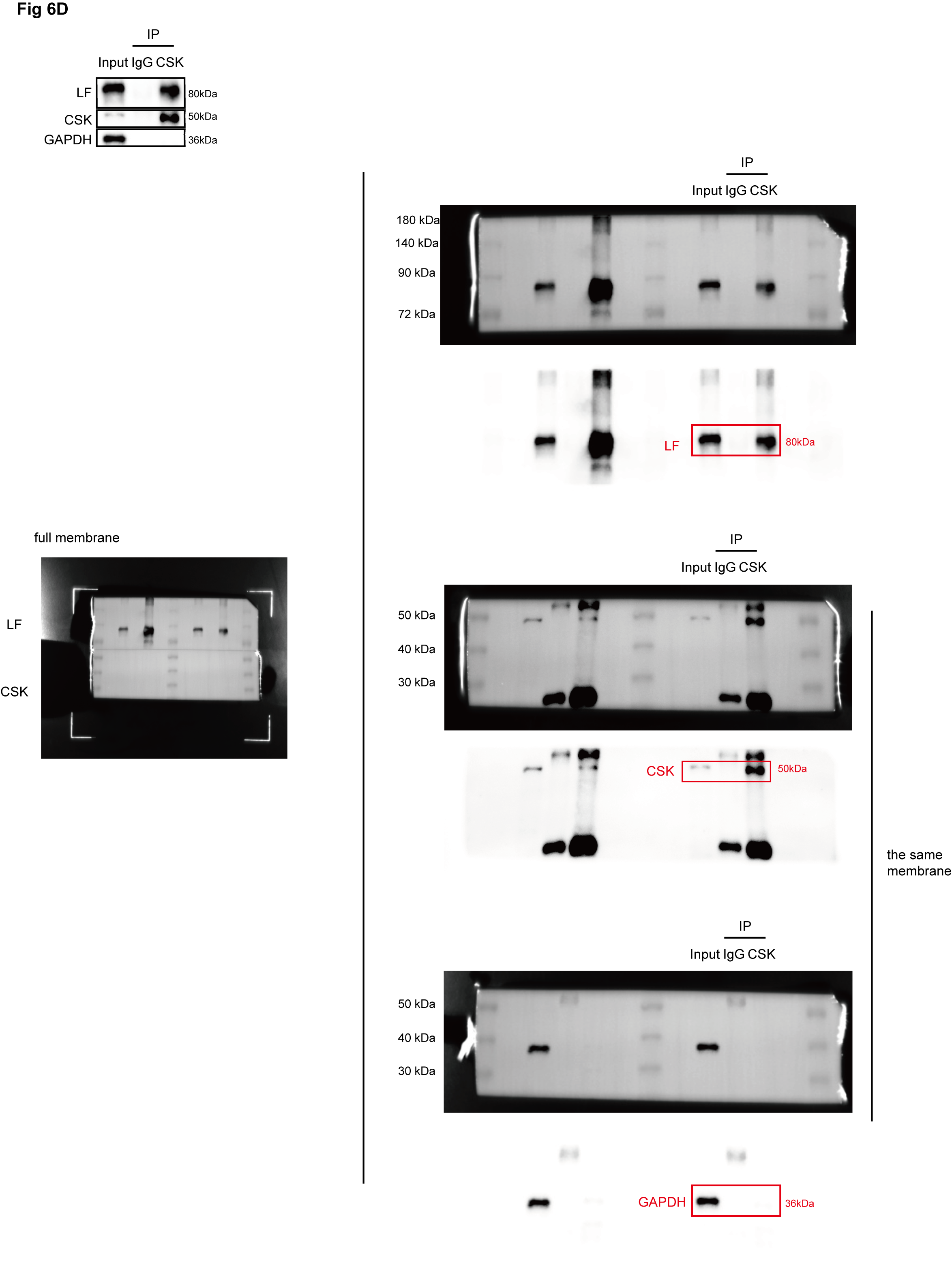


**Supplementary Figure S5**: Original images of blots corresponding to Figure 6D in the main text. Red boxes indicate the cropped areas shown in the main figure.


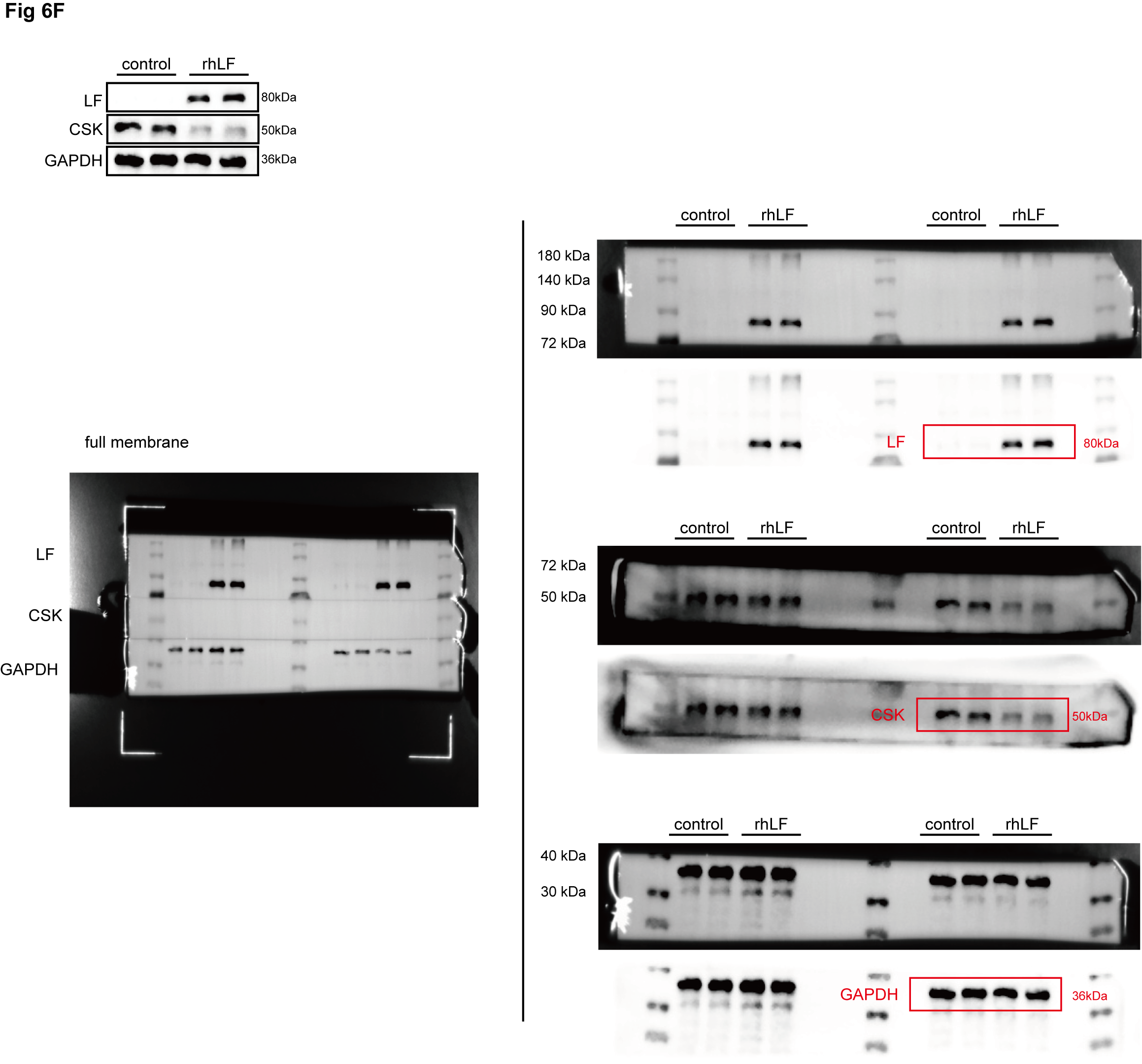


**Supplementary Figure S6**: Original images of blots corresponding to Figure 6F in the main text. Red boxes indicate the cropped areas shown in the main figure.


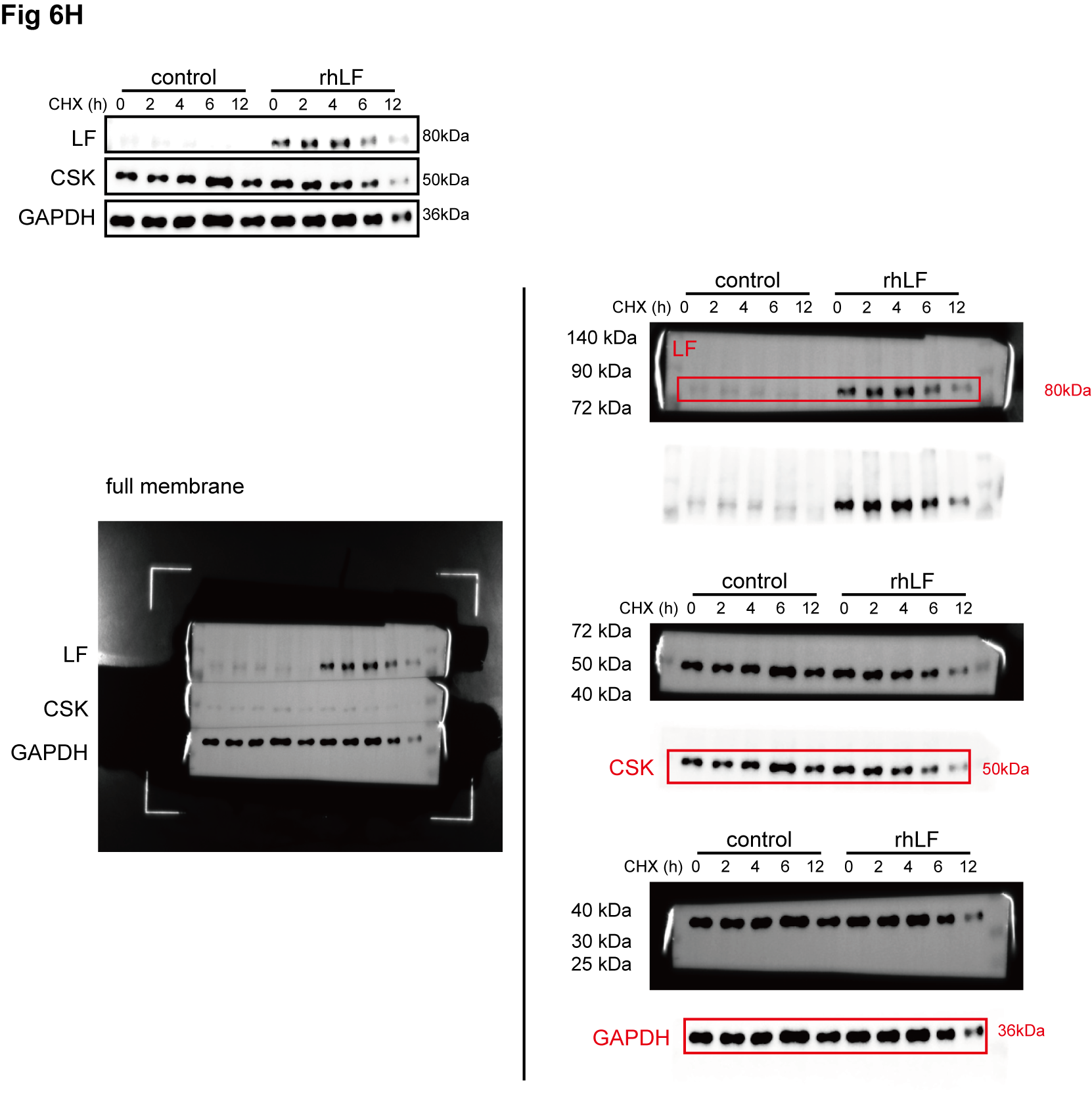


**Supplementary Figure S7**: Original images of blots corresponding to Figure 6H in the main text. Red boxes indicate the cropped areas shown in the main figure.


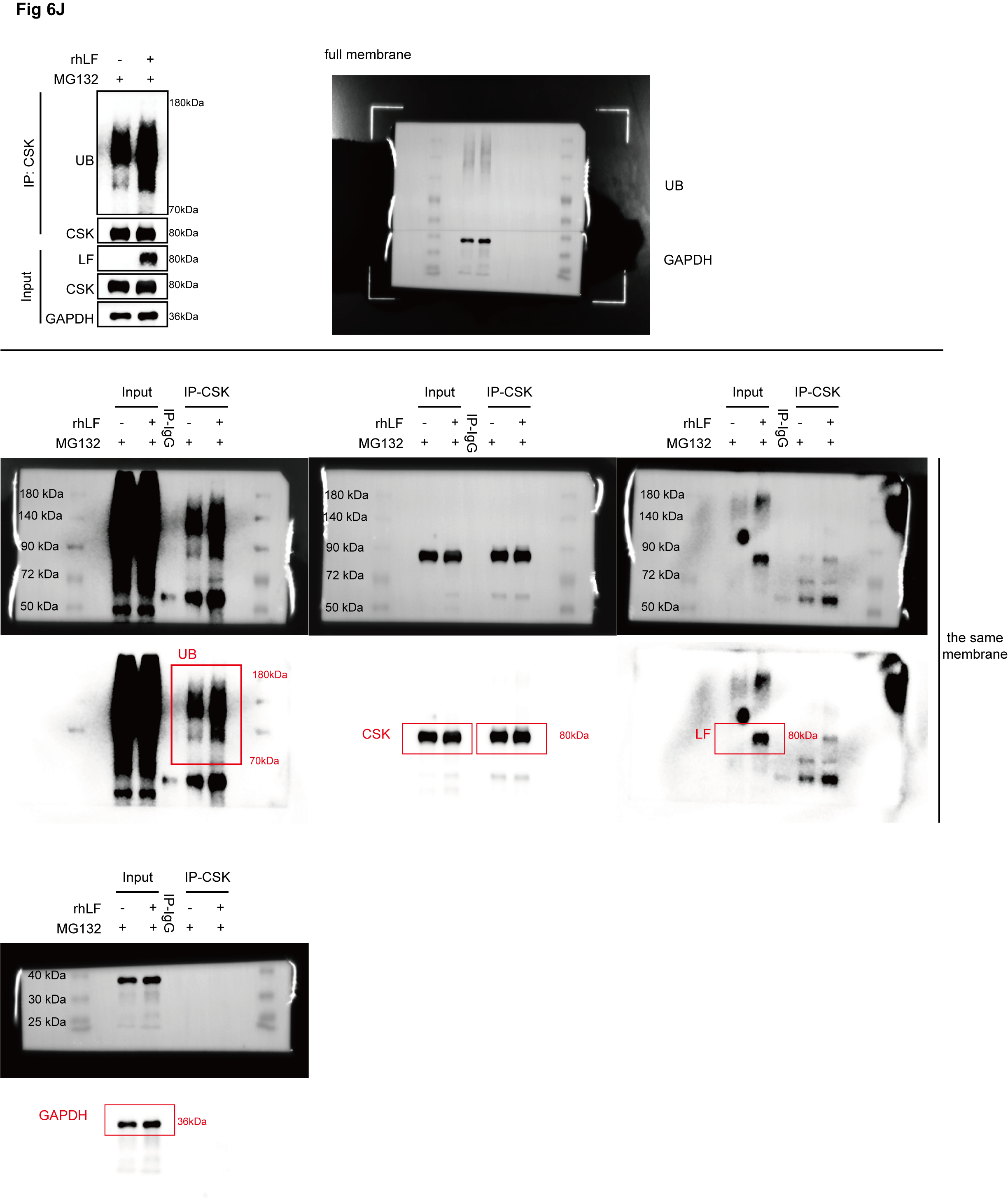


**Supplementary Figure S8**: Original images of blots corresponding to Figure 6J in the main text. Red boxes indicate the cropped areas shown in the main figure.


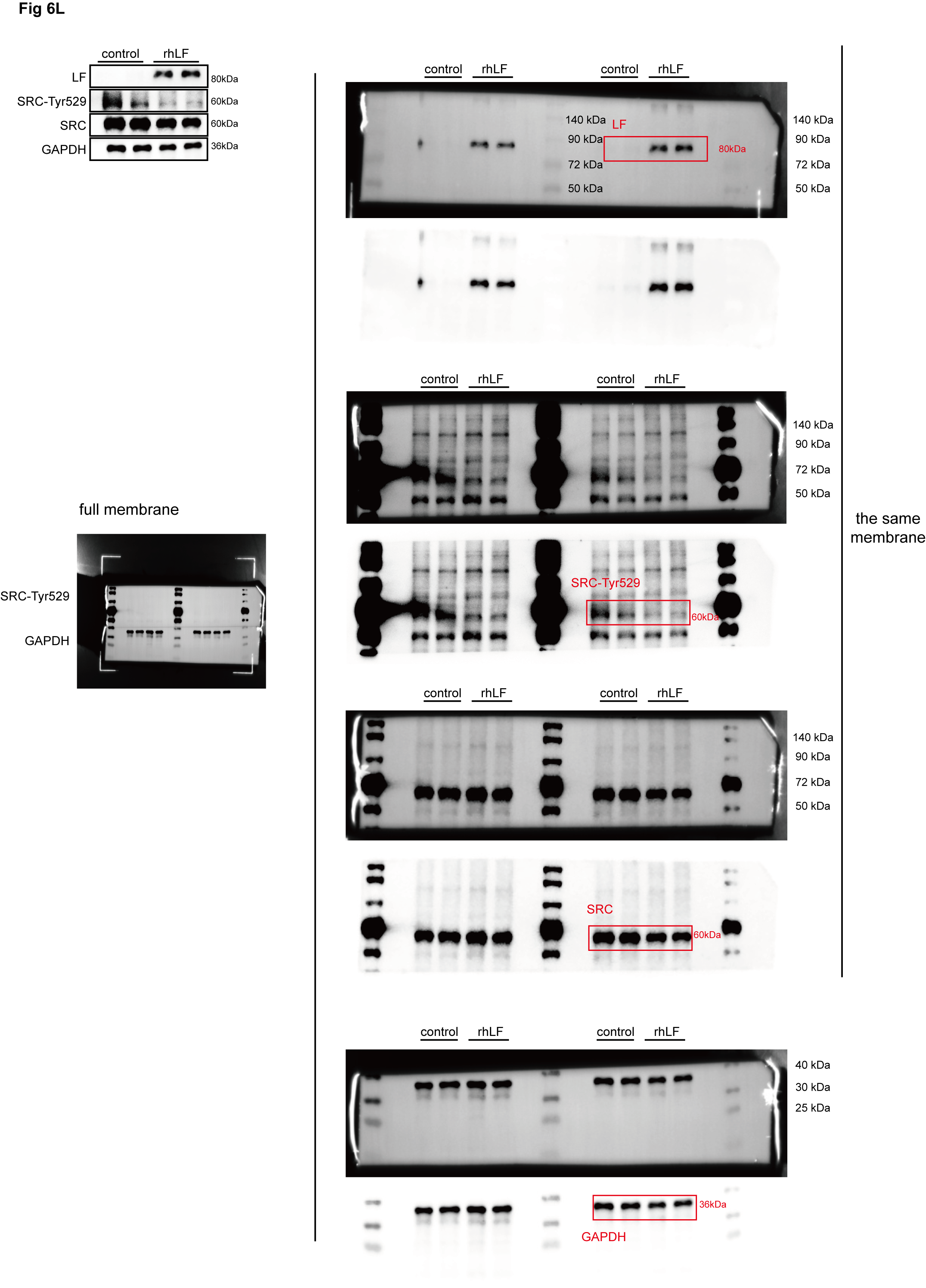


**Supplementary Figure S9**: Original images of blots corresponding to Figure 6L in the main text. Red boxes indicate the cropped areas shown in the main figure.


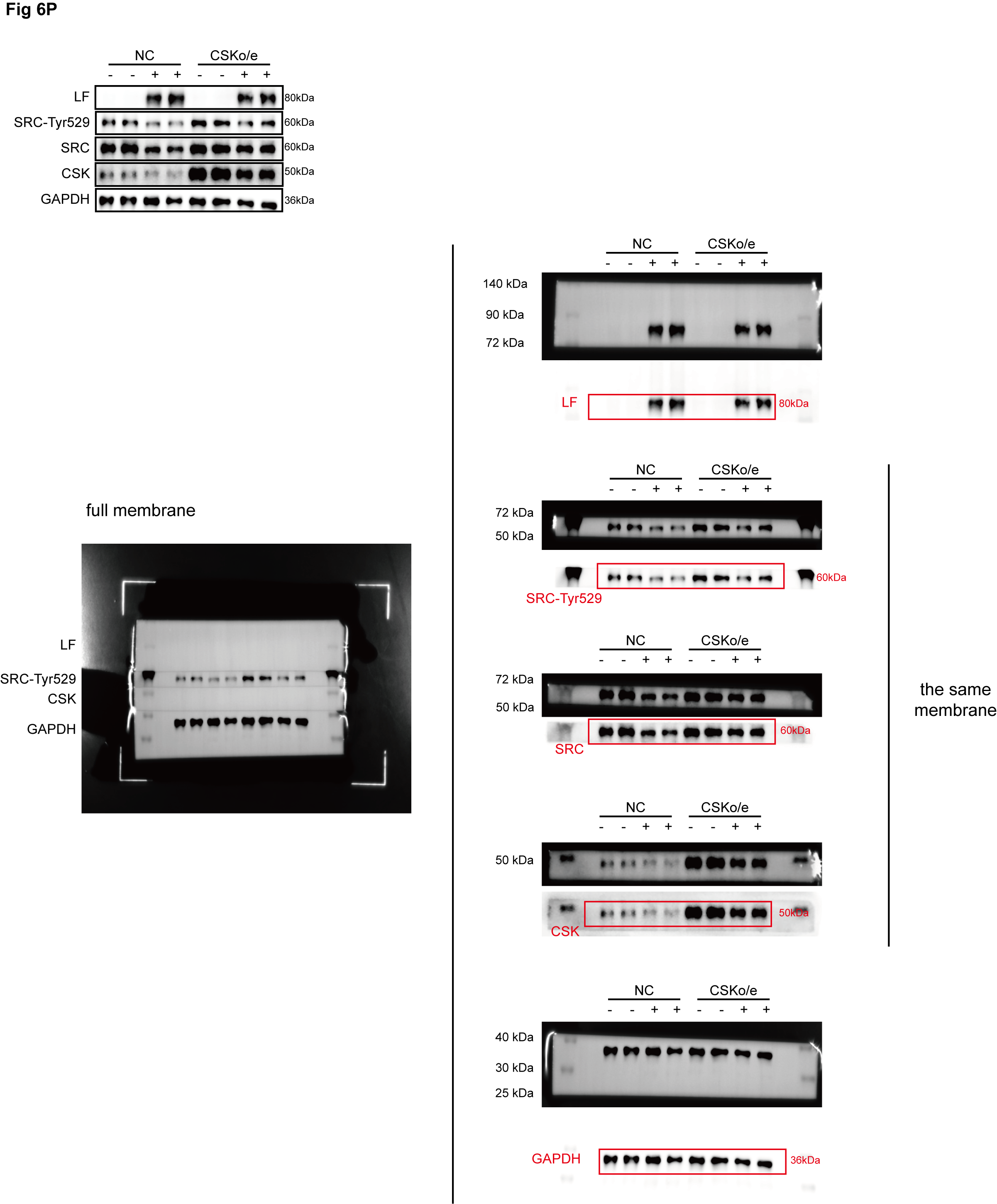


**Supplementary Figure S10**: Original images of blots corresponding to Figure 6P in the main text. Red boxes indicate the cropped areas shown in the main figure.


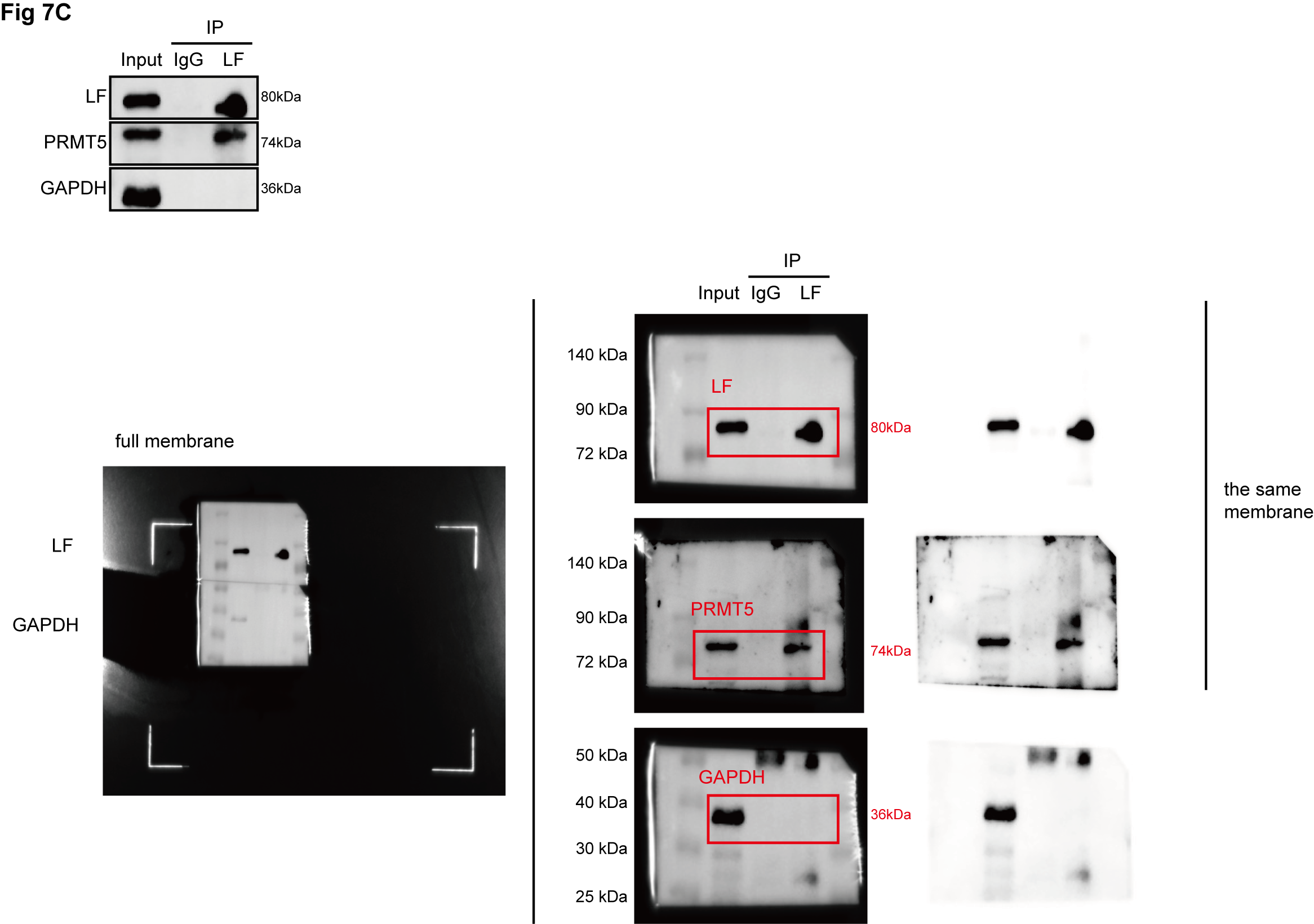


**Supplementary Figure S11**: Original images of blots corresponding to Figure 7C in the main text. Red boxes indicate the cropped areas shown in the main figure.


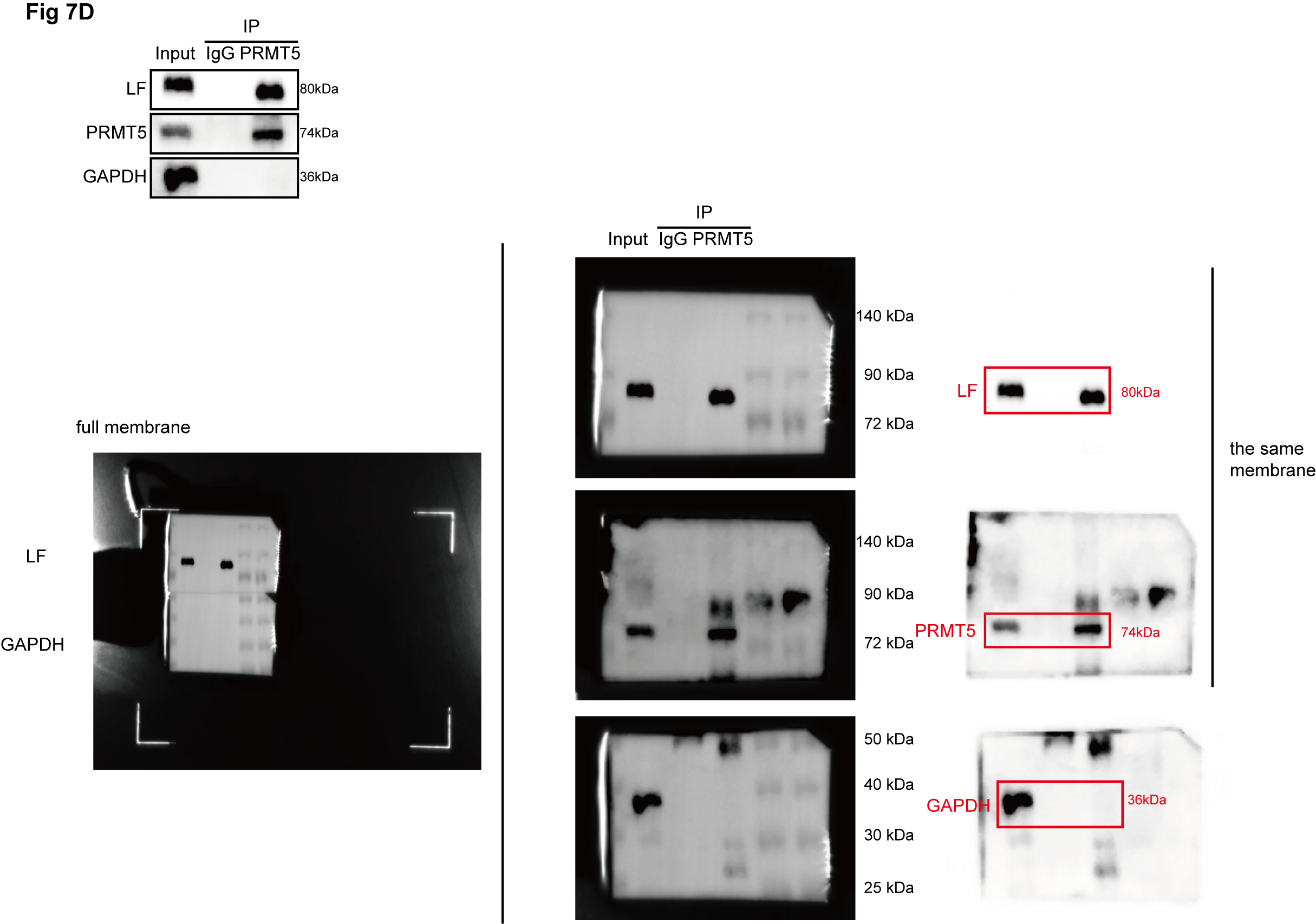


**Supplementary Figure S12**: Original images of blots corresponding to Figure 7D in the main text. Red boxes indicate the cropped areas shown in the main figure.


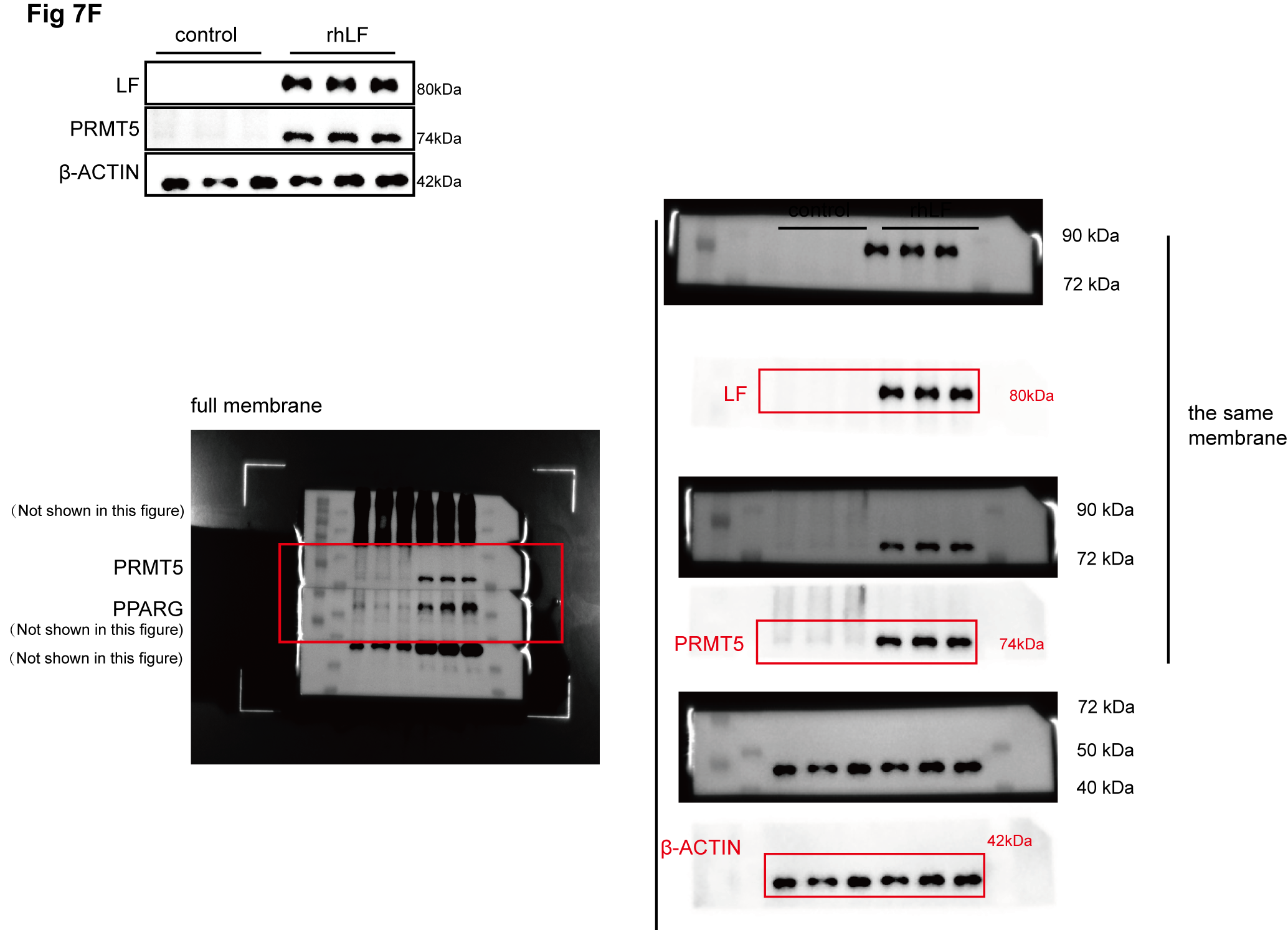


**Supplementary Figure S13**: Original images of blots corresponding to Figure 7F in the main text. Red boxes indicate the cropped areas shown in the main figure.


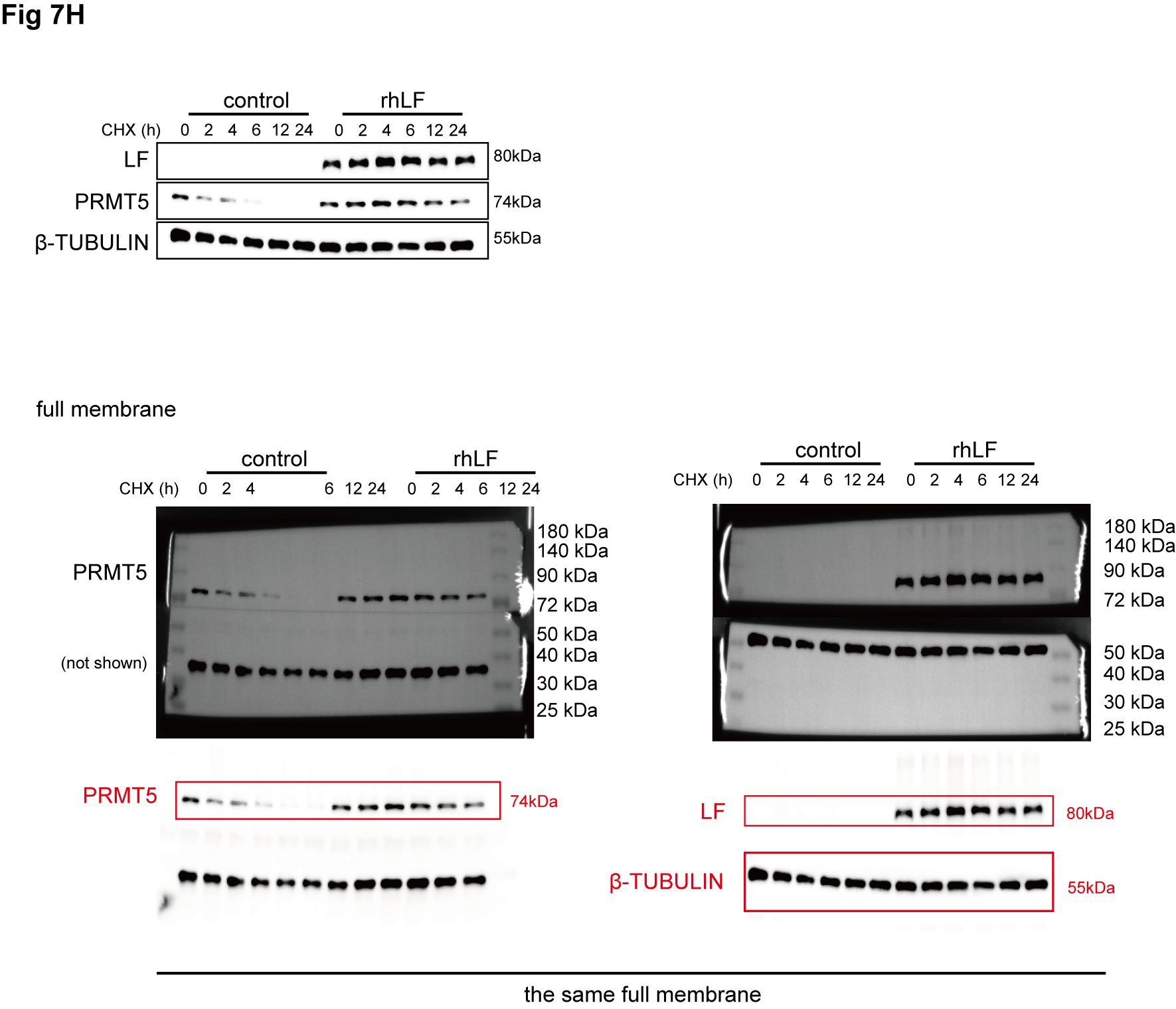


**Supplementary Figure S14**: Original images of blots corresponding to Figure 7H in the main text. Red boxes indicate the cropped areas shown in the main figure.


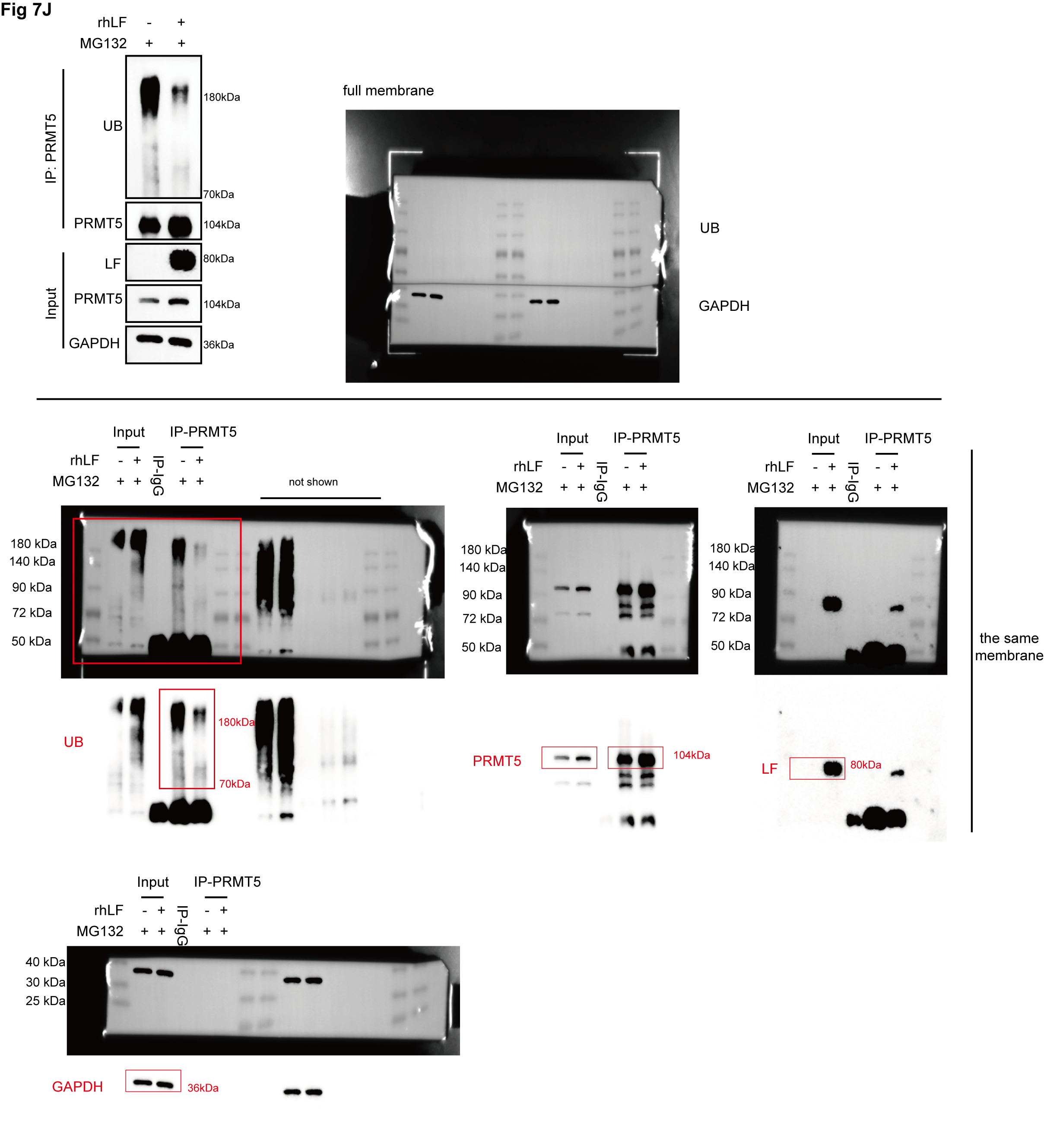


**Supplementary Figure S15**: Original images of blots corresponding to Figure 7J in the main text. Red boxes indicate the cropped areas shown in the main figure.


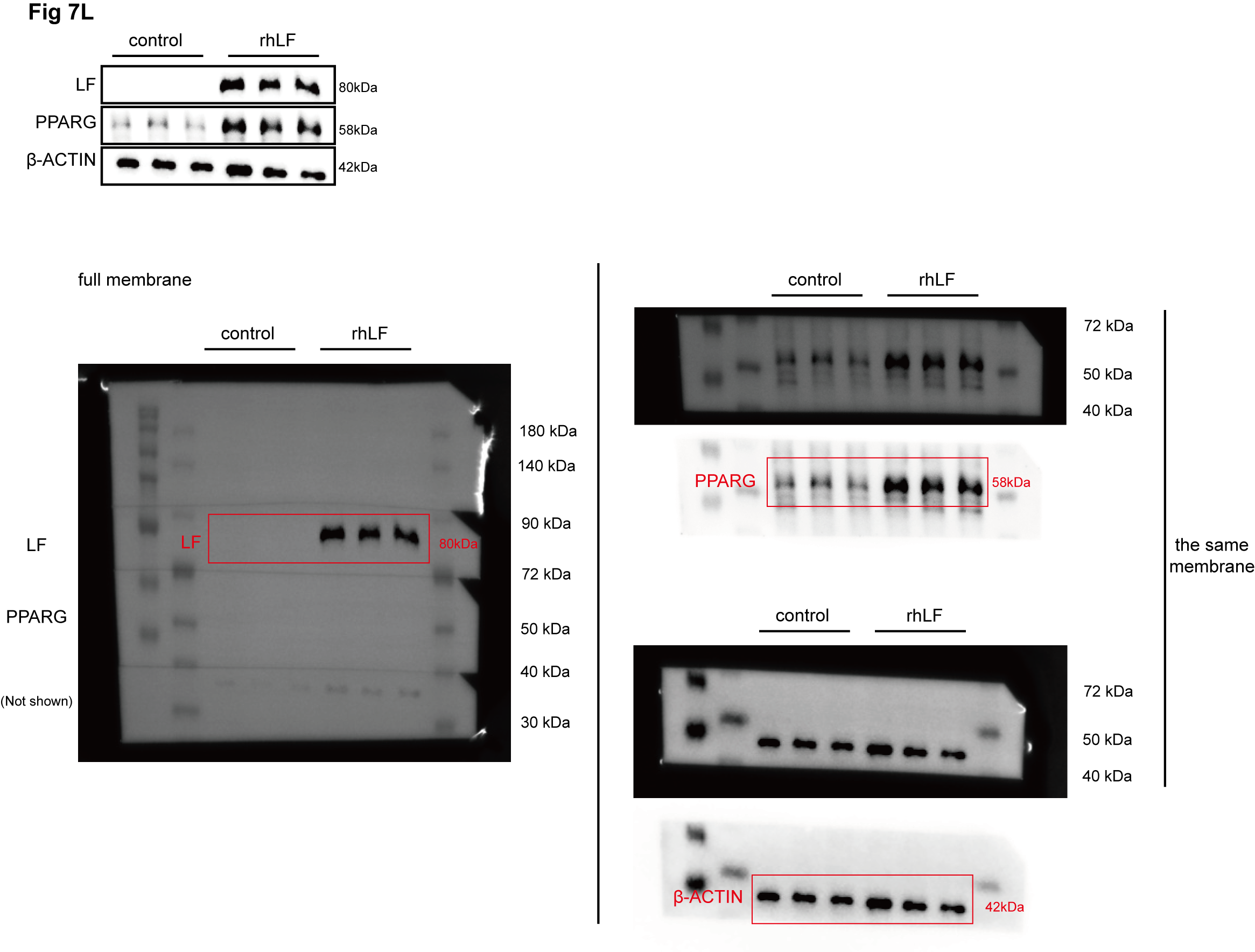


**Supplementary Figure S16**: Original images of blots corresponding to Figure 7L in the main text. Red boxes indicate the cropped areas shown in the main figure.


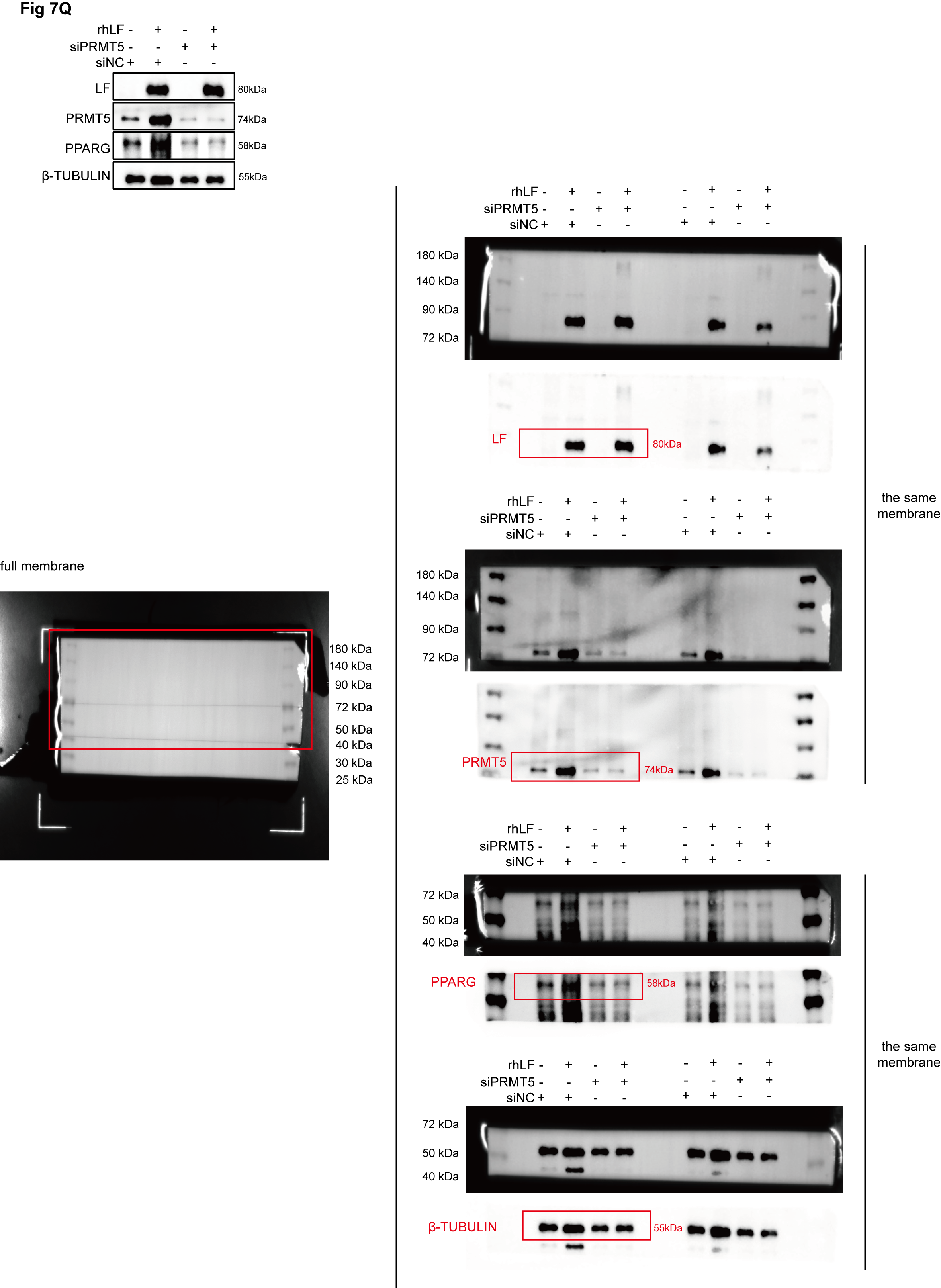


**Supplementary Figure S17**: Original images of blots corresponding to Figure 7Q in the main text. Red boxes indicate the cropped areas shown in the main figure.


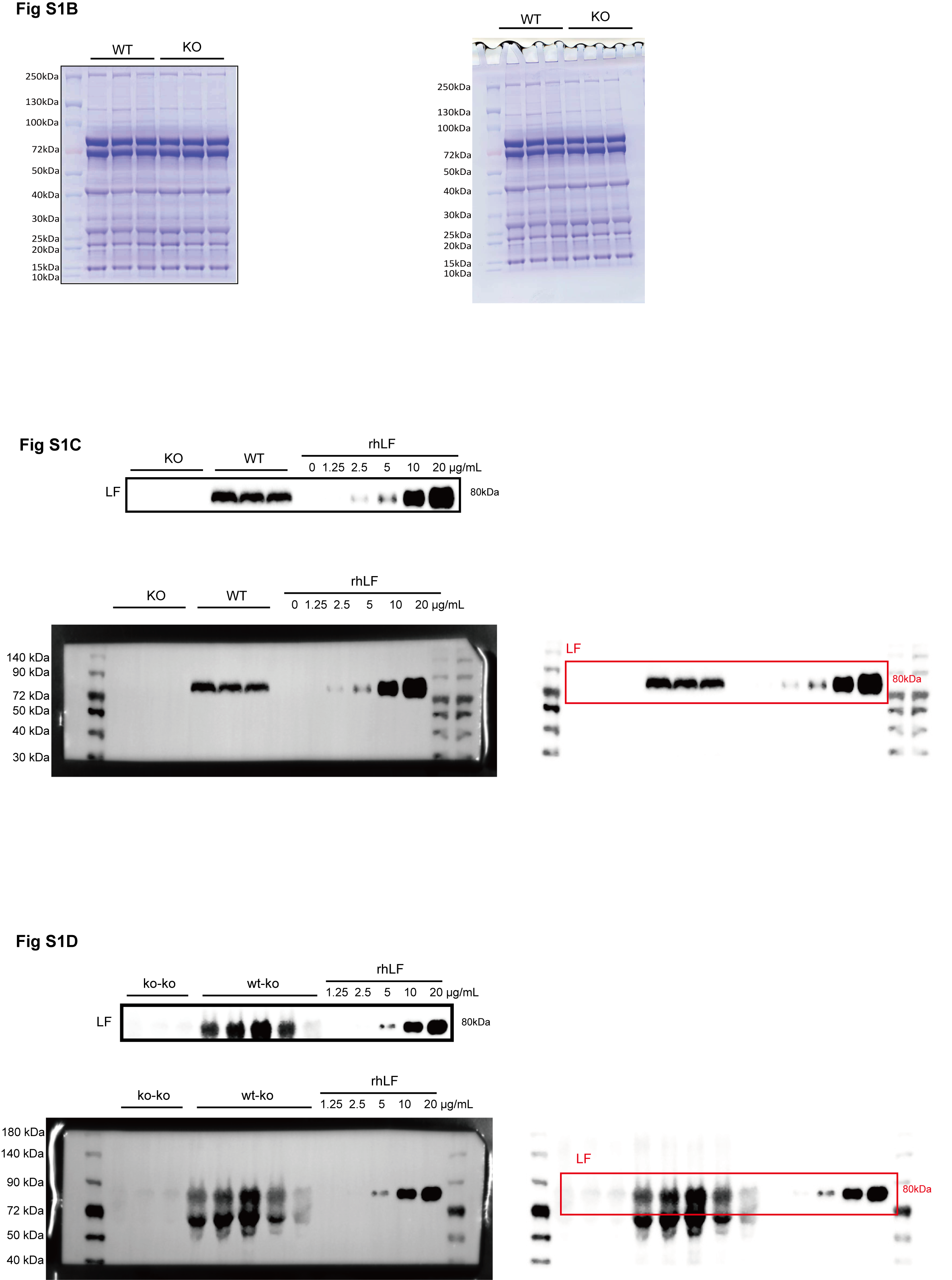


**Supplementary Figure S18**: Original images of blots corresponding to Figure S1B, Figure S1C and Figure S1D in the article. Red boxes indicate the cropped areas.


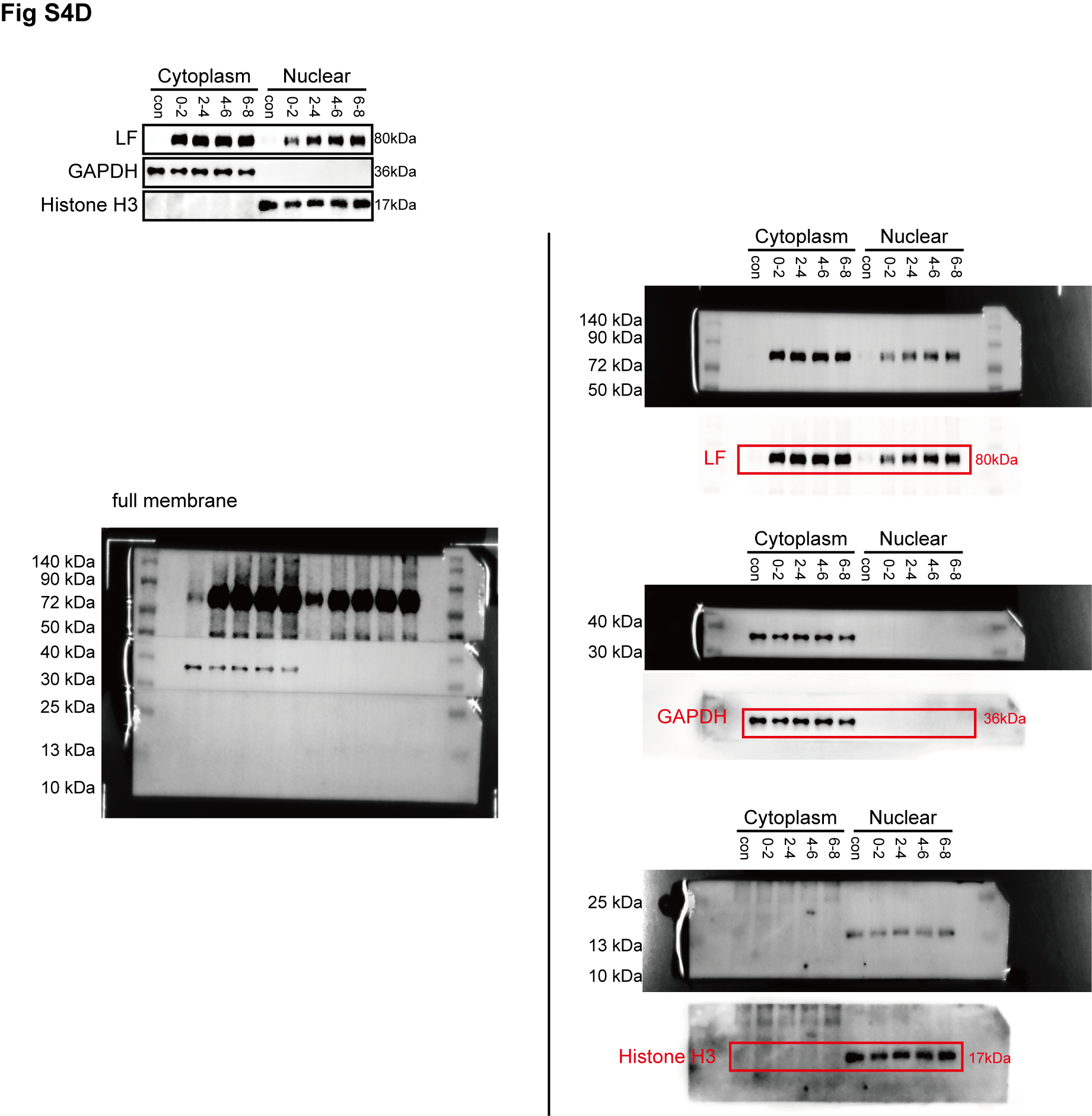


**Supplementary Figure S19**: Original images of blots corresponding to Figure S4D in the article. Red boxes indicate the cropped areas.


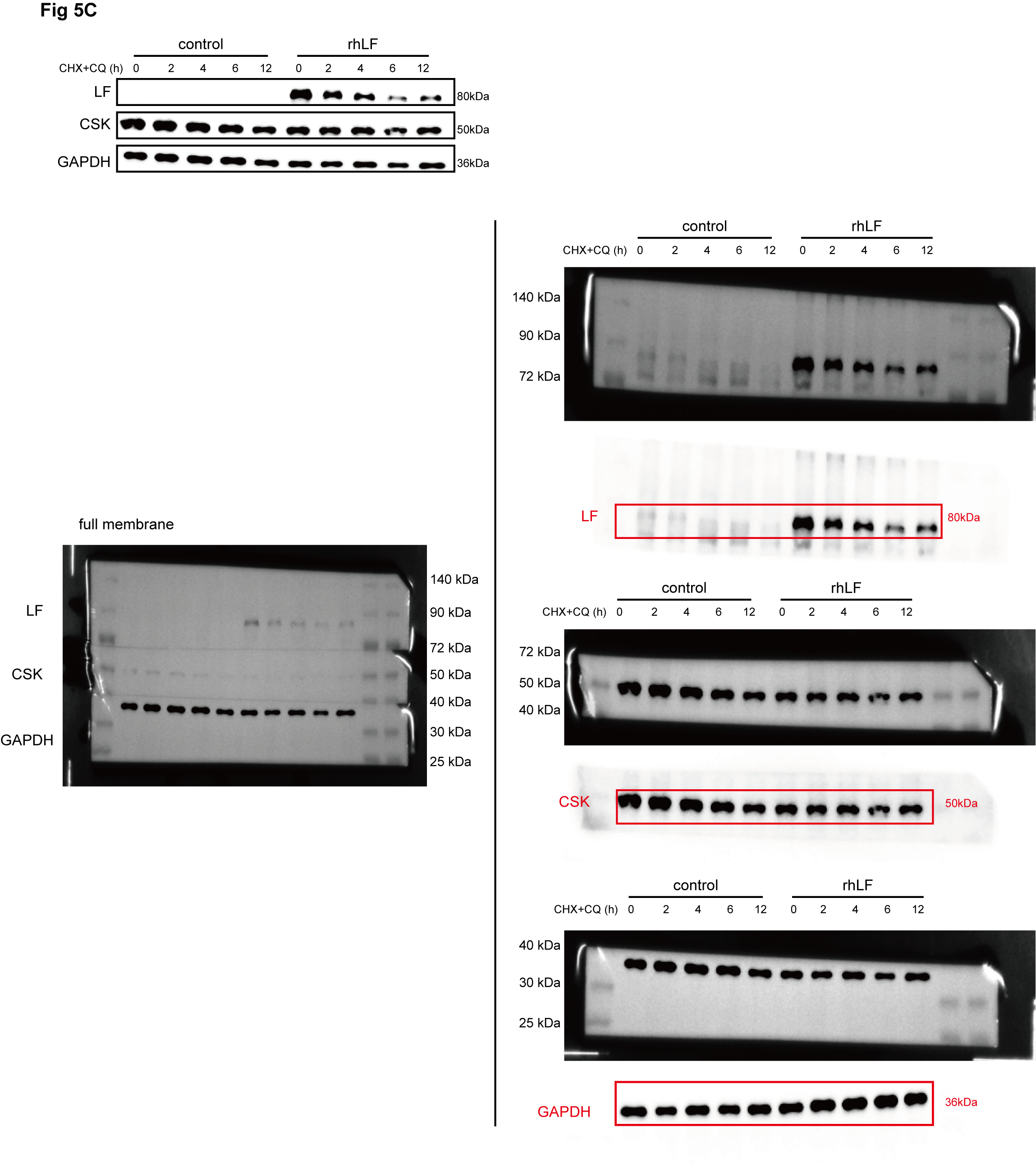


**Supplementary Figure S20**: Original images of blots corresponding to Figure S5C in the article. Red boxes indicate the cropped areas.


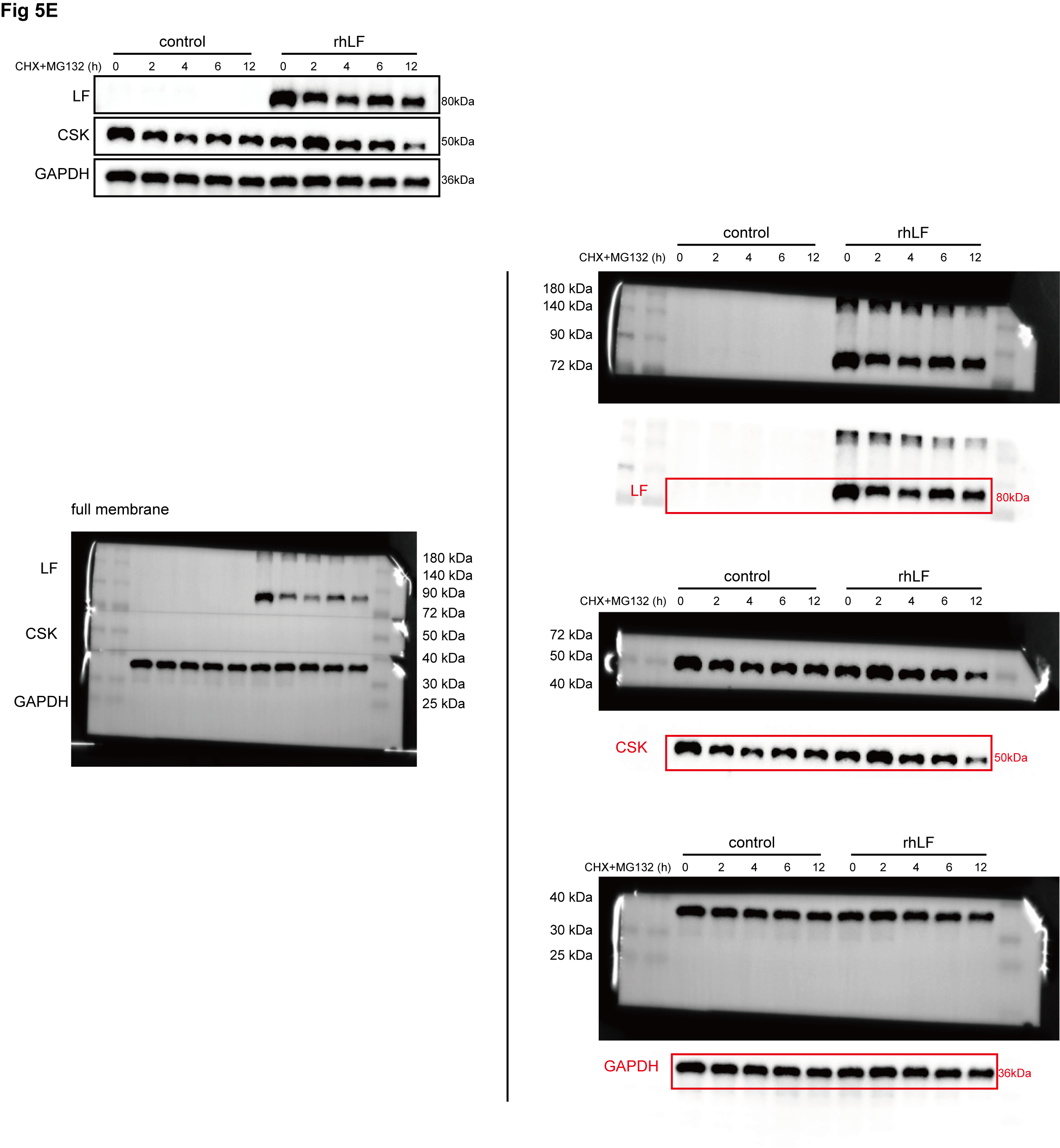


**Supplementary Figure S21**: Original images of blots corresponding to Figure S5E in the article. Red boxes indicate the cropped areas.
